# CD4^+^ T Cells Sensitize Quasimesenchymal Breast Tumors Lacking CD73 to Anti-CTLA4 Immune Checkpoint Blockade Therapy

**DOI:** 10.1158/2767-9764.CRC-26-0304

**Published:** 2026-06-02

**Authors:** Shiney Chandraganti, Caitlyn Sams, Sarthak Sahoo, Brian Feng, Isabel O’Connell, Lynna Li, Sunita Nepal, Siddhartha Pulukuri, Kimaya Bakhle, Prathapan Thiru, Corina Simian, Viviana Maymi, Brian D. Rudd, George W. Bell, Stephen Hatfield, Mohit Kumar Jolly, Anushka Dongre

**Affiliations:** 1 Department of Biomedical and Translational Sciences, College of Veterinary Medicine, Cornell University, Ithaca, New York.; 2 Department of Bioengineering, Indian Institute of Science, Bangalore, India.; 3 Whitehead Institute for Biomedical Research, Cambridge, Massachusetts.; 4 Institute for Plant-Human Interface, Northeastern University, Boston, Massachusetts.; 5 Department of Microbiology and Immunology, College of Veterinary Medicine, Cornell University, Ithaca, New York.; 6 New England Inflammation and Tissue Protection Institute, Northeastern University, Boston, Massachusetts.

## Abstract

Although immune checkpoint blockade (ICB) therapy has generated dramatic responses in certain cancer types, breast tumors are largely unresponsive. Epithelial–mesenchymal plasticity (EMP) leads to the assembly of an immunosuppressive tumor microenvironment and drives resistance of breast tumors to immunotherapies. Importantly, targeting CD73 completely sensitizes quasi-mesenchymal (qM) breast tumors to anti-CTLA4 ICB therapy. However, the mechanism(s) of sensitization remained unknown. We demonstrate that targeting CD73 in qM breast tumors sensitizes them to anti-CTLA4 ICB therapy in a CD4^+^ T cell–dependent manner. Moreover, EMP results in elevated expression of cancer cell–intrinsic CD73 in human triple-negative breast cancers. Given the ability of qM cancer cells to metastasize and resist multiple therapies, these findings can instruct the formation of translational strategies for the treatment of human breast cancers. These findings also bring to the forefront the attractive possibility of utilizing the phenotypic plasticity of cancer cells along with CD73 and CD4^+^ T cells as a predictive criterion for immunotherapy responsiveness.

**Significance:**

EMP enables metastasis and drives resistance to multiple treatment regimens. Our work underscores the possibility of utilizing CD4^+^ T cells, CD73, and cancer plasticity as predictive criteria for responsiveness to immunotherapy.

## Introduction

The use of immune checkpoint blockade (ICB) therapies, which harness the immune system to kill cancer cells, has revolutionized cancer treatment by creating durable clinical responses (1). However, whereas melanomas and lung cancers mount proficient responses to these therapies, certain other cancer types, such as breast carcinomas, are still largely unresponsive (2–5). It is therefore critical that we drastically improve the curative potential of these therapies by understanding the mechanisms by which breast cancer cells mount resistance to immunotherapy.

Epithelial–mesenchymal plasticity (EMP), which converts epithelial cells to mesenchymal derivatives, endows cancer cells with many traits associated with high-grade malignancies. This includes their ability to metastasize to distant organ sites, acquire tumor-initiating abilities, and mount resistance to chemotherapies (6–9). In fact, EMP is a highly dynamic process which often gives rise to a spectrum of partial or quasi-mesenchymal (qM) states which can coexpress both more-epithelial and more-mesenchymal markers (10–12). Importantly, EMP is a reversible process in which mesenchymal-like cancer cells can lapse back into an epithelial state by undergoing a mesenchymal-to-epithelial transition (13). In addition to these well-documented features of this program, we have shown that EMP can lead to the assembly of an immunosuppressive tumor microenvironment (TME) and render breast tumors unresponsive to ICB therapies (14–17).

In previous work, we established epithelial or qM cell lines from tumors arising in the MMTV-PyMT autochthonous murine model of breast cancer (18). Some of these mice contained IRES-YFP reporter constructs that labeled cells expressing the Snail epithelial–mesenchymal transition (EMT) transcription factor (TF), enabling us to isolate Snail^HI^ qM cancer cells which differed from their Epcam-expressing epithelial counterparts (18, 19). By implanting these cell lines into immunocompetent, syngeneic hosts, we established preclinical murine models of epithelial or qM breast tumors and observed that epithelial tumors recruit CD8^+^ T cells to the TME and are highly responsive to anti-CTLA4 ICB. In sharp contrast, qM tumors recruit immunosuppressive cells such as T-regulatory cells (Treg) and M2-like macrophages instead and are resistant to the same therapy (18). Most strikingly, in mixed tumors comprised of both epithelial and qM cancer cells, a minority population (10%) of more-mesenchymal cells can cross-protect the vast majority (90%) of their epithelial neighbors from immune attack (18). These observations alone are of great consequence clinically, as the majority of human carcinomas contain minority populations of more-mesenchymal cells that could dictate the outcome of the entire tumor to immune attack.

This ability of qM cancer cells to resist being eliminated by the immune system is in part due to their secretion of multiple immunosuppressive factors (20). Of particular importance is the observation that qM cancer cells that lack the expression of a specific immunosuppressive factor called CD73 (an ectoenzyme that produces immunosuppressive adenosine) were completely sensitized to anti-CTLA4 ICB (20). Additionally, targeting the adenosinergic signaling pathway in qM tumors with either anti-CD73 or an adenosine receptor antagonist generated synergistic responses, with anti-CTLA4 leading to a significant reduction in primary tumor size as well as distal metastases. Additionally, these strategies sensitized qM breast tumors specifically to anti-CTLA4 but not anti–PD-1 ICB therapy (20). Our previous work demonstrated for the very first time that disruption of certain cancer cell–intrinsic, EMP-regulated immunosuppressive signaling channels, notably CD73, could lead to a near-complete eradication of qM cancer cells.

Given that more-mesenchymal cells enable metastasis and are notoriously resistant to multiple treatment regimens, strategies to eliminate them altogether could revolutionize cancer treatment.

However, the mechanism(s) underlying such sensitization remains unknown. In other words, the identity of immune cell subsets that mediate the elimination of qM cancer cells lacking CD73 in response to anti-CTLA4 ICB treatment is yet to be determined. We present our findings demonstrating that CD4^+^ T cells sensitize qM breast tumors that lack the expression of CD73 to anti-CTLA4 ICB. Importantly, CD73 expression is associated with a partial and more-mesenchymal state in human breast cancer cell lines and patient samples. Our work brings to the forefront the attractive possibility of utilizing CD4^+^ T cells, CD73, and EMP as predictive criterion for ICB responsiveness of breast tumors.

## Materials and Methods

### Mice

C57BL/6J female mice (IMSR_JAX:000664), ages 6 to 8 weeks, were obtained from The Jackson Laboratory. Mice were age-matched and randomly assigned to treatment or control groups in all experiments. All animal procedures were carried out in compliance with guidelines and protocols approved by the Institutional Animal Care and Use Committee and maintained by the Center for Animal Resources and Education at Cornell University.

### Cell lines and tissue culture

All murine cell lines sgCD73, Snail^HI^ qM, CD73, and B2M double knockout (DKO) and MCF7RAS human breast cancer cells containing doxycycline-inducible Luciferase or SLUG and SOX9 constructs were a kind gift from the Weinberg Lab and established and maintained as previously described (18–20). Murine cell lines were cultured in a 1:1 mixture of Dulbecco modified Eagle medium (DMEM) and Ham’s F12 medium supplemented with 5% bovine adult serum, 1× penicillin–streptomycin, and 1× nonessential amino acids maintained at 37°C in an incubator containing 5% CO_2_. MCF7RAS cells were maintained in DMEM with 10% bovine fetal serum and 1× penicillin–streptomycin as previously described (18–20). All cell lines were kept for a maximum of 10 passages since thawing. All cell lines were routinely tested for *Mycoplasma* (from 2019–2025) using the MycoAlert Mycoplasma Detection Kit (Lonza). All cell lines were negative for *Mycoplasma* and not authenticated since they were first acquired.

### Generation of cell lines using CRISPR/Cas9

The sgCD73 cell line was established and maintained as previously described (20). In order to generate the CD73 and B2M DKO cell line, B2M was knocked out from the sgCD73 murine cell line via transient transfection using CRISPR/Cas9 plasmids obtained from Santa Cruz Biotechnology. Cells were initially seeded at 0.5 × 10^6^ cells/well in a six-well plate for 12 hours and transfected with the plasmid of interest according to the manufacturer’s protocol. After 48 hours of incubation, cells expressing GFP were sorted using (BD Biosciences FACSMelody) into each well of a 96-well plate to obtain single-cell clones. These single-cell clones were then expanded and screened for the presence or absence of B2M using Western blotting and for surface MHC-I by flow cytometry. B2M and MHC-I expression was measured on cell lines before and after treatment with 100 ng/mL of IFNγ for 48 hours.

### *In vivo* mouse models and tumor dissociation

For orthotopic tumor implantations, 1 × 10^6^ cells were counted and resuspended in 30 μL of media containing 20% Matrigel. Cells were then implanted orthotopically into the mammary fat pads of C57BL/6J mice. Tumor volume was calculated using the modified ellipsoid formula tumor volume (mm^3^) = (*L* × *W* × *W*)/2, in which *L* represents the largest tumor diameter and *W* represents the perpendicular measurement. After the tumors reached 2,000 mm^3^, the mice were sacrificed, and tumors were collected. A small section of the tumors was saved for fixing, whereas the rest was used for making single-cell suspensions for flow cytometry. For tumor digestions, tumors were cut and finely minced with a razor blade and digested in RPMI containing 2 mg/mL collagenase A (Krackeler Scientific) and 100 U/mL hyaluronidase (Krackeler Scientific). The suspension was then incubated in a rotator at 37°C for 40 minutes. After digestion, the single-cell suspension was filtered first through a 70-micron strainer and then through a 40-micron strainer, followed by centrifugation at 1,250 rpm for 10 minutes at 4°C. The pellets were then resuspended in RPMI containing monensin GolgiStop (BD Sciences) and incubated for 3 to 5 hours at 37°C. After incubation, the cells were centrifuged at 1,250 rpm for 10 minutes at 4°C and processed for flow cytometry as described in the following section.

### Flow cytometry analysis

The following steps were performed in 96-well V-bottom microwell plates using single-cell suspensions obtained after tumor processing. First, cells were centrifuged at 1,250 rpm for 5 minutes at 4°C, and the pellets were resuspended in 200 μL of FACS wash buffer with a master mix containing surface markers each diluted 1:100: CD3 FITC (17A2; BioLegend; AB_312660), TCR β-chain FITC (H57-597; BioLegend; AB_313428), CD107a (LAMP-1) PerCP-eFluor 710 (eBio 1DA; Invitrogen; AB_10718968), KLRG1 PerCP-eFluor 710 (13F12F2; Invitrogen; AB_2573888), CD279 (PD-1) APC (J43; Invitrogen; AB_11149860), CD45 BUV805 (30-F11; Invitrogen; AB_2925264), CD4 eFluor 450 (RM4-5; Invitrogen; AB_1272231), CD152 (CTLA4) eFluor 450 (UC10-49; BioLegend; AB_2564473), TIGIT PECY7 (1G9; BioLegend; AB_2565648), CD25 BV650 (PC61.5; BioLegend; AB_11125760), CD44 BV650 (IM7; BioLegend; AB_2562600), CD45 BUV395 (30-F11; BioLegend; AB_2925263), CD45 BUV563 (30-F11; BD Biosciences; AB_2722550), CD4 BUV805 (RM4-5; Invitrogen; AB_2925264), CD3 PE (145-2c11; Invitrogen; AB_465496), and CD8b (Ly-3) PE (YTS156.7.7; BioLegend; AB_961298) for 30 minutes in the dark on ice or at 4°C. Live/dead staining was performed for 15 to 20 minutes in the dark on ice or at 4°C using the Invitrogen LIVE/DEAD Fixable Near-IR Dead Cell Stain Kit with APC-CY7 dye. Cells were then fixed for 30 to 60 minutes using the Intracellular Fixation and Permeabilization Buffer Set (Thermo Fisher Scientific). Intracellular staining was performed for 30 to 60 minutes in the dark on ice or at 4°C using the FOXP3/Transcription Factor Staining Buffer Set (Thermo Fisher Scientific) for the following antibodies each diluted at 1:50 - FOXP3 FITC (FJK16s; BioLegend; AB_465242), CD152 (CTLA4) PerCP-eFluor 710 (UC10-4 B9; BioLegend; AB_2564473), TOX eFluor 660 (TXRX10; Invitrogen; AB_2574264), perforin APC (eBio0MAK-D; Invitrogen; AB_469514), TCF-7/TCF-1 BV421 (S33-966; BD Biosciences; AB_2869822), granzyme B BV421 (QA18A28; BioLegend; AB_2810602), CD366 (TIM3) Super Bright 702 (RMT3-23; BioLegend; AB_2744892), Ki67 BUV737 (SolA15; Invitrogen; AB_2896015), 4-1BB (CD137L) PE (TKS-1; BioLegend; AB_2256408), RORgt PerCP-eFluor 710 (B2D; Invitrogen; AB_10717534), TNFα BV650 (MP6-XT22; BioLegend; AB_2562450), IFNγ BV785 (XMG1.2; BioLegend; AB_11219004), IL-17a BUV395 (TC11-18H10; BD BioSciences; AB_2722575), EOMES PE CF 594 (X4-83; BD Biosciences; AB_2916484), granzyme A PECY7 (GzA-3G8.5; Invitrogen; AB_2573475), and T-BET PECY7 (4B10, BioLegend; AB_2561760). The single-stain controls were made using UltraComp eBeads Compensation Beads (Invitrogen). Flow cytometry data were acquired on a BD Biosciences FACSymphony A3, and data were analyzed using the FlowJo (TreeStar) software (SCR_008520).

### Antibody depletion and ICB treatment

For single or combinatorial treatment with immunotherapy, mice received 200 μg of anti-CTLA4 (clone 9H10, Bio X Cell; AB_10950184) and/or 100 μg of anti-CD73 (clone TY/23, Bio X Cell; AB_10972983) diluted in 200 μL of phosphate-buffered saline (PBS) administered intraperitoneally every other day for six doses followed by 100 μg weekly injections for seventh and eighth doses. Other depletion antibodies were administered as follows: 200 μg anti-CD4 (clone GK1.5, Bio X Cell; AB_1107642), 200 μg anti-CD8a (clone 53-6.7, Bio X Cell; AB_1107671), 200 μg of anti-NK1.1 (clone PK136, Bio X Cell; AB_10973500), and 200 μg of anti-CSF1R (clone AFS98, Bio X Cell; AB_2687699) and 200 μg of anti-γδ TCR (clone UC7-13D5, Bio X Cell; AB_1107751) administered once a week after 1 to 3 days of implantation until control tumors reached approximately 2,000 mm^3^ in size.

### Immunofluorescence staining

Tumor sections were fixed in 10% neutral buffered formalin for 24 to 48 hours and transferred to freshly made 70% ethanol, followed by embedding and sectioning. Formalin-fixed, paraffin-embedded (FFPE) tumor sections were deparaffinized in Histoclear and rehydrated through a series of decreasing ethanol concentrations (100%, 95%, and 75%, Milli-Q water and 1× DAKO wash buffer; 5 minutes each). For antigen retrieval, the section slides were immersed in 1× DAKO antigen retrieval buffer pH 6.0 (Agilent Technologies) and microwaved. Following this, the slides were washed twice with 1× DAKO wash buffer (Agilent Technologies). To block nonspecific binding, sections were blocked with PBS containing 0.3% Triton X-100 (Millipore Sigma) and 1% normal donkey serum (Jackson Immunoresearch Lab) for 20 minutes at room temperature (RT). After blocking, the sections were stained overnight at 4°C with anti-rabbit CD4 monoclonal antibody (mAb; 1:100 Abcam EPR19514) and anti-mouse CD8a (1:100 Thermo Fisher Scientific). The sections were then washed twice with 1× DAKO wash buffer and tagged with Biotium CF555 donkey anti-rabbit IgG (1:500) and Biotium CF488A donkey anti-mouse IgG (1:500) for 2 hours at RT. After three washes, the sections were stained with DAPI (1:1,000 from 10 mg/mL stock, Millipore Sigma) for 5 minutes at RT. Slides were washed for 5 minutes in distilled water and mounted with ProLong Gold Antifade mounting media (Cell Signaling Technologies 9071S).

### Microscopy

The stained sections were viewed under a Zeiss AxioObserver inverted microscope with 63× oil objective, and the images were analyzed using the ZEN imaging software.

### Western blots

Lysates were made in aqueous 1× RIPA lysis buffer (EMD Millipore Corp). The concentration of extracted protein was measured using the Pierce BCA protein assay (Thermo Fisher Scientific, 23227), and the optical density was measured on a BioTek Synergy 2 Microplate Reader at 562 nm. Data were analyzed on Gen5 1.11 software. Forty μg of protein was resolved using the NuPAGE Bis-Tris mini protein gels, 12%, 1 mm (Invitrogen) and transferred to a 0.2-micron Immobilon-P polyvinylidene difluoride membrane (Millipore Sigma) using a wet transfer. The blots were then blocked in 5% Blotto (Santa Cruz) containing 0.2% Tween-20 (Sigma Aldrich) in PBS. Membranes were then probed with primary antibodies (1:1,000) overnight, washed and incubated with horseradish peroxidase (HRP)-labeled secondary antibodies (1:5,000), and developed using Dura substrate (Thermo Fisher Scientific). Primary antibodies: β2-microglobulin (Abcam; AB_1523204), CD73 mAb (Invitrogen), GAPDH (AB_561053), E-cadherin (AB_2291471), Sox9 (AB_2665492), Slug (AB_2239535), and vimentin (AB_10695459; Cell Signaling Technologies); secondary antibodies: anti-rabbit IgG, HRP (AB_2099233) and anti-mouse IgG, HRP (AB_330924; Cell Signaling Technologies).

### Processing tumors for single-cell RNA sequencing

**Figure 1.**
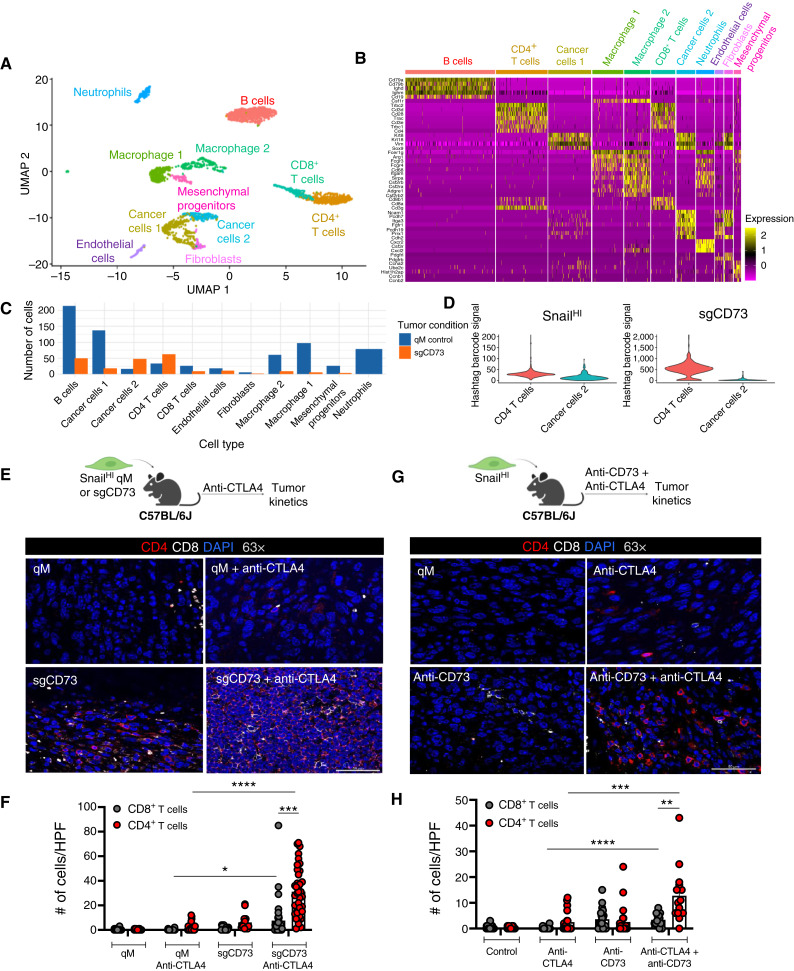
Presence of T cells in responding tumors. **A,** UMAP plot of unbiased clustering of Snail^HI^ qM and sgCD73 tumors, in which each color-coded cluster represents a specific cell type or state. **B,** Genes representing each cluster depicted in the UMAP plot in **A**. See additional details in Supplementary Table S1. **C,** Bar plot showing the number of cells per cluster assigned to Snail^HI^ qM control or sgCD73 conditions. **D,** Hashtag antibody-derived tag violin plots for the indicated clusters in Snail^HI^ qM control or sgCD73 conditions. **E,** Immunofluorescence analysis of primary tumor sections obtained from Snail^HI^ qM or sgCD73 tumor-bearing mice receiving control or anti-CTLA4 antibodies stained for CD4 (red), CD8 (white), and DAPI (blue) at 63× magnification. **F,** Quantification of CD8^+^ T cells and CD4^+^ T cells in each high-power field (HPF) at 63× magnification from **E**. Data represent three independent experiments, with *n* = 3–5 mice in each group. Fields of view (3–5) from each tumor interior were obtained for each group at 63× magnification. **G,** Immunofluorescence analysis of primary tumor sections obtained from Snail^HI^ qM tumor-bearing mice receiving control, anti-CD73, anti-CTLA4, or combinations of anti-CD73 and anti-CTLA4 antibodies stained for CD4 (red), CD8 (white), and DAPI (blue) at 63× magnification. **H,** Quantification of CD8^+^ T cells and CD4^+^ T cells in each HPF at 63× magnification from **G**. Data represent three independent experiments, with *n* = 3–5 mice in each group. Fields of view (3–5) from each tumor interior were obtained for each group at 63× magnification. **F** and **H,** Data represent the SEM, two-tailed unpaired *t* test, *, *P* < 0.05; **, *P* < 0.01; ***, *P* < 0.001; ****, *P* < 0.0001.

Tumors were processed into a single-cell suspension as described in the preceding sections and resuspended in 1× cold PBS. Two million cells were then resuspended in 100 μL of FACS wash buffer (0.05% bovine serum albumin in PBS). Each tumor sample was then incubated with the TotalSeq antibody cocktail (TotalSeq TM A0301 anti-mouse Hashtag 1 for Snail^HI^ qM tumors and Hashtag 2 for sgCD73 tumors; BioLegend clone M1/42; 30-F11) at a final concentration of 10 μg/mL. This was followed by a 30-minute incubation on ice, two washes with cold FACs buffer by centrifugation at 1,250 rpm for 10 minutes, followed by resuspension in PBS for a final count of 1 million cells. Each sample was filtered through a 40-micron filter and pooled. Libraries were generated using the 10X Genomics Chromium Single Cell 3 Prime Library Gel Bead Kit V2 followed by purification using solid-phase reversible immobilization beads and sequencing on an Illumina NextSeq system. After sequencing, the first step in processing the raw data was the demultiplexing of the samples based on the unique hashtag antibodies using Cell Ranger. Quality control (QC) measures were implemented to focus on biologically relevant, high-quality single cells. We removed cells with fewer than 500 features (e.g., the number of genes detected in a cell) and cells with more than 7,500 features, thereby excluding low-quality or nonviable cells and doublets. After QC, the estimated number of labeled cells was 6,332. Additionally, the percentage of mitochondrial gene expression in each cell was determined as part of the QC process. We used Loupe to identify the number of cells from each cluster. LogNormalize was applied to normalize the feature expression measurements in Seurat. Subsequent cell clustering was performed using the Seurat 5.0.1 package in R, utilizing the Louvain algorithm based on the elbow method. We selected 10 principal components for the analysis, which led to the identification of 11 clusters representing B cells, CD4^+^ T cells, CD8^+^ T cells, two subsets of macrophages, neutrophils, fibroblasts, endothelial cells, mesenchymal progenitor cells, and two subsets of cancer cells. Cluster identities were assigned based on known marker genes specific to each cell type. For each cell cluster, we identified 100 markers that defined the clusters through differential expression. A list of differentially expressed genes was compiled for each cluster, resulting in a heatmap that shows how the expression of specific clusters differed across samples (Fig. 1B). For example, genes such as CD79a, CD79b, and CD19 showed high expression in cluster 0, suggesting that this cluster was associated with B cells. Uniform Manifold Approximation and Projection (UMAP) was used to display the data.

### Computational analysis of publicly available murine tumors, human breast cancer cell lines, and patient data

Analysis of data from (21). Single-cell RNA sequencing (scRNA-seq) data processing and QC: Raw 10x Genomics count matrices for untreated and 7-day immunotherapy-treated breast cancer samples (KPB25Luv) were imported and processed using the scanpy (v1.9+) Python package. To ensure consistency, all gene symbols were converted to uppercase. Strict QC filters were applied to remove low-quality cells and potential doublets: cells expressing fewer than 200 or more than 5,000 unique genes were excluded, as were cells with greater than 10% mitochondrial gene content. Genes expressed in fewer than three cells were also removed from the dataset. Normalization, integration, and dimensionality reduction: Following QC, raw counts were library-size normalized to 10,000 counts per cell and log_1_p transformed. Highly variable genes (HVG) were identified using sample batch as a key, and the data were subsequently subsetted to these HVGs and scaled (maximum value = 10). To account for technical variation and effectively merge the untreated and treated biological states, principal component analysis was performed, followed by batch-effect correction using the Harmony algorithm (sc.external.pp.harmony_integrate). Clustering, signature scoring, and cell type annotation: A k-nearest neighbors graph was constructed using the first 40 Harmony-adjusted principal components and 10 neighbors. Cells were embedded into a two-dimensional (2D) space using UMAP and clustered using the Leiden algorithm (resolution = 0.8). Gene signature scoring, including the partial EMT (pEMT) signature (22), was calculated using the AUCell algorithm implemented via the decoupler package with a custom curated GMT file. Distinct cell types and states were annotated based on the expression of canonical marker genes identified via Wilcoxon rank-sum tests; the specific markers utilized for the identification of epithelial, immune, stromal, and endothelial lineages are detailed in the accompanying Supplementary Table S2. Pathway scores for all pathways were computed using AUCell scoring on different gene lists for different biological pathways considered (23). Spearman correlation was performed to assess the degree of correlation between CD73 expression and the different pathway scores calculated for the bulk RNA-seq data from the Cancer Cell Line Encyclopedia (CCLE). Luminal and basal gene expression signatures were obtained from (24). Epithelial and mesenchymal signatures were obtained from (22). Hallmark EMT signatures were obtained from MSigDB (25). pEMT signature was obtained from (26). The scores along the luminal–basal and epithelial–mesenchymal axes were calculated as described in (24).

### Statistical analysis

All statistics were performed using the GraphPad Prism v10 software. All data represent standard error of the mean using either two-tailed unpaired *t* tests or a regular two-way ANOVA with Tukey's multiple comparisons test. Asterisks indicate statistical significance where *, *P* < 0.05; **, *P* < 0.01; ***, *P* < 0.001; ****, *P* < 0.0001.

## Results

### Presence of T cells in responding tumors

In previous work, we established E and qM breast cancer cell lines by sorting cells from tumors arising in the autochthonous MMTV-PyMT murine model bearing Snail-IRES-YFP knock-in constructs (18). We have demonstrated that qM cancer cells lacking the expression of CD73 (hereafter referred to as sgCD73) are completely eliminated after treatment with anti-CTLA4 ICB relative to qM control tumors which are resistant (Supplementary Fig. S1A and S1B; ref. 20). However, precisely which immune cells mediate this elimination was unknown. We first determined how the absence of CD73 from qM cancer cells alters the TME even before the administration of anti-CTLA4 ICB by performing multiplexed scRNA-seq analysis of qM control and sgCD73 tumors. We identified 11 clusters representing B cells, CD4^+^ T cells, CD8^+^ T cells, two subsets of macrophages, neutrophils, fibroblasts, endothelial cells, mesenchymal progenitor cells, and two types of cancer cells (Fig. 1A and B; Supplementary Table S1). The relative abundance of cells within each cluster revealed that sgCD73 tumors contained elevated numbers of CD4^+^ T cells and a subset of cancer cells relative to qM control tumors even prior to ICB treatment (Fig. 1C). Hashtag antibody-derived tag violin plots demonstrate robust demultiplexing, with CD4^+^ T cells exhibiting strong, condition-specific barcode enrichment and minimal cross-sample contamination (Fig. 1D). We have previously determined that abrogation of CD73 from qM cancer cells results in an immunopermissive TME with an influx of T cells and fewer Tregs and M2-like macrophages relative to the immunosuppressive TME of control qM tumors (20). Consistent with these findings, a closer analysis of the CD4^+^ T-cell cluster revealed that although sgCD73 tumors contained greater numbers of CD4^+^ T cells, there were fewer immunosuppressive Tregs within this subset relative to control qM tumors (Supplementary Fig. S1C and S1D).

Given the ability of anti-CTLA4 ICB to regulate the recruitment and function of both CD8^+^ and CD4^+^ T cells, we focused our analyses on T cells and asked whether the proportion of both these subsets was altered in sgCD73 and qM tumors after treatment with anti-CTLA4 ICB. Immunofluorescence staining of tumor sections revealed that responding tumors (sgCD73 treated with anti-CTLA4) recruited significantly higher numbers of both CD8^+^ and CD4^+^ T cells to the tumor core relative to control qM tumors that were unresponsive to ICB therapy (Fig. 1E and F; Supplementary Fig. S1A and S1B). We have previously determined that treating Snail^HI^ qM tumor-bearing mice with anti-CD73 in combination with anti-CTLA4 ICB generates synergistic responses resulting in significantly smaller tumors in approximately 75% of recipients relative to mice receiving each antibody individually (Supplementary Fig. S1E and S1F; ref. 20). Accordingly, Snail^HI^ qM tumor-bearing mice that received combinations of anti-CD73 and anti-CTLA4 also demonstrated an increased influx of both CD8^+^ and CD4^+^ T cells relative to untreated tumors or those that received each therapy individually (Fig. 1G and H). What was particularly striking in both models was that although responding tumors recruited both T-cell types, the number of CD4^+^ T cells in the tumor core was significantly higher than the number of CD8^+^ T cells (Fig. 1F and H).

### CD8^+^ T cells are partially important for regulating responses of qM tumors lacking CD73 to anti-CTLA4 ICB

**Figure 2.**
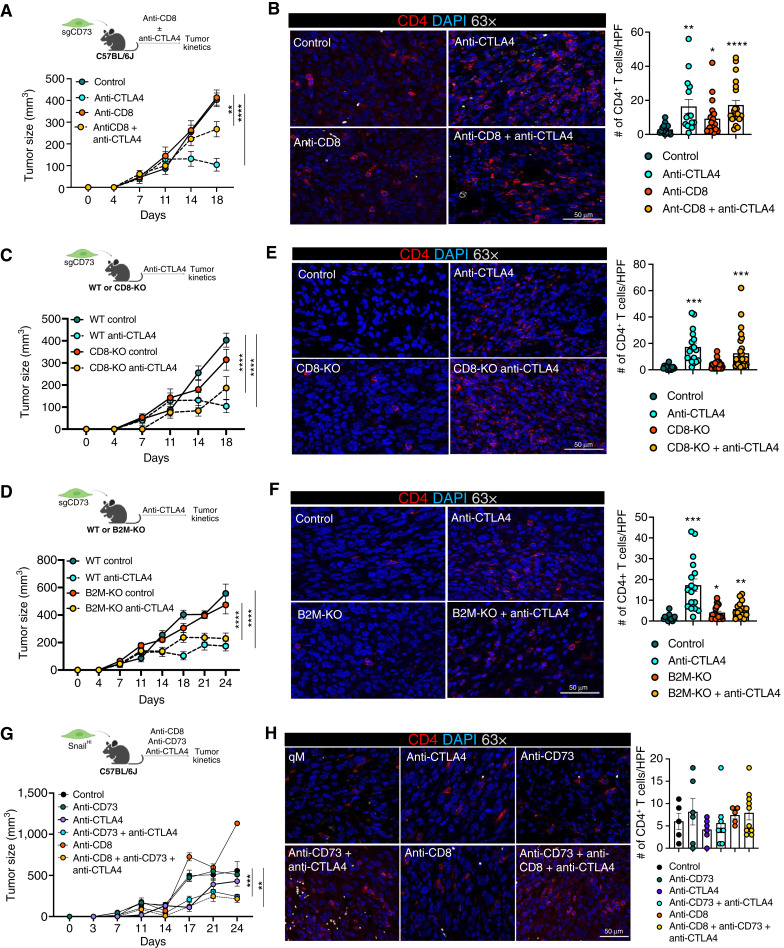
CD8^+^ T cells are partially important for sensitizing qM tumors lacking CD73 to anti-CTLA4 ICB. **A,** Schema and tumor kinetics for sgCD73 tumor-bearing mice treated with the indicated antibodies. Data represent three independent experiments in which *n* = 3–4 for each group. **B,** Representative immunofluorescence images of primary tumor sections obtained from (**A**) stained for CD4 (red) and DAPI (blue) at 63× magnification. Bar graph on the right represents quantification of CD4^+^ T cells in each high-power field (HPF) at 63× magnification for the indicated treatment groups. Fields of view (3–5) from the tumor interior were obtained for each tumor. Data represent three independent experiments, with *n* = 3–5 mice in each group. **C,** Schema and tumor kinetics for sgCD73 tumors propagated in WT or CD8-KO mice treated with the indicated antibodies. Data represent three independent experiments in which *n* = 3–4 for each group. **D,** Schema and tumor kinetics for sgCD73 tumors propagated in WT or B2M-KO mice treated with the indicated antibodies. Data represent three independent experiments in which *n* = 3–5 for each group. **E,** Representative immunofluorescence images of primary tumor sections obtained from (**C**) stained for CD4 (red) and DAPI (blue) at 63× magnification. Bar graph on the right represents quantification of CD4^+^ T cells in each HPF at 63× magnification for the indicated treatment groups. Fields of view (3–5) from the tumor interior were obtained for each tumor. Data represent three independent experiments, with *n* = 3–5 mice in each group. **F,** Representative immunofluorescence images of primary tumor sections obtained from (**D**) stained for CD4 (red) and DAPI (blue) at 63× magnification. Bar graph on the right represents quantification of CD4^+^ T cells in each HPF at 63× magnification for the indicated treatment groups. Fields of view (3–5) from the tumor interior were obtained for each tumor. Data represent three independent experiments, with *n* = 3–5 mice in each group. **G,** Schema and tumor kinetics for Snail^HI^ qM tumor-bearing mice treated with the indicated antibodies. Data represent two independent experiments in which *n* = 3–4 for each group. **H,** Representative immunofluorescence images of primary tumor sections obtained from (**G**) stained for CD4 (red) and DAPI (blue) at 63× magnification. Bar graph on the right represents quantification of CD4^+^ T cells in each HPF at 63× magnification for the indicated treatment groups. Fields of view (3–5) from the tumor interior were obtained for each tumor. Data represent three independent experiments, with *n* = 3–5 mice in each group. **A**, **C**, **D**, and **G,** Data represent the SEM, two-way ANOVA, **, *P* < 0.01; ***, *P* < 0.001; ****, *P* < 0.0001. **B**, **E**, **F**, and **H,** Bar graph data represent the SEM, two-tailed unpaired *t* test, *, *P* < 0.05; **, *P* < 0.01; ***, *P* < 0.001; ****, *P* < 0.0001. Scale bars are 100 μm.

To understand which T-cell subset was functionally important in regulating responses of sgCD73 tumors to anti-CTLA4 ICB, we first depleted CD8^+^ T cells using subset-specific antibodies. Depletion of CD8^+^ T cells from sgCD73 tumor-bearing mice only partially reversed sensitivity to anti-CTLA4 ICB (Fig. 2A; Supplementary Fig. S1G; ref. 20). Moreover, sgCD73 tumor-bearing mice continued to recruit CD4^+^ T cells to their primary tumors in response to anti-CTLA4 ICB, even in the absence of CD8^+^ T cells (Fig. 2B).

To validate these findings further, we implanted sgCD73 cells into genetically modified mice that lacked all CD8^+^ T cells (CD8-KO) or, alternatively, in mice which lacked B2M (B2M-KO). B2M is required for the stable cell-surface expression of MHC-I, which is critical for antigen presentation to CD8^+^ T cells. Thus, in this latter scenario, CD8^+^ T cells, although still present, are functionally compromised because of the absence of priming. Strikingly, sgCD73 tumors propagated in both CD8-KO and B2M-KO mice continued to respond to anti-CTLA4 ICB just as well as those propagated in wild-type (WT) mice that also received ICB treatment (Fig. 2C and D; Supplementary Fig. S1H and S1I). Additionally, a large number of CD4^+^ T cells were found within the tumor core of responding tumors that grew in both CD8-KO and B2M-KO mice upon treatment with anti-CTLA4 ICB, suggesting that the absence or functional impairment of CD8^+^ T cells did not affect the recruitment of CD4^+^ T cells to responding tumors (Fig. 2E and F). We then asked whether synergistic responses observed by using anti-CD73 in combination with anti-CTLA4 were also dependent on CD8^+^ T cells. Antibody-based depletion of CD8^+^ T cells from Snail^HI^ qM tumor-bearing mice did not alter their responses to combination therapy (anti-CD73 and anti-CTLA4). In other words, these tumor-bearing mice mounted synergistic responses to combination therapy and recruited CD4^+^ T cells to the tumor core just as efficiently in the presence and absence of CD8^+^ T cells (Fig. 2G and H; Supplementary Fig. S1J).

Finally, a closer analysis of CD8^+^ T-cell subsets revealed that responding tumors (sgCD73 treated with anti-CTLA4) had significantly more CD8^+^ T cells expressing markers of activation and proliferation (CD44 and Ki67; Supplementary Fig. S2A and S2B) relative to qM nonresponders. However, we observed no differences between other markers of immune functionality (CD107a, Eomes, perforin, IFNγ, granzyme A, granzyme B, and TNFα; Supplementary Fig. S2C–S2I) and exhaustion (TIGIT, PD-1, CTLA4, TCF1, and TIM3; Supplementary Fig. S2J–S2N) expressed by CD8^+^ T cells present in qM control and sgCD73 tumors before and after treatment with anti-CTLA4 ICB. Taken together, our data demonstrate that targeting CD73 sensitizes qM tumors to anti-CTLA4 in a manner that is only partially dependent on CD8^+^ T cells. Moreover, the absence of CD8^+^ T cells does not impact the recruitment of CD4^+^ T cells to responding tumors.

### CD4^+^ T cells drive sensitivity of qM tumors lacking CD73 to anti-CTLA4 ICB

**Figure 3.**
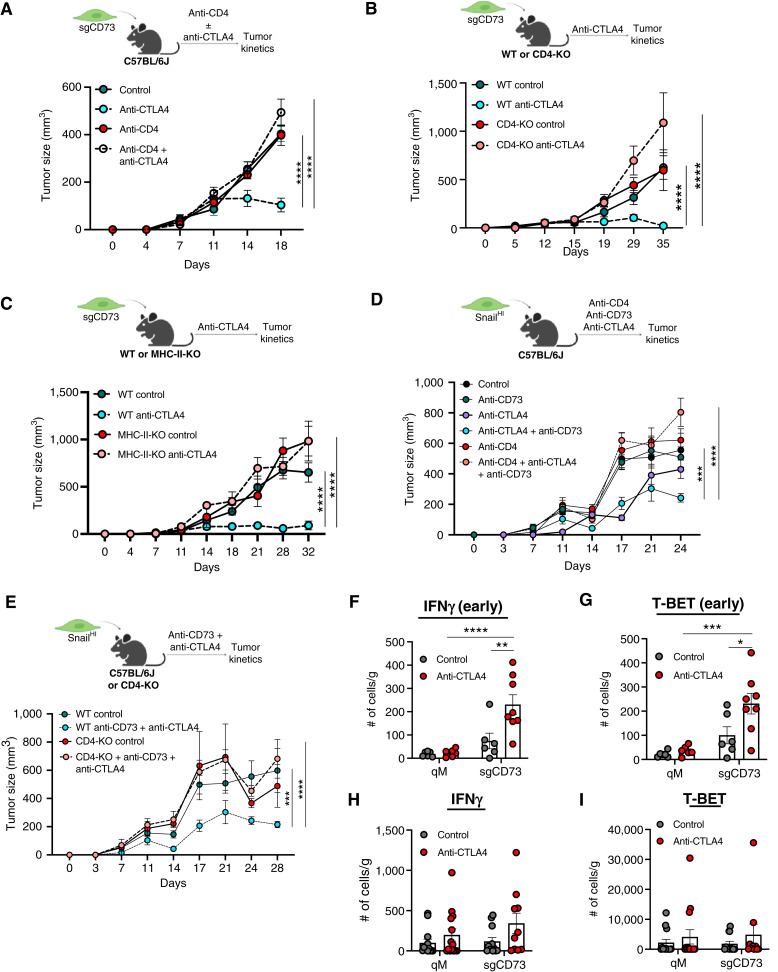
CD4^+^ T cells drive sensitization of qM tumors lacking CD73 to anti-CTLA4 ICB. **A,** Schema and tumor kinetics for sgCD73 tumor-bearing mice treated with the indicated antibodies. Data represent three independent experiments in which *n* = 3–7 for each group. **B,** Schema and tumor kinetics for sgCD73 tumors propagated in WT or CD4-KO mice treated with the indicated antibodies. Data represent three independent experiments in which *n* = 3–7 for each group. **C,** Schema and tumor kinetics for sgCD73 tumors propagated in WT or MHC-II-KO mice treated with the indicated antibodies. Data represent three independent experiments in which *n* = 3–7 for each group. **D,** Schema and tumor kinetics for Snail^HI^ qM tumor-bearing mice treated with the indicated antibodies. Data represent three independent experiments in which *n* = 3–7 for each group. **E,** Schema and tumor kinetics for Snail^HI^ qM tumors propagated in WT or CD4-KO mice treated with the indicated antibodies. Data represent three independent experiments in which *n* = 3–7 for each group. **F–I,** Flow cytometry analysis for (**F** and **H**) IFNγ and (**G** and **I**) T-BET expression on CD4^+^ T cells as determined by flow cytometry from Snail^HI^ qM control or sgCD73 tumor-bearing mice treated with the indicated antibodies. Tumors were harvested at 2 weeks (**F** and **G**) or 4 weeks (**H** and **I**) after treatment. Data represent three independent experiments in which *n* = 3–8 for each group. **A–E,** Data represent the SEM, two-way ANOVA, **, *P* < 0.01; ***, *P* < 0.001; ****, *P* < 0.0001. **F–I,** Data represent the SEM, two-way ANOVA, with Tukey's multiple comparisons test *, *P* < 0.05; **, *P* < 0.01; ***, *P* < 0.001; ****, *P* < 0.0001.

Although CD8^+^ T cells have been ascribed as the key players of the adaptive immune system in driving antitumor immune responses, the functional importance of antitumor CD4^+^ T cells is only beginning to emerge. Given the large influx of CD4^+^ T cells in both responding tumor models, we asked whether they were functionally important for sensitizing qM tumors lacking CD73 to anti-CTLA4 ICB. Antibody-based depletion of CD4^+^ T cells from sgCD73 tumor-bearing mice completely reversed their responsiveness to anti-CTLA4 ICB (Fig. 3A; Supplementary Fig. S3A; ref. 20).

Similarly, sgCD73 tumors failed to respond to ICB when grown in genetically modified mice that lacked all CD4^+^ T cells (CD4-KO; Fig. 3B; Supplementary Fig. S3B) relative to WT control mice. CD4^+^ T cells are activated when their T-cell receptor recognizes antigens presented by MHC-II molecules. Along these lines, genetically modified mice lacking the expression of MHC-II molecules have impaired CD4^+^ T-cell responses. To further confirm the functional importance of CD4^+^ T cells in driving sensitization of sgCD73 tumors to anti-CTLA4 ICB, we propagated sgCD73 cancer cells in WT or MHC-II-KO mice. Strikingly, sgCD73 tumors failed to respond to anti-CTLA4 ICB even when propagated in MHC-II-KO mice, in sharp contrast to WT mice, in which they mounted proficient responses to the same treatment (Fig. 3C; Supplementary Fig. S3C).

Finally, Snail^HI^ qM tumor-bearing mice failed to respond to combination therapy of anti-CD73 and anti-CTLA4 ICB when CD4^+^ T cells were depleted (Fig. 3D; Supplementary Fig. S3D). Similarly, Snail^HI^ qM tumors failed to respond to combinations of anti-CD73 and anti-CTLA4 when orthotopically implanted in CD4-KO mice (Fig. 3E; Supplementary Fig. S3E) relative to WT mice, in which they responded proficiently to combination therapy. Taken together, these findings from multiple models enabled us to determine that CD4^+^ T cells are necessary for sensitizing qM breast tumors lacking CD73 to anti-CTLA4 ICB.

CD4^+^ T cells are heterogeneous and can differentiate into multiple subsets, including antitumor, immunostimulatory T_H_1 and T_H_17 cells or protumor, immunosuppressive T_H_2 and Tregs (27). Whether the abrogation of CD73 from qM cancer cells altered the representation of one or more T-cell subsets remained unknown. This is particularly important as the polarization status of CD4^+^ T cells can directly influence breast tumor progression and could have profound consequences on their subsequent responsiveness to ICB therapies. Thus, we determined the identities of various CD4^+^ T-cell subsets in control qM and sgCD73 tumors before and after treatment with anti-CTLA4 ICB. sgCD73 tumors demonstrated a significant increase in the absolute numbers of IFNγ and T-BET–expressing CD4^+^ T cells only in response to anti-CTLA4 ICB 2 weeks after treatment relative to untreated tumors or qM tumors that were unresponsive to ICB treatment (Fig. 3F and G). However, these significant differences were no longer observed at later time points (4 weeks after treatment; Fig. 3H and I). Additionally, the representation of other T-cell subsets T_H_2, T_H_17, Tregs, T-follicular helper cells, as well as CD4^+^ T cells expressing granzyme A, granzyme B, or TNFα was unaltered in responders and nonresponders (Supplementary Fig. S4A–S4Q) before and after anti-CTLA4 ICB therapy. Moreover, Snail^HI^ qM and sgCD73 tumors did not show statistically significant differences in the proportion of CD4^+^ T cells expressing markers associated with proliferation or dysfunction such as KLRG1, Ki67, CTLA4, PD-1, TIGIT, TIM3, and 4-1BB (Supplementary Fig. S5A–S5H). Thus, sgCD73 tumors demonstrate elevated numbers of T_H_1-like CD4^+^ T cells at early time points after treatment with anti-CTLA4 ICB.

### Abrogation of MHC-I from qM tumors lacking CD73 drives sensitivity to anti-CTLA4 ICB in a CD4^+^ T cell–dependent manner

We and others have previously demonstrated that qM cancer cells significantly reduce their cell surface expression of MHC-I as a consequence of activating the EMP program, which could in turn render sgCD73 tumors vulnerable to NK cell–mediated cytotoxicity (20). Moreover, myeloid cells are also capable of engulfing MHC-deficient tumors. Thus, sensitization of sgCD73 tumors to anti-CTLA4 ICB could be driven by NK and/or myeloid cells, in addition to CD4^+^ T cells.

**Figure 4.**
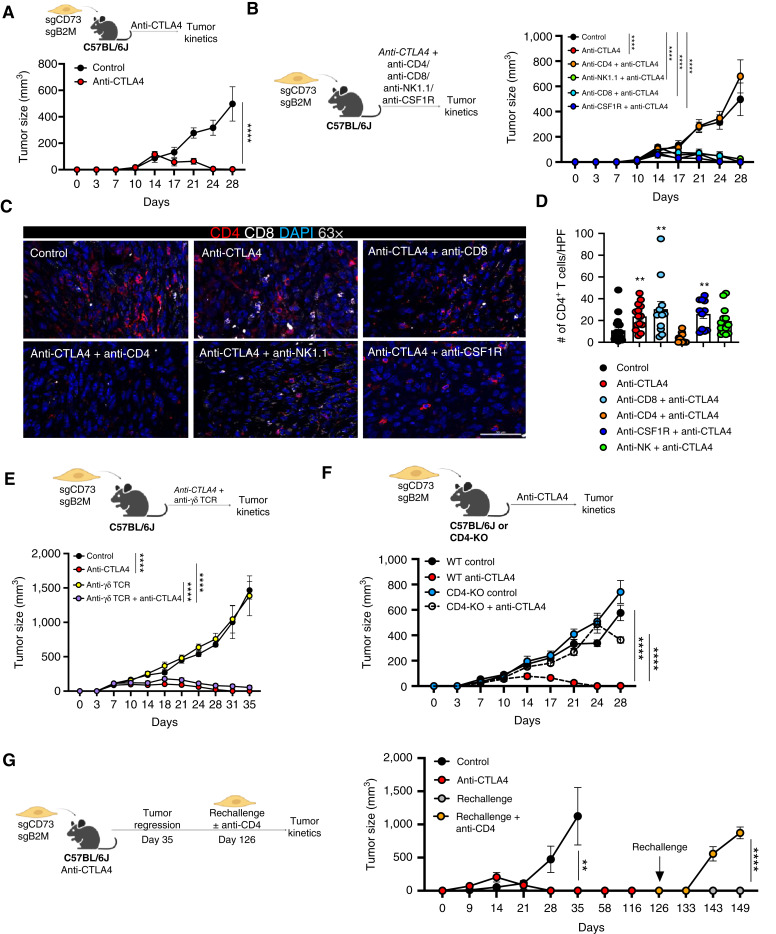
CD4^+^ T cells sensitize qM tumors lacking CD73 and MHC-I to anti-CTLA4 ICB. **A,** Schema and tumor kinetics for CD73 and B2M DKO tumor-bearing mice treated with the indicated antibodies. Data represent three independent experiments in which *n* = 3–7 for each group. **B,** Schema and tumor kinetics for CD73 and B2M DKO tumor-bearing mice treated with the indicated antibodies. Data represent three independent experiments in which *n* = 3–7 for each group. **C** and **D,** Representative immunofluorescence images of primary tumor sections obtained from (**B**) stained for CD4 (red), CD8 (white), and DAPI (blue) at 63× magnification. **D,** Bar graph represents quantification of CD4^+^ T cells in each high-power field (HPF) at 63× magnification for the indicated treatment groups. 3–5 fields of view from the tumor interior were obtained for each tumor. Data represent three independent experiments, with *n* = 3–5 mice in each group. **E,** Schema and tumor kinetics for CD73 and B2M DKO tumor-bearing mice treated with the indicated antibodies. Data represent two independent experiments in which *n* = 3–9 for each group. **F,** Schema and tumor kinetics for CD73 and B2M DKO tumors propagated in WT or CD4-KO mice treated with the indicated antibodies. Data represent three independent experiments in which *n* = 3–7 for each group. **G,** Schema and tumor kinetics for CD73 and B2M DKO tumor-bearing mice treated with the indicated antibodies. Responders were rechallenged with the same cell line as indicated with or without treatment with anti-CD4. Data represent three independent experiments in which *n* = 3–7 for each group. **A**, **B**, and **E–G,** Data represent the SEM, two-way ANOVA, **, *P* < 0.01; ****, *P* < 0.0001. **D,** Data represent the SEM, two-tailed unpaired *t* test, **, *P* < 0.01. Scale bars are 100 μm.

To functionally validate the relevance of these other immune cells in driving sensitization of sgCD73 tumors to anti-CTLA4 ICB, we abrogated the expression of MHC-I from sgCD73 cancer cells (via CRISPR/Cas9-mediated deletion of B2M, which is required for the stable cell-surface expression of MHC-I; Supplementary Fig. S6A). Whereas control sgCD73 cancer cells upregulated MHC-I and B2M in response to IFNγ treatment *in vitro*, DKO cells which lacked both CD73 and B2M failed to do so (Supplementary Fig. S6B and S6C). Accordingly, such a strategy would render DKO cells unresponsive to elimination by CD8^+^ T cells while retaining their susceptibility to macrophages, NK cells, and CD4^+^ T cells. These DKO cells were implanted orthotopically into syngeneic immunocompetent hosts and treated with control antibodies or anti-CTLA4 ICB. Strikingly, tumor-bearing mice that contained DKO cells which lacked both CD73 and B2M were completely sensitized to anti-CTLA4 ICB (Fig. 4A; Supplementary Fig. S6D).

To determine the functional importance of CD8^+^ T cells, NK cells, or myeloid cells in driving this sensitization, we depleted each of these cells using subset-specific antibodies which resulted in efficient depletion of each subset (Supplementary Fig. S6E–S6G). Antibody-based depletion of each of these immune cells failed to reverse sensitization (Fig. 4B; Supplementary Fig. S6H). Moreover, responding tumor-bearing mice recruited elevated numbers of CD4^+^ T cells to their tumors in response to anti-CTLA4 therapy even in the absence of CD8^+^ T cells, NK-cells, and macrophages (Fig. 4C and D). Similarly, antibody-based depletion of γδ T cells that also operate independently of MHC-I failed to reverse sensitization (Fig. 4E; Supplementary Fig. S6I and S6J). In sharp contrast, antibody mediated depletion of CD4^+^ T cells completely reversed the response of DKO tumor-bearing mice to anti-CTLA4 ICB (Fig. 4B; Supplementary Fig. S6H). Similarly, DKO cancer cells failed to respond to anti-CTLA4 ICB when implanted orthotopically in CD4-KO mice relative to WT mice (Fig. 4F; Supplementary Fig. S6K) once again, underscoring the functional importance of CD4^+^ T cells in sensitizing qM breast tumors lacking CD73 to anti-CTLA4 ICB.

To determine the efficacy of the aforementioned complete responses, DKO tumor-bearing mice that had responded to anti-CTLA4 ICB were rechallenged with the same tumor cells. Strikingly, these tumor-bearing mice remained tumor-free upon rechallenge, indicating the generation of antitumor memory (Fig. 4G). More importantly, these protective effects were lost when CD4^+^ T cells were depleted prior to rechallenge. Thus, CD4^+^ T cells not only drive the sensitization of qM tumors lacking CD73 to ICB but are also required for long-term memory responses (Fig. 4G).

### EMP is associated with CD73 expression in other murine models and human breast cancers

Our preclinical murine models of epithelial and qM tumors have enabled us to identify the EMP program and cancer cell–intrinsic expression of CD73 as important determinants of responsiveness to anti-CTLA4 ICB therapy. To determine the generalizability of our findings, we first assessed whether other murine models of triple-negative breast cancer (TNBC) that are also resistant to ICB therapy demonstrated similar associations. To this end, we focused our analyses on a syngeneic transplant tumor model established from the KPB25Luv cell line bearing mutations in *Trp53* and *Brca1*, resembling basal-like breast cancers and described by Hollern and colleagues (21).

**Figure 5.**
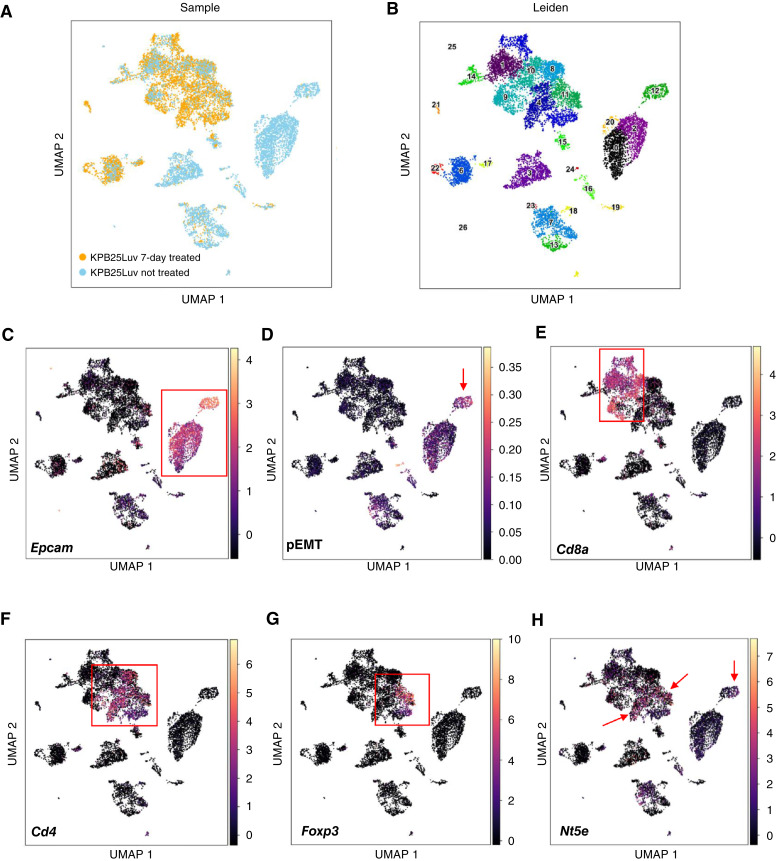
CD73 expression correlates with EMP in other murine models. scRNA-seq UMAP visualizations of untreated and 7-day immunotherapy-treated breast cancer samples from Hollern and colleagues. **A,** UMAP projection showing cell distribution colored by sample condition (treated vs. untreated). **B,** UMAP projection colored by Leiden cluster assignments. See also Supplementary Table S2. **C–H,** Feature plots displaying the normalized expression or signature scores across the UMAP space for (**C**) *Epcam*, (**D**) pEMT signature, (**E**) *Cd8a*, (**F**) *Cd4*, (**G**) *Foxp3*, and (**H**) *Nt5e*.

To characterize the transcriptional shifts within the breast cancer TME following immunotherapy, we analyzed scRNA-seq data sets on untreated samples and those treated with ICB inhibitors for 7 days. Following QC and Harmony-based integration, UMAP visualization demonstrated a well-mixed distribution of treated and untreated cells across most clusters, indicating successful batch mitigation while preserving biological heterogeneity (Fig. 5A). Leiden clustering resolved the TME into diverse compartments, including epithelial/tumor cells, stroma, and a robust immune infiltrate (Fig. 5B; Supplementary Table S2). The epithelial tumor cell compartment was distinctly identified by the ubiquitous expression of the canonical marker *Epcam* (Fig. 5C). Notably, pathway scoring revealed significant intratumoral heterogeneity. Within the broader *Epcam* + tumor cell population, a distinct cluster emerged that highly expressed a pEMT signature (Fig. 5D).

We next evaluated the composition of the T-cell infiltrate and the expression of the immunomodulatory enzyme *Nt5e* (CD73). The T-cell compartment clustered into distinct phenotypic states, prominently featuring cytotoxic CD8A^+^ T cells, CD4^+^ helper T cells, and a concentrated population of Tregs identified by high *Foxp3* expression within the CD4^+^ T-cell subset (Fig. 5E–G). Mapping *Nt5e* expression across the UMAP space revealed a highly specific and targeted distribution (Fig. 5H). Within the tumor compartment, *Nt5e* was not expressed uniformly by all *Epcam* + cells; rather, it was exclusively upregulated by the specific tumor subpopulation exhibiting the pEMT signature. More strikingly, the highest overall abundance of *Nt5e* expression was localized within the immune compartment. It was predominantly expressed by the CD4^+^ T-cell populations, with the densest expression mapping directly to the *Foxp3*^+^ Treg clusters. Together, these data indicate that both the partial EMT state of the breast cancer cells and the regulatory CD4^+^ T-cell populations converge on the expression of *Nt5e*, highlighting a potentially shared axis of immunosuppression in the TME. These observations once again underscore the importance of CD73, CD4^+^ T cells, and pEMT as being associated with resistance to immunotherapy in a different basal-like breast cancer model.

**Figure 6.**
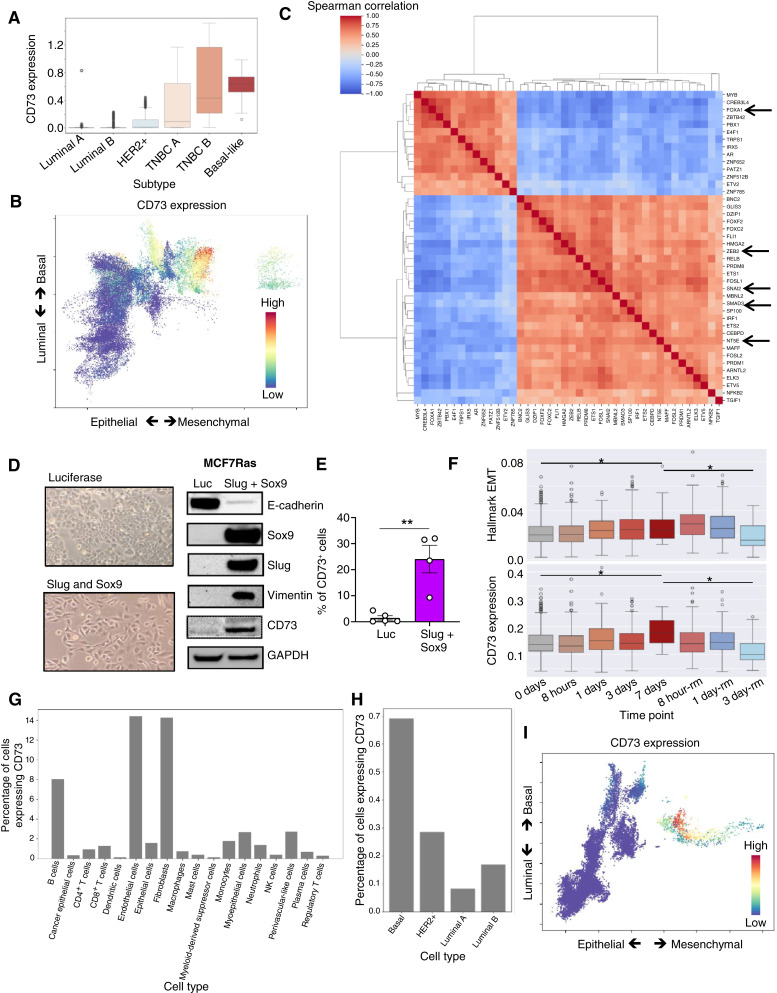
EMP regulates CD73 expression in human breast cancer. **A,** Box plot showing the expression level of CD73 in breast cancer cell lines belonging to different breast cancer subtypes. **B,** Scatter plot showing the relative position of single cells from different breast cancer cell lines on a 2D luminal–basal and epithelial–mesenchymal plane. Individual cells are colored by CD73 expression levels, with red denoting a higher expression. **C,** A clustered heatmap showing top TFs that are correlated with CD73 expression in the CCLE breast cancer cell lines. **D** and **E,** MCF7RAS cells expressing doxycycline-controlled control Luciferase or SLUG and SOX9 coexpressing constructs were treated with doxycycline for 4 days to induce EMP. **D,** Phase-contrast images and Western blots for the indicated markers. Data represent four independent experiments. **E,** Bar graph representing percentage of cells expressing CD73. Data represent the SEM, two-tailed unpaired *t* test, **, *P* < 0.01. **F,** Boxplot showing Hallmark EMT AUCell score and CD73 expression of MCF7 cells treated with TGFß followed by the removal of the same over a total period of 10 days. Student *t* test was performed to assess the significance of difference of means in expression between day 0 with day 7 and day 7 with day 10 (third day of TGFß removal) *, *P *< 0.05. **G,** Barplot showing the percentage of cells in which nonzero counts of reads were detected from scRNA-seq of human breast cancer–derived cells from different breast cancer subtypes. **H,** Barplot showing the percentage of cells with nonzero counts of reads that were detected in tumor cells belonging to different breast cancer subtypes from human breast cancer samples. **I,** Scatterplot showing the relative positions of tumor cells from human breast cancer samples on a 2D luminal–basal and epithelial–mesenchymal plane. Individual cells are colored by CD73 expression levels with red denoting a higher expression.

To assess the translational potential of our findings, we first asked whether and which types of human breast cancers express CD73. To that end, we analyzed publicly available bulk and scRNA-seq datasets of different breast cancer cell lines (28). We observed that CD73 was expressed in a subtype-specific manner with the highest levels of CD73 being expressed by TNBCs (Fig. 6A; Supplementary Fig. S7A). In sharp contrast, cell lines belonging to the luminal A/B subtypes expressed little to no CD73 (Fig. 6A; Supplementary Fig. S7A). Most strikingly, analysis of RNA-seq transcriptomic datasets of breast cancer cell lines from the CCLE revealed that CD73 expression was correlated strongly with EMP as well as partial EMP pathways (Supplementary Fig. S7B–S7D; ref. 25). To further assess whether CD73 expression by more-mesenchymal cancer cells is specifically associated with luminal or basal characteristics, we projected transcriptomics data obtained from scRNA-seq of breast cancer cell lines on a 2D epithelial–mesenchymal and luminal–basal plot (28). This analysis revealed that only cells that were enriched for a basal signature and were intermediate or high in mesenchymal signatures were more likely to express *NT5E* (the gene name for CD73) compared with luminal cells which were largely epithelial in nature (Fig. 6B). Taken together, these analyses suggest a strong correlation between CD73 expression and TNBCs that reside in a more-mesenchymal, basal-like state.

To understand the contribution of causal factors that could control cancer cell–intrinsic CD73 expression, we performed a correlation analysis of CD73 expression with all TFs using transcriptomic data of breast cancer cell lines from the CCLE. We observed that TFs expressed by luminal breast cancer cell lines, specifically *PBX2* and *FOXA1*, were negatively correlated with *NT5E* expression. In sharp contrast, TFs that are known to activate EMP, specifically *SNAI2* (SLUG), *ZEB2*, *SMAD3*, and *FOXC2*, were positively correlated with *NT5E* expression (Fig. 6C). To experimentally assess a causal connection between EMP and CD73 expression, we activated this program in MCF7RAS human breast cancer cells by doxycycline-controlled expression of two different EMT-inducing TFs SLUG and SOX9 (20, 29). Coexpression of these two EMT TFs led to a robust activation of EMP, as observed by the adoption of a more-mesenchymal morphology, loss of E-cadherin, and gain of vimentin relative to cells expressing a doxycycline-controlled luciferase construct. Most importantly, the acquisition of more-mesenchymal features was also accompanied by a concomitant increase in CD73 expression indicating a causal connection between EMP and CD73 (Fig. 6D and E; Supplementary Fig. S7E). To determine whether other EMT TFs could also regulate the expression of CD73, we induced the expression of either ZEB1, TWIST, or SLUG in MCF7RAS cells. The expression of these EMT TFs resulted in only a partial EMT as the cells retained varying levels of E-cadherin (Supplementary Fig. S7F). However, residence in this partial state also resulted in increased CD73 expression albeit to a lesser extent compared with cells that coexpressed SLUG and SOX9 and underwent a more-complete transition (Supplementary Fig. S7G).

To further assess causality, we analyzed scRNA-seq data from the MCF7 cell line that was induced to undergo an EMP by treatment with TGFβ for 7 days followed by a subsequent reversal of EMP by withdrawing TGFβ for 3 days (30). We observed that both the Hallmark EMT signature as well as *NT5E* expression were significantly upregulated by day 7 of TGFβ treatment. Strikingly, the expression of both EMT pathways and *NT5E* expression was reversibly lost as the signal for EMP was removed over the course of the next 3 days (Fig. 6F). Taken together, these findings establish the fact that the induction of cancer cell–intrinsic CD73 expression is EMP-dependent and that TFs can causally induce the expression of CD73 in a reversible manner.

Given the known immunosuppressive function of CD73, we sought to delineate its expression on various cell types within the tumor micro-environment of human breast tumors. Upon investigating a recently curated scRNA-seq atlas of human breast tumors (31), we found that several immune cells expressed varying levels of CD73 with the highest expression being present on B cells, in which it is known to generate adenosine, induce immunosuppressive effects, and likely regulate class-switching (32, 33). Intriguingly, CD73 in the TME was also largely expressed by more-mesenchymal cells such as fibroblasts and endothelial cells, indicating a more general association of CD73 expression with the mesenchymal state even in noncancer cells (Fig. 6G). Most importantly, within the epithelial, cancer cell population, basal-like cancer cells had the highest expression of CD73, followed by HER2+ and luminal B cancer cells (Fig. 6H). Finally, we analyzed tumor cells from a subset of patients with breast cancer to determine which molecular phenotype of cancer cells express the highest levels of CD73 (34). Once again, we observed that patient-derived breast cancer cells that expressed a basal and a partial EMP (pEMP)/full EMP phenotype were more likely to express CD73 in comparison with either luminal-epithelial or basal-epithelial cells (Fig. 6I). This was further exemplified by the fact that the same cells that expressed CD73 also expressed mesenchymal markers such as ZEB1, SLUG, and fibronectin and were low in E-cadherin expression (Supplementary Fig. S8A). In conclusion, analysis of multiple transcriptomic data sets of human breast cancer cell lines or scRNA-seq data-sets obtained from patients with breast cancer demonstrate a strong relationship between EMP and CD73 expression.

## Discussion

EMP has long been studied as a process that potentiates metastasis and drives resistance of breast tumors to multiple forms of therapies, including targeted therapies, chemotherapies, and, more recently, immunotherapies (6, 14, 35, 36). Our previous and current findings have underscored the importance of the adenosine-generating ectoenzyme, CD73, in driving such resistance of qM breast tumors to anti-CTLA4 ICB therapy (20). More specifically, inhibiting the expression of cancer cell–intrinsic CD73 can completely sensitize qM breast tumors to anti-CTLA4 ICB (20). Although our previous work uncovered the importance of targeting the cancer cell–intrinsic adenosinergic pathway for eliminating more-mesenchymal cancer cells, the precise identity of immune cells that enabled such eradication remained unknown. In this study, we present findings that identify the functional importance of CD4^+^ T cells in eliminating qM breast tumors lacking CD73 in response to anti-CTLA4 ICB.

We observed that the susceptibility of qM tumors lacking CD73 to anti-CTLA4 ICB was only partially dependent on CD8^+^ T cells. Given the ability of more-mesenchymal cancer cells to downregulate MHC-I expression as a consequence of activating the EMT program, this finding is perhaps not that surprising (18, 37). Moreover, in previous studies we have determined that CD8^+^ T cells infiltrating sgCD73 tumors do indeed exhibit greater cytolytic function relative to those present in control qM tumors even prior to administering ICB (20). However, despite retaining effector function, CD8^+^ T cells were dispensable for eliminating sgCD73 tumors in response to anti-CTLA4 ICB. This is likely because CD8^+^ T cells are outnumbered by their CD4^+^ T-cell counterparts in responding tumors as described in this study. Precisely why sgCD73 tumors recruit elevated numbers of CD4^+^ T cells but not CD8^+^ T cells in response to ICB treatment remains to be determined. Whether perturbation of CD73 in qM tumors promotes the release of cytokines and chemoattractants that are specific for CD4^+^ T cells is one possibility. Alternatively, anti-CTLA4 ICB treatment of sgCD73 tumors could result in more efficient priming and recruitment of peripheral CD4^+^ T cells relative to CD8^+^ T cells.

Whereas CD4^+^ T cells in the TME have primarily been studied as immunosuppressive Tregs, their antitumor functions in eliminating cancer cells, ostensibly akin to their CD8^+^ T-cell counterparts, is only beginning to emerge (27, 38). We observed marginally elevated numbers of T_H_1-like cells in sgCD73 tumors in response to anti-CTLA4 ICB treatment relative to those present in nonresponding qM control tumors and no changes in other helper T-cell subsets. Previous studies have shown that both anti-CTLA4 treatment and perturbation of CD73 signaling can specifically deplete Tregs and increase T_H_1-like cells (39, 40). The precise mechanism utilized by these CD4^+^ T cells in eliminating more-mesenchymal breast cancer cells remains to be established. It is plausible that IFNγ derived from these T_H_1-like cells could exert antitumor effects ultimately leading to eradication of qM tumors.

We demonstrated that sgCD73 cancer cells lacking MHC-I were also sensitized to anti-CTLA4 ICB in a CD4^+^ T cell–dependent manner, eliminating the contribution of NK cells or myeloid cells in tumor clearance. Moreover, CD4^+^ T cells were important not only for acute responses to anti-CTLA4 ICB but also for long-term memory responses, underscoring their biological importance in controlling more-mesenchymal breast tumors. A few studies have implicated the ability of CD4^+^ T cells to directly eliminate cancer cells that are MHC-II–proficient or –deficient by releasing proinflammatory cytokines (41–43). Other studies have identified alternative mechanisms in which CD4^+^ T cells enable tumoricidal myeloid cells to eliminate cancer cells regardless of MHC-II expression (44, 45). CD4^+^ T cells can also eliminate cancer cells by increasing the cytolytic functions of CD8^+^ T cells via dendritic cell licensing or via the formation of intratumoral triads (46–48). Determining precisely how CD4^+^ T cells eliminate qM tumors lacking CD73 in response to anti-CTLA4 ICB will be critical for gleaning mechanistic insights for the results presented in this study.

By performing transcriptomic analyses of various published data sets from other murine tumor models that resemble basal-like breast cancer as well as human breast tumors, we observed that the expression of cancer cell–intrinsic CD73 was associated specifically with more basal-like, TNBCs. The ability of various EMT TFs to induce the expression of CD73 also suggests a causal relationship between CD73 and the gain of more-mesenchymal properties by cancer cells. TNBCs activate components of the EMP program relative to other breast cancer subtypes and concomitantly resist ICB therapies (49–51). Given the ability of EMP to drive metastasis and resistance to therapies, our findings can spur translational efforts to (i) utilize the phenotypic plasticity of cancer cells along with CD73 expression and CD4^+^ T cells as predictive criteria for ICB responsiveness and (ii) therapeutically target these parameters in combination to potentiate the response of highly refractory TNBCs to anti-tumor immunity. Accordingly, such translational strategies hold the potential to be transformative for the treatment of human breast cancers.

We have previously demonstrated that qM tumors lacking CD73 are specifically sensitized to anti-CTLA4 but not anti–PD-1 ICB (20). The underlying reason(s) for this difference is unknown. Recognizing the importance of this observation, the goal of these studies was to first identify how disrupting CD73 signaling potentiates responses to anti-CTLA4 ICB and then apply the lessons learned to anti–PD-1 blockade in subsequent work. Several studies have outlined the ability of anti–PD-1 blockade to reinvigorate stem-like CD8^+^ T-cell progenitors (52, 53). However, given the higher numbers and biological relevance of CD4^+^ T cells but not CD8^+^ T cells in our models, this finding alone could explain the lack of response to anti–PD-1 blockade. Future studies are aimed at understanding whether (and which) differential mechanisms are utilized by sgCD73 tumors in response to anti-CTLA4 versus anti–PD-1 therapies. Although 15% to 30% of patients with breast cancer do respond to anti–PD-L1 blockade, a vast majority of them remain unresponsive to single-agent anti-CTLA4 or anti-CD73 ICB (5, 54, 55). Thus, the translational appeal of our work lies in determining mechanistically how combining both agents together (anti-CTLA4 and anti-CD73 ICB) generates synergistic responses to specifically eliminate qM tumors. Indeed, the adenosine antagonist ciforadenant synergizes with anti-CTLA4 ICB in preclinical mouse models and is currently being used in phase Ib/II clinical trials in combination with ipilimumab (anti-CTLA4) or nivolumab (anti–PD-1) for metastatic renal cancer (56). We propose that similar strategies can also be utilized for the treatment of refractory, more-mesenchymal TNBC tumors.

## Supplementary Material

Supplementary Figure S1 Supplementary Figure S1. Targeting CD73 sensitizes quasi-mesenchymal tumors to anti-CTLA4 immune checkpoint blockade therapy

Supplementary Figure S2 Supplementary Figure S2. Activation and exhaustion markers on CD8+ T-cell subsets present in responders and non-responders.

Supplementary Figure S3 Supplementary Figure S3. Targeting CD73 sensitizes quasi-mesenchymal tumors to anti-CTLA4 immune checkpoint blockade therapy in a CD4+ T-cell dependent manner.

Supplementary Figure S4 Supplementary Figure S4. Presence of CD4+ T-cell helper subsets in responders and non-responders.

Supplementary Figure S5 Supplementary Figure S5. Activation and exhaustion markers on CD4+ T-cell subsets present in responders and non-responders.

Supplementary Figure S6 Supplementary Figure S6. sgCD73 cells lacking MHC-I respond to anti-CTLA4 immune checkpoint blockade therapy in a CD4+ T-cell dependent manner.

Supplementary Figure S7 Supplementary Figure S7. EMP regulates CD73 expression on human breast cancer cell lines

Supplementary Figure S8 Supplementary Figure S8. CD73 expression on human breast cancer patient samples.

Supplementary Table 1 Supplementary Table 1. Genes present in each cluster depicted in Figure 1A

Supplementary Table 2 Supplementary Table 2. Genes present in each cluster depicted in Figure 5B

## Data Availability

The data generated in this study are available in the main article, supplemental files, or upon request to the corresponding author.
