# Supplementary material for: CD4+ T Cells Sensitize Quasimesenchymal Breast Tumors Lacking CD73 to Anti-CTLA4 Immune Checkpoint Blockade Therapy
Source: Cancer Res Commun. 2026 Jun 2;6(6):1278–94. doi: 10.1158/2767-9764.CRC-26-0304 (PMC13227059; doi:10.1158/2767-9764.CRC-26-0304)
Supplement: Supplementary Figure S1 — Targeting CD73 sensitizes quasi-mesenchymal tumors to anti-CTLA4 immune checkpoint blockade therapy [file crc-26-0304_supplementary_figure_s1_suppsf1.pptx]

## Slide 1
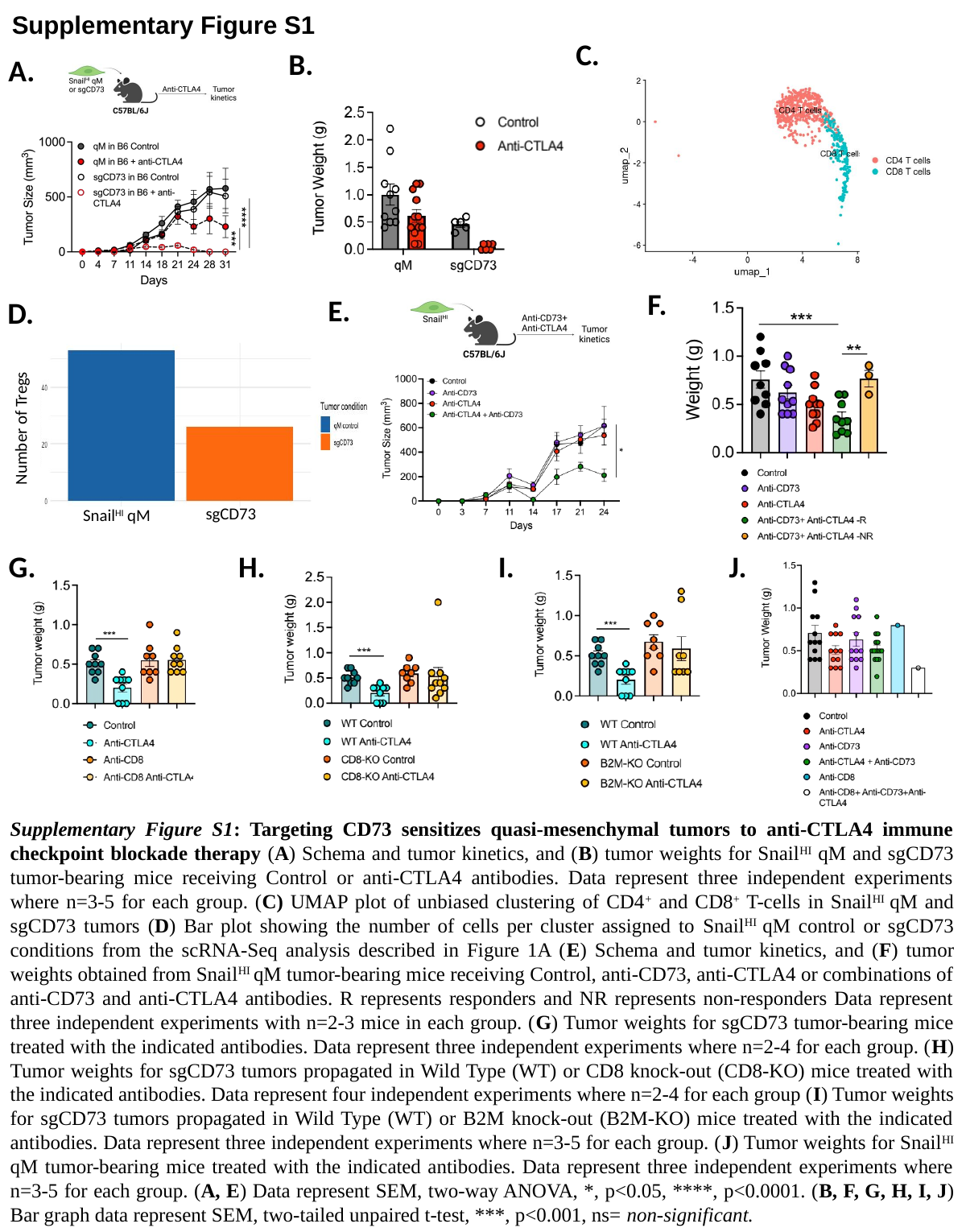

Supplementary Figure S1
C.
B.
A.
F.
E.
D.
Number of Tregs
sgCD73
SnailHI qM
G.
H.
I.
J.
Supplementary Figure S1: Targeting CD73 sensitizes quasi-mesenchymal tumors to anti-CTLA4 immune checkpoint blockade therapy (A) Schema and tumor kinetics, and (B) tumor weights for SnailHI qM and sgCD73 tumor-bearing mice receiving Control or anti-CTLA4 antibodies. Data represent three independent experiments where n=3-5 for each group. (C) UMAP plot of unbiased clustering of CD4+ and CD8+ T-cells in SnailHI qM and sgCD73 tumors (D) Bar plot showing the number of cells per cluster assigned to SnailHI qM control or sgCD73 conditions from the scRNA-Seq analysis described in Figure 1A (E) Schema and tumor kinetics, and (F) tumor weights obtained from SnailHI qM tumor-bearing mice receiving Control, anti-CD73, anti-CTLA4 or combinations of anti-CD73 and anti-CTLA4 antibodies. R represents responders and NR represents non-responders Data represent three independent experiments with n=2-3 mice in each group. (G) Tumor weights for sgCD73 tumor-bearing mice treated with the indicated antibodies. Data represent three independent experiments where n=2-4 for each group. (H) Tumor weights for sgCD73 tumors propagated in Wild Type (WT) or CD8 knock-out (CD8-KO) mice treated with the indicated antibodies. Data represent four independent experiments where n=2-4 for each group (I) Tumor weights for sgCD73 tumors propagated in Wild Type (WT) or B2M knock-out (B2M-KO) mice treated with the indicated antibodies. Data represent three independent experiments where n=3-5 for each group. (J) Tumor weights for SnailHI qM tumor-bearing mice treated with the indicated antibodies. Data represent three independent experiments where n=3-5 for each group. (A, E) Data represent SEM, two-way ANOVA, *, p<0.05, ****, p<0.0001. (B, F, G, H, I, J) Bar graph data represent SEM, two-tailed unpaired t-test, ***, p<0.001, ns= non-significant.
