# Supplementary material for: CD4+ T Cells Sensitize Quasimesenchymal Breast Tumors Lacking CD73 to Anti-CTLA4 Immune Checkpoint Blockade Therapy
Source: Cancer Res Commun. 2026 Jun 2;6(6):1278–94. doi: 10.1158/2767-9764.CRC-26-0304 (PMC13227059; doi:10.1158/2767-9764.CRC-26-0304)
Supplement: Supplementary Figure S2 — Activation and exhaustion markers on CD8+ T-cell subsets present in responders and non-responders. [file crc-26-0304_supplementary_figure_s2_suppsf2.pptx]

## Slide 1
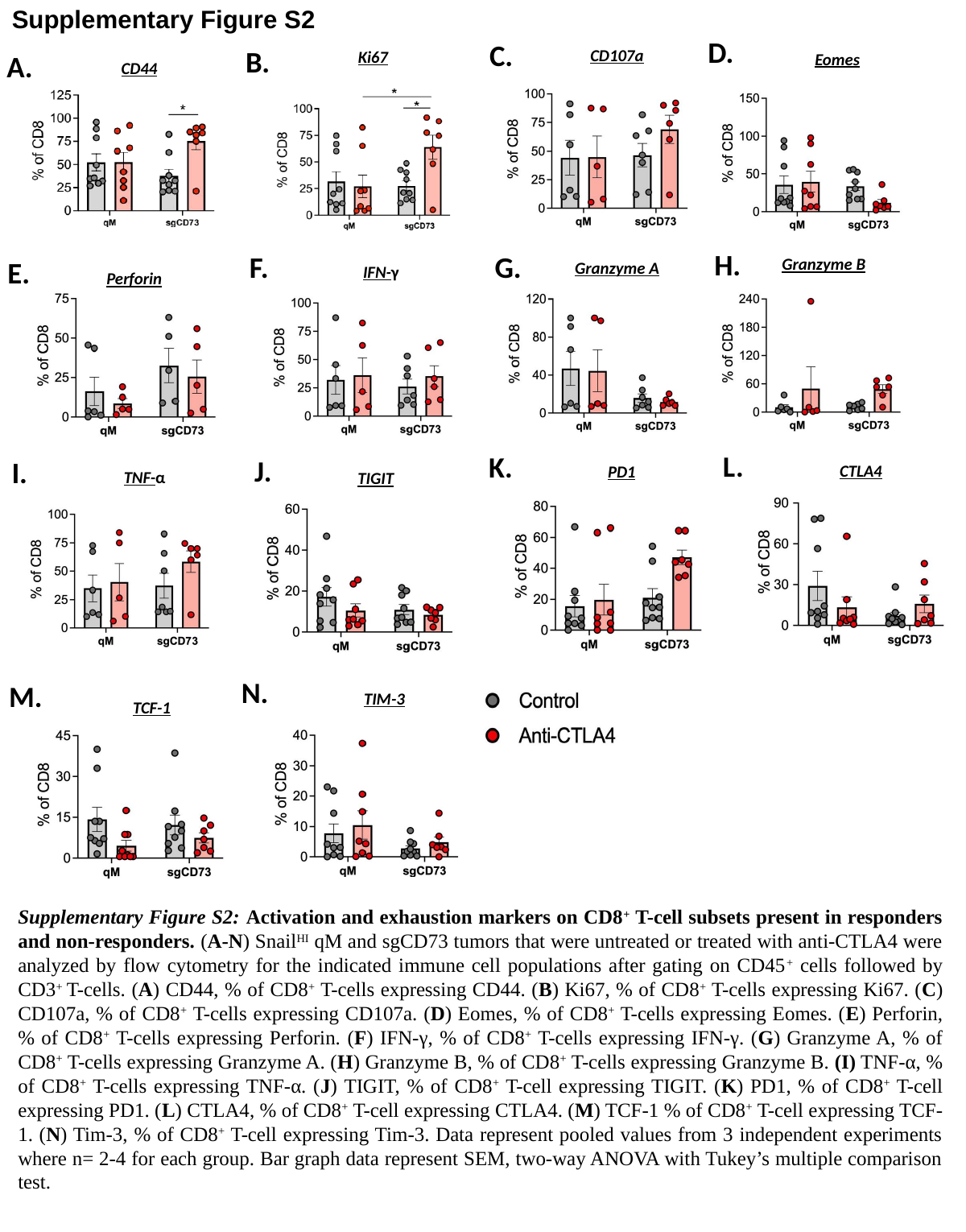

Supplementary Figure S2
D.
C.
B.
CD107a
Ki67
A.
Eomes
CD44
H.
F.
G.
Granzyme B
E.
Granzyme A
IFN-γ
Perforin
L.
K.
J.
I.
CTLA4
PD1
TNF-α
TIGIT
N.
M.
TIM-3
TCF-1
Supplementary Figure S2: Activation and exhaustion markers on CD8+ T-cell subsets present in responders and non-responders. (A-N) SnailHI qM and sgCD73 tumors that were untreated or treated with anti-CTLA4 were analyzed by flow cytometry for the indicated immune cell populations after gating on CD45+ cells followed by CD3+ T-cells. (A) CD44, % of CD8+ T-cells expressing CD44. (B) Ki67, % of CD8+ T-cells expressing Ki67. (C) CD107a, % of CD8+ T-cells expressing CD107a. (D) Eomes, % of CD8+ T-cells expressing Eomes. (E) Perforin, % of CD8+ T-cells expressing Perforin. (F) IFN-γ, % of CD8+ T-cells expressing IFN-γ. (G) Granzyme A, % of CD8+ T-cells expressing Granzyme A. (H) Granzyme B, % of CD8+ T-cells expressing Granzyme B. (I) TNF-α, % of CD8+ T-cells expressing TNF-α. (J) TIGIT, % of CD8+ T-cell expressing TIGIT. (K) PD1, % of CD8+ T-cell expressing PD1. (L) CTLA4, % of CD8+ T-cell expressing CTLA4. (M) TCF-1 % of CD8+ T-cell expressing TCF-1. (N) Tim-3, % of CD8+ T-cell expressing Tim-3. Data represent pooled values from 3 independent experiments where n= 2-4 for each group. Bar graph data represent SEM, two-way ANOVA with Tukey’s multiple comparison test.
