# Supplementary material for: CD4+ T Cells Sensitize Quasimesenchymal Breast Tumors Lacking CD73 to Anti-CTLA4 Immune Checkpoint Blockade Therapy
Source: Cancer Res Commun. 2026 Jun 2;6(6):1278–94. doi: 10.1158/2767-9764.CRC-26-0304 (PMC13227059; doi:10.1158/2767-9764.CRC-26-0304)
Supplement: Supplementary Figure S3 — Targeting CD73 sensitizes quasi-mesenchymal tumors to anti-CTLA4 immune checkpoint blockade therapy in a CD4+ T-cell dependent manner. [file crc-26-0304_supplementary_figure_s3_suppsf3.pptx]

## Slide 1
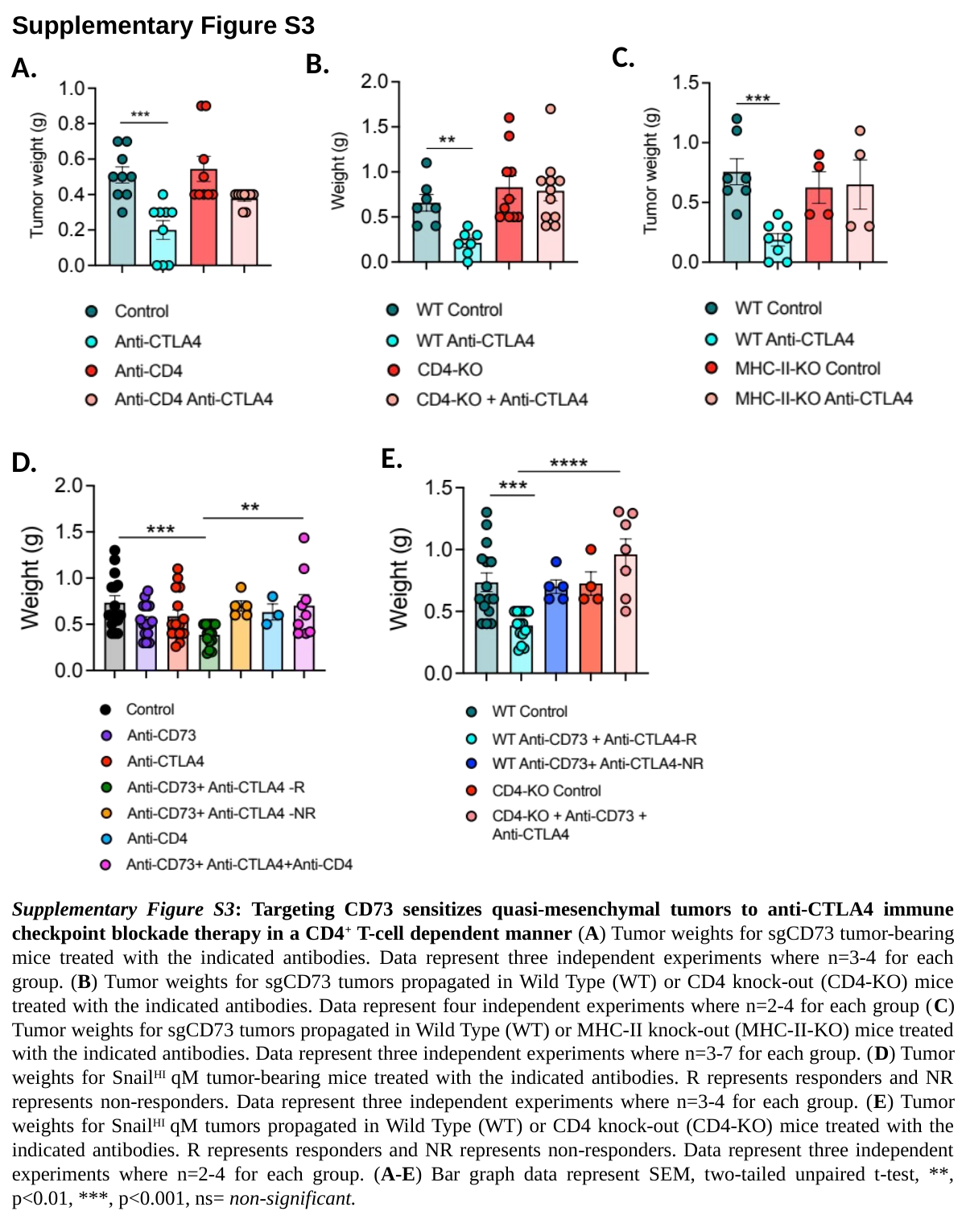

Supplementary Figure S3
C.
B.
A.
E.
D.
Supplementary Figure S3: Targeting CD73 sensitizes quasi-mesenchymal tumors to anti-CTLA4 immune checkpoint blockade therapy in a CD4+ T-cell dependent manner (A) Tumor weights for sgCD73 tumor-bearing mice treated with the indicated antibodies. Data represent three independent experiments where n=3-4 for each group. (B) Tumor weights for sgCD73 tumors propagated in Wild Type (WT) or CD4 knock-out (CD4-KO) mice treated with the indicated antibodies. Data represent four independent experiments where n=2-4 for each group (C) Tumor weights for sgCD73 tumors propagated in Wild Type (WT) or MHC-II knock-out (MHC-II-KO) mice treated with the indicated antibodies. Data represent three independent experiments where n=3-7 for each group. (D) Tumor weights for SnailHI qM tumor-bearing mice treated with the indicated antibodies. R represents responders and NR represents non-responders. Data represent three independent experiments where n=3-4 for each group. (E) Tumor weights for SnailHI qM tumors propagated in Wild Type (WT) or CD4 knock-out (CD4-KO) mice treated with the indicated antibodies. R represents responders and NR represents non-responders. Data represent three independent experiments where n=2-4 for each group. (A-E) Bar graph data represent SEM, two-tailed unpaired t-test, **, p<0.01, ***, p<0.001, ns= non-significant.
