# Supplementary material for: CD4+ T Cells Sensitize Quasimesenchymal Breast Tumors Lacking CD73 to Anti-CTLA4 Immune Checkpoint Blockade Therapy
Source: Cancer Res Commun. 2026 Jun 2;6(6):1278–94. doi: 10.1158/2767-9764.CRC-26-0304 (PMC13227059; doi:10.1158/2767-9764.CRC-26-0304)
Supplement: Supplementary Figure S4 — Presence of CD4+ T-cell helper subsets in responders and non-responders. [file crc-26-0304_supplementary_figure_s4_suppsf4.pptx]

## Slide 1
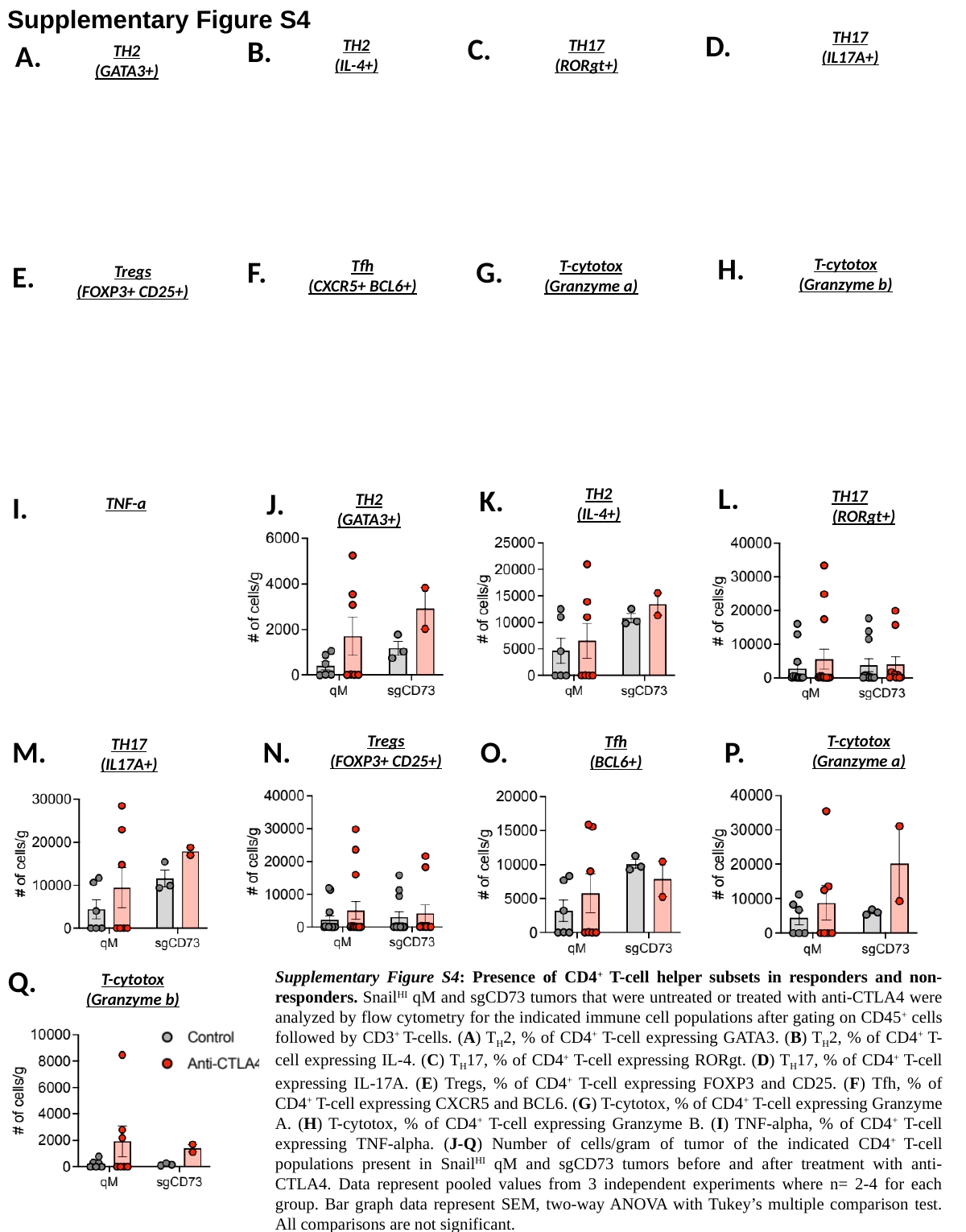

Supplementary Figure S4
D.
TH17
(IL17A+)
C.
B.
TH17
(RORgt+)
TH2
(IL-4+)
A.
TH2
(GATA3+)
H.
F.
G.
T-cytotox
(Granzyme b)
Tfh
(CXCR5+ BCL6+)
T-cytotox
(Granzyme a)
E.
Tregs
(FOXP3+ CD25+)
L.
K.
TH2
(IL-4+)
J.
TH17
(RORgt+)
I.
TH2
(GATA3+)
TNF-a
T-cytotox
(Granzyme a)
Tregs
(FOXP3+ CD25+)
Tfh
(BCL6+)
M.
N.
O.
P.
TH17
(IL17A+)
Q.
Supplementary Figure S4: Presence of CD4+ T-cell helper subsets in responders and non-responders. SnailHI qM and sgCD73 tumors that were untreated or treated with anti-CTLA4 were analyzed by flow cytometry for the indicated immune cell populations after gating on CD45+ cells followed by CD3+ T-cells. (A) TH2, % of CD4+ T-cell expressing GATA3. (B) TH2, % of CD4+ T-cell expressing IL-4. (C) TH17, % of CD4+ T-cell expressing RORgt. (D) TH17, % of CD4+ T-cell expressing IL-17A. (E) Tregs, % of CD4+ T-cell expressing FOXP3 and CD25. (F) Tfh, % of CD4+ T-cell expressing CXCR5 and BCL6. (G) T-cytotox, % of CD4+ T-cell expressing Granzyme A. (H) T-cytotox, % of CD4+ T-cell expressing Granzyme B. (I) TNF-alpha, % of CD4+ T-cell expressing TNF-alpha. (J-Q) Number of cells/gram of tumor of the indicated CD4+ T-cell populations present in SnailHI qM and sgCD73 tumors before and after treatment with anti-CTLA4. Data represent pooled values from 3 independent experiments where n= 2-4 for each group. Bar graph data represent SEM, two-way ANOVA with Tukey’s multiple comparison test. All comparisons are not significant.
T-cytotox
(Granzyme b)
