# Supplementary material for: CD4+ T Cells Sensitize Quasimesenchymal Breast Tumors Lacking CD73 to Anti-CTLA4 Immune Checkpoint Blockade Therapy
Source: Cancer Res Commun. 2026 Jun 2;6(6):1278–94. doi: 10.1158/2767-9764.CRC-26-0304 (PMC13227059; doi:10.1158/2767-9764.CRC-26-0304)
Supplement: Supplementary Figure S5 — Activation and exhaustion markers on CD4+ T-cell subsets present in responders and non-responders. [file crc-26-0304_supplementary_figure_s5_suppsf5.pptx]

## Slide 1
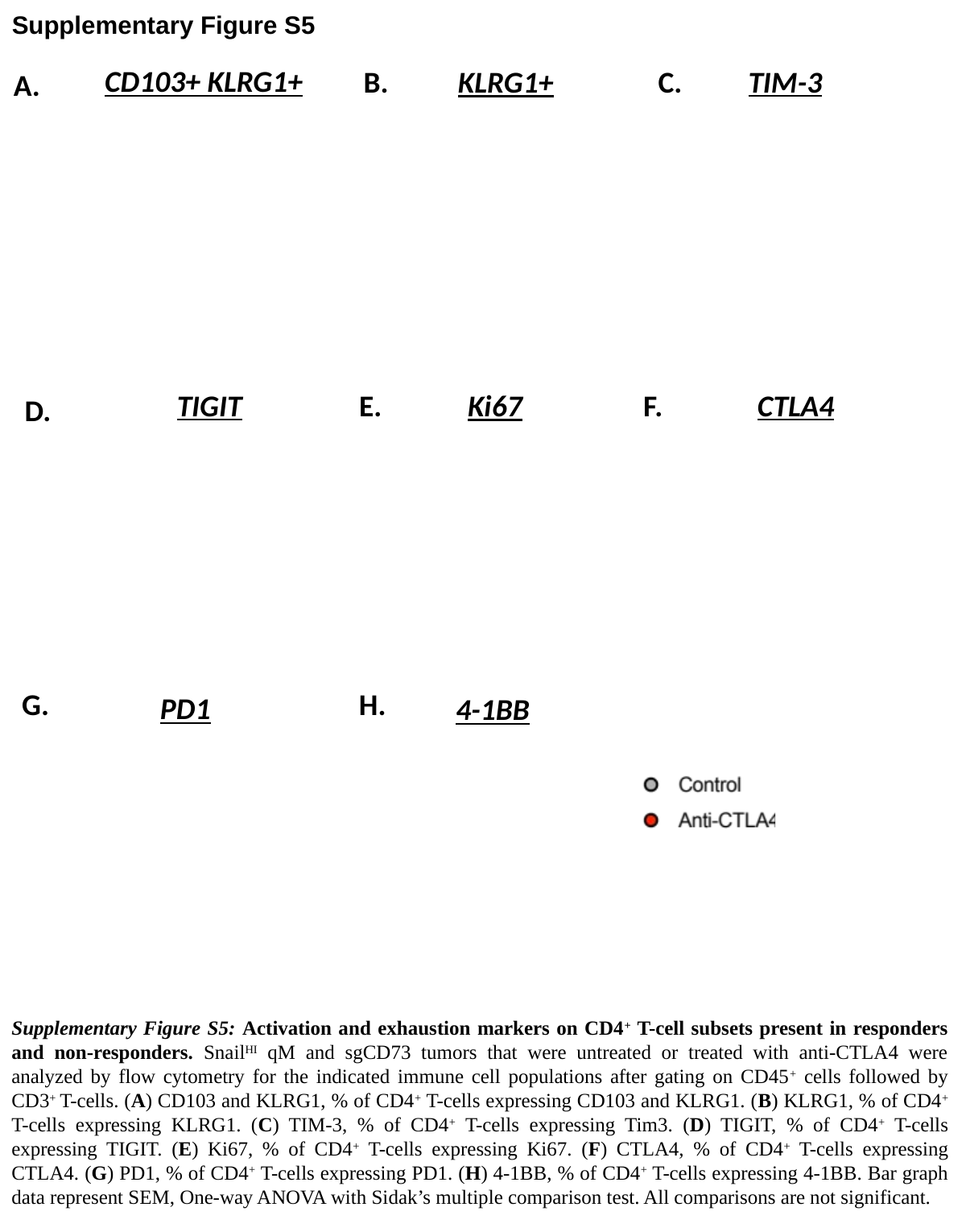

Supplementary Figure S5
CD103+ KLRG1+
TIM-3
KLRG1+
B.
C.
A.
TIGIT
CTLA4
Ki67
E.
F.
D.
H.
G.
PD1
4-1BB
Supplementary Figure S5: Activation and exhaustion markers on CD4+ T-cell subsets present in responders and non-responders. SnailHI qM and sgCD73 tumors that were untreated or treated with anti-CTLA4 were analyzed by flow cytometry for the indicated immune cell populations after gating on CD45+ cells followed by CD3+ T-cells. (A) CD103 and KLRG1, % of CD4+ T-cells expressing CD103 and KLRG1. (B) KLRG1, % of CD4+ T-cells expressing KLRG1. (C) TIM-3, % of CD4+ T-cells expressing Tim3. (D) TIGIT, % of CD4+ T-cells expressing TIGIT. (E) Ki67, % of CD4+ T-cells expressing Ki67. (F) CTLA4, % of CD4+ T-cells expressing CTLA4. (G) PD1, % of CD4+ T-cells expressing PD1. (H) 4-1BB, % of CD4+ T-cells expressing 4-1BB. Bar graph data represent SEM, One-way ANOVA with Sidak’s multiple comparison test. All comparisons are not significant.
