# Supplementary material for: CD4+ T Cells Sensitize Quasimesenchymal Breast Tumors Lacking CD73 to Anti-CTLA4 Immune Checkpoint Blockade Therapy
Source: Cancer Res Commun. 2026 Jun 2;6(6):1278–94. doi: 10.1158/2767-9764.CRC-26-0304 (PMC13227059; doi:10.1158/2767-9764.CRC-26-0304)
Supplement: Supplementary Figure S6 — sgCD73 cells lacking MHC-I respond to anti-CTLA4 immune checkpoint blockade therapy in a CD4+ T-cell dependent manner. [file crc-26-0304_supplementary_figure_s6_suppsf6.pptx]

## Slide 1
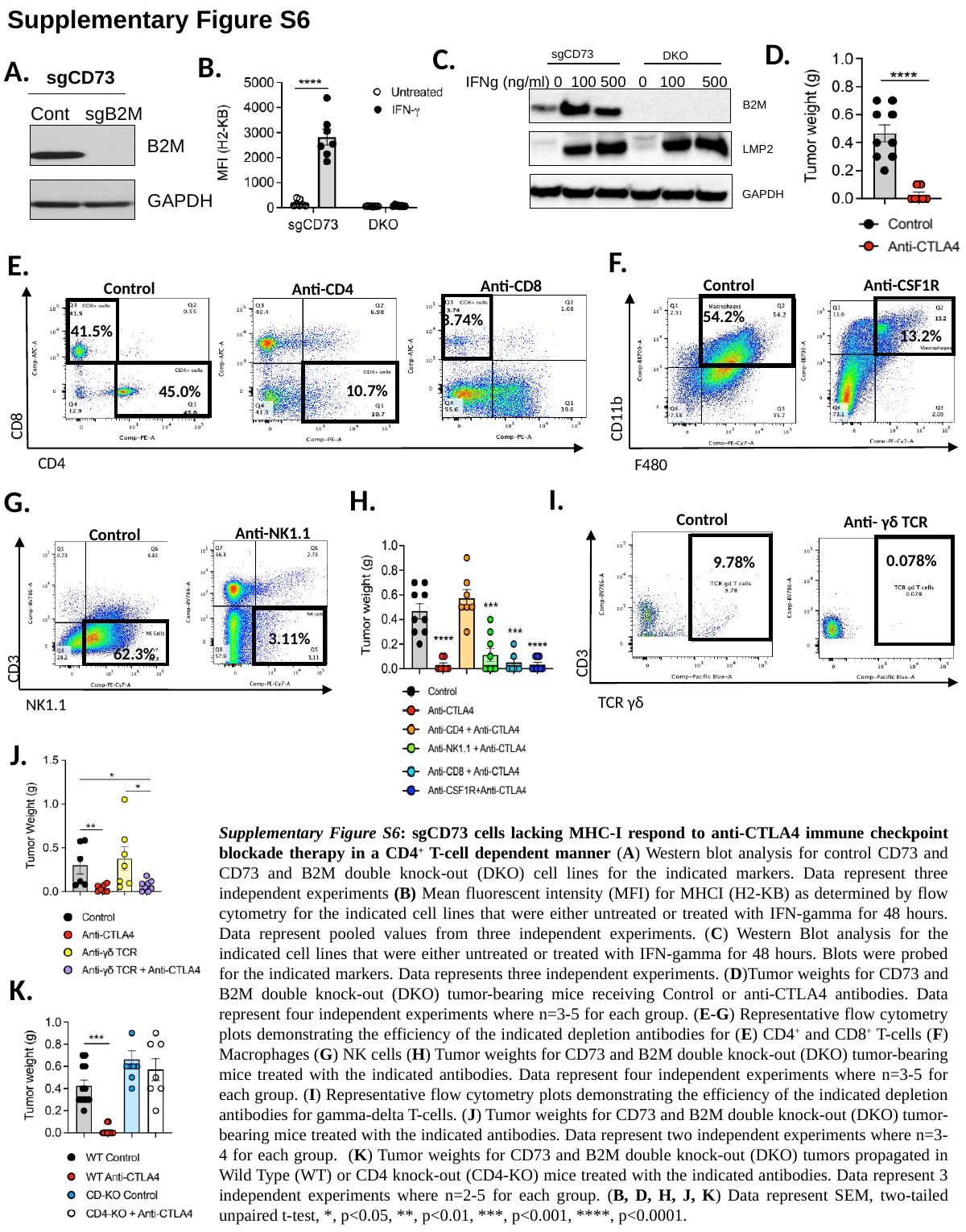

Supplementary Figure S6
D.
C.
sgCD73
DKO
 IFNg (ng/ml) 0 100 500 0 100 500
B2M
LMP2
GAPDH
B.
A.
sgCD73
Cont sgB2M
B2M
GAPDH
F.
E.
Control
Anti-CSF1R
54.2%
13.2%
CD11b
F480
Anti-CD8
Control
Anti-CD4
3.74%
41.5%
10.7%
45.0%
CD8
CD4
I.
H.
G.
Control
Anti- γδ TCR
0.078%
9.78%
CD3
 TCR γδ
Anti-NK1.1
Control
3.11%
62.3%
CD3
NK1.1
J.
Supplementary Figure S6: sgCD73 cells lacking MHC-I respond to anti-CTLA4 immune checkpoint blockade therapy in a CD4+ T-cell dependent manner (A) Western blot analysis for control CD73 and CD73 and B2M double knock-out (DKO) cell lines for the indicated markers. Data represent three independent experiments (B) Mean fluorescent intensity (MFI) for MHCI (H2-KB) as determined by flow cytometry for the indicated cell lines that were either untreated or treated with IFN-gamma for 48 hours. Data represent pooled values from three independent experiments. (C) Western Blot analysis for the indicated cell lines that were either untreated or treated with IFN-gamma for 48 hours. Blots were probed for the indicated markers. Data represents three independent experiments. (D)Tumor weights for CD73 and B2M double knock-out (DKO) tumor-bearing mice receiving Control or anti-CTLA4 antibodies. Data represent four independent experiments where n=3-5 for each group. (E-G) Representative flow cytometry plots demonstrating the efficiency of the indicated depletion antibodies for (E) CD4+ and CD8+ T-cells (F) Macrophages (G) NK cells (H) Tumor weights for CD73 and B2M double knock-out (DKO) tumor-bearing mice treated with the indicated antibodies. Data represent four independent experiments where n=3-5 for each group. (I) Representative flow cytometry plots demonstrating the efficiency of the indicated depletion antibodies for gamma-delta T-cells. (J) Tumor weights for CD73 and B2M double knock-out (DKO) tumor-bearing mice treated with the indicated antibodies. Data represent two independent experiments where n=3-4 for each group. (K) Tumor weights for CD73 and B2M double knock-out (DKO) tumors propagated in Wild Type (WT) or CD4 knock-out (CD4-KO) mice treated with the indicated antibodies. Data represent 3 independent experiments where n=2-5 for each group. (B, D, H, J, K) Data represent SEM, two-tailed unpaired t-test, *, p<0.05, **, p<0.01, ***, p<0.001, ****, p<0.0001.
K.
