# Supplementary material for: CD4+ T Cells Sensitize Quasimesenchymal Breast Tumors Lacking CD73 to Anti-CTLA4 Immune Checkpoint Blockade Therapy
Source: Cancer Res Commun. 2026 Jun 2;6(6):1278–94. doi: 10.1158/2767-9764.CRC-26-0304 (PMC13227059; doi:10.1158/2767-9764.CRC-26-0304)
Supplement: Supplementary Figure S7 — EMP regulates CD73 expression on human breast cancer cell lines [file crc-26-0304_supplementary_figure_s7_suppsf7.pptx]

## Slide 1
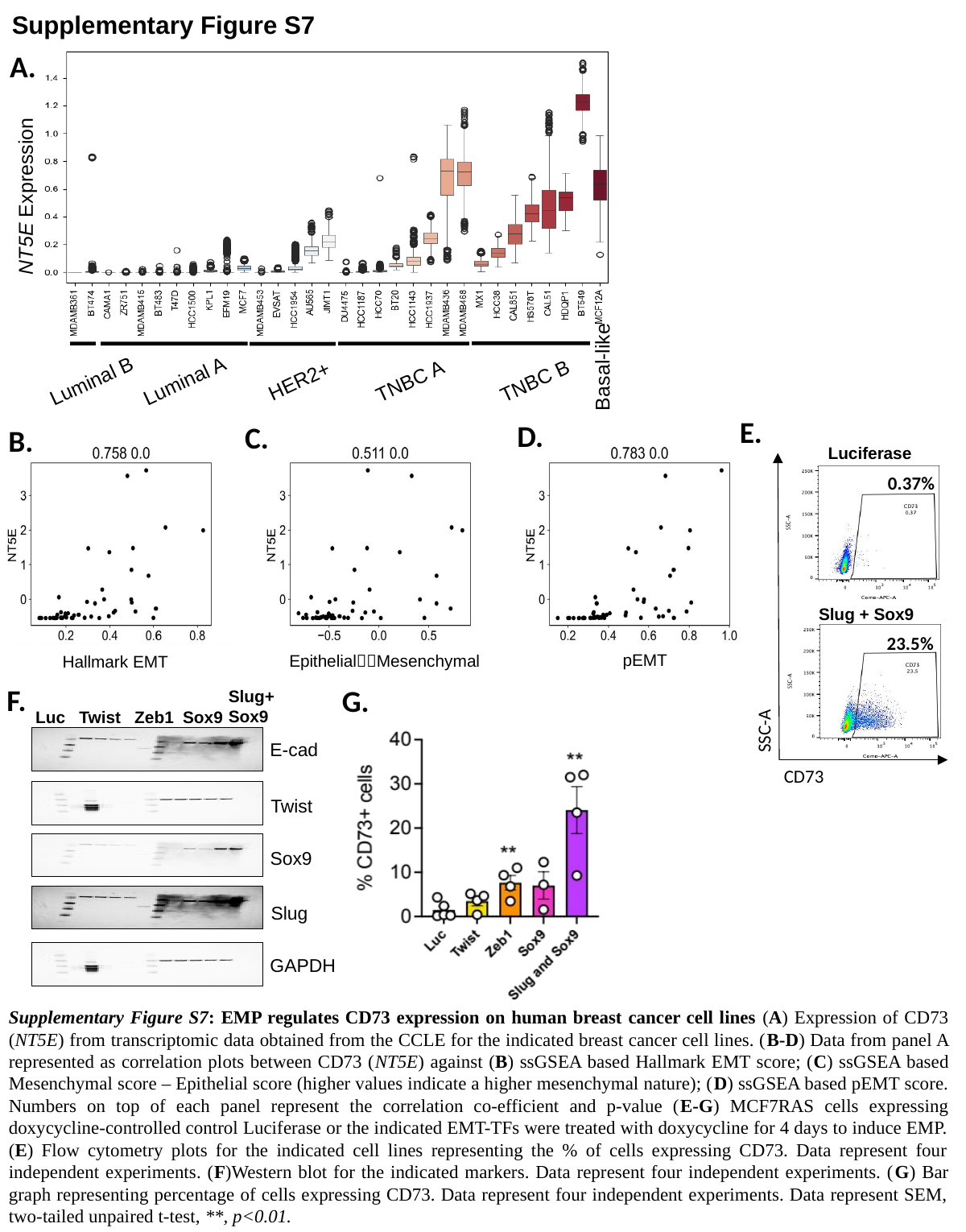

Supplementary Figure S7
A.
NT5E Expression
Basal-like
Luminal B
Luminal A
HER2+
TNBC A
TNBC B
E.
D.
C.
B.
Luciferase
0.37%
Slug + Sox9
23.5%
SSC-A
CD73
pEMT
EpithelialMesenchymal
Hallmark EMT
F.
G.
Slug+
Sox9
Luc Twist Zeb1 Sox9
E-cad
Twist
Sox9
Slug
GAPDH
Supplementary Figure S7: EMP regulates CD73 expression on human breast cancer cell lines (A) Expression of CD73 (NT5E) from transcriptomic data obtained from the CCLE for the indicated breast cancer cell lines. (B-D) Data from panel A represented as correlation plots between CD73 (NT5E) against (B) ssGSEA based Hallmark EMT score; (C) ssGSEA based Mesenchymal score – Epithelial score (higher values indicate a higher mesenchymal nature); (D) ssGSEA based pEMT score. Numbers on top of each panel represent the correlation co-efficient and p-value (E-G) MCF7RAS cells expressing doxycycline-controlled control Luciferase or the indicated EMT-TFs were treated with doxycycline for 4 days to induce EMP. (E) Flow cytometry plots for the indicated cell lines representing the % of cells expressing CD73. Data represent four independent experiments. (F)Western blot for the indicated markers. Data represent four independent experiments. (G) Bar graph representing percentage of cells expressing CD73. Data represent four independent experiments. Data represent SEM, two-tailed unpaired t-test, **, p<0.01.
