# Supplementary material for: CD4+ T Cells Sensitize Quasimesenchymal Breast Tumors Lacking CD73 to Anti-CTLA4 Immune Checkpoint Blockade Therapy
Source: Cancer Res Commun. 2026 Jun 2;6(6):1278–94. doi: 10.1158/2767-9764.CRC-26-0304 (PMC13227059; doi:10.1158/2767-9764.CRC-26-0304)
Supplement: Supplementary Figure S8 — CD73 expression on human breast cancer patient samples. [file crc-26-0304_supplementary_figure_s8_suppsf8.pptx]

## Slide 1
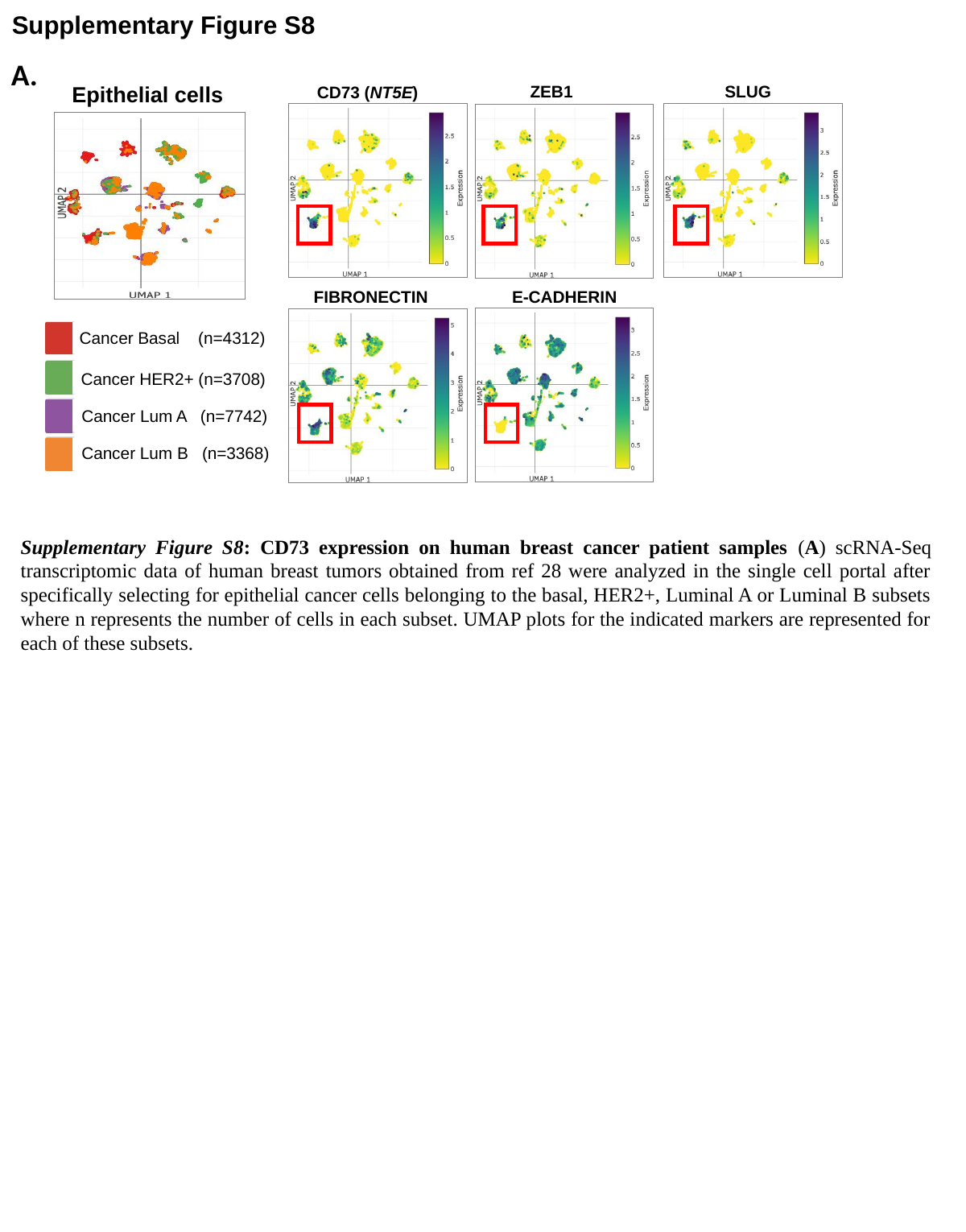

Supplementary Figure S8
A.
Epithelial cells
ZEB1
SLUG
CD73 (NT5E)
E-CADHERIN
FIBRONECTIN
Cancer Basal (n=4312)
Cancer HER2+ (n=3708)
Cancer Lum A (n=7742)
Cancer Lum B (n=3368)
Supplementary Figure S8: CD73 expression on human breast cancer patient samples (A) scRNA-Seq transcriptomic data of human breast tumors obtained from ref 28 were analyzed in the single cell portal after specifically selecting for epithelial cancer cells belonging to the basal, HER2+, Luminal A or Luminal B subsets where n represents the number of cells in each subset. UMAP plots for the indicated markers are represented for each of these subsets.
