# Supplementary material for: CD4+ T Cells Sensitize Quasimesenchymal Breast Tumors Lacking CD73 to Anti-CTLA4 Immune Checkpoint Blockade Therapy
Source: Cancer Res Commun. 2026 Jun 2;6(6):1278–94. doi: 10.1158/2767-9764.CRC-26-0304 (PMC13227059; doi:10.1158/2767-9764.CRC-26-0304)
Supplement: Supplementary Table 1 — Genes present in each cluster depicted in Figure 1A [file crc-26-0304_supplementary_table_1_suppst1.docx]

**Supplementary Table 1: Genes present in each cluster depicted in Figure 1A**

**Cluster 0 B cells**

cluster.markers <- FindMarkers(danx, ident.1 =0, min.pct = 0.25)

|++++++++++++++++++++++++++++++++++++++++++++++++++| 100% elapsed=10s

> head(cluster.markers, n = 100)

p_val avg_log2FC pct.1 pct.2 p_val_adj

Fcmr 0.000000e+00 2.6580313 0.693 0.017 0.000000e+00

Bank1 0.000000e+00 2.5322032 0.711 0.039 0.000000e+00

Pax5 0.000000e+00 2.4695290 0.657 0.017 0.000000e+00

Igkc 0.000000e+00 5.0722286 0.958 0.046 0.000000e+00

Cd79a 0.000000e+00 3.9834118 0.970 0.027 0.000000e+00

Ebf1 0.000000e+00 3.7401276 0.940 0.062 0.000000e+00

Cd79b 0.000000e+00 3.1219841 0.853 0.039 0.000000e+00

Ighd 0.000000e+00 3.4469762 0.886 0.029 0.000000e+00

Ighm 0.000000e+00 3.5589081 0.978 0.246 0.000000e+00

Mef2c 0.000000e+00 3.0843168 0.882 0.140 0.000000e+00

Ly6d 0.000000e+00 3.4892170 0.879 0.031 0.000000e+00

Iglc3 0.000000e+00 2.7172031 0.697 0.021 0.000000e+00

Iglc2 0.000000e+00 3.3441669 0.820 0.023 0.000000e+00

H2-Aa 0.000000e+00 2.3943922 0.996 0.257 0.000000e+00

Ms4a1 0.000000e+00 3.3863465 0.866 0.021 0.000000e+00

Cd74 6.130601e-298 2.3882968 1.000 0.325 1.032148e-293

H2-Eb1 4.302153e-291 2.0269734 0.982 0.230 7.243104e-287

H2-Ab1 1.315096e-290 2.0149622 0.993 0.244 2.214096e-286

Scd1 7.889363e-266 2.4952785 0.756 0.144 1.328253e-261

Cr2 9.414711e-262 2.2997673 0.558 0.015 1.585061e-257

H2-Ob 1.140625e-254 2.2469861 0.618 0.050 1.920356e-250

Gm31243 2.788041e-248 1.9843575 0.531 0.014 4.693945e-244

Bcl11a 2.267203e-246 2.1931573 0.585 0.034 3.817062e-242

Fcer2a 4.309203e-245 2.0960854 0.531 0.016 7.254974e-241

Ralgps2 5.365689e-238 2.3393789 0.684 0.116 9.033674e-234

H2-DMb2 1.856127e-227 1.9522744 0.584 0.050 3.124975e-223

Cd37 5.544814e-214 2.0348142 0.699 0.150 9.335250e-210

Pou2af1 1.033252e-201 1.7633509 0.476 0.023 1.739584e-197

Cd83 1.071778e-198 2.0675395 0.566 0.067 1.804445e-194

Mzb1 1.578859e-189 1.6812998 0.430 0.014 2.658168e-185

Cd22 8.979813e-189 1.7122754 0.455 0.024 1.511841e-184

Tnfrsf13c 9.003371e-189 1.5975205 0.427 0.013 1.515808e-184

Cd19 1.436013e-187 1.6457795 0.419 0.011 2.417672e-183

Pou2f2 3.560917e-180 1.9091689 0.601 0.112 5.995159e-176

H2-Oa 1.622251e-175 1.6599540 0.484 0.047 2.731221e-171

Stap1 6.085959e-174 1.9480454 0.591 0.115 1.024632e-169

Vpreb3 1.355048e-173 1.6746976 0.391 0.010 2.281358e-169

Jund 9.332222e-170 1.5934180 0.955 0.689 1.571173e-165

Fcrla 1.690489e-168 1.5611184 0.396 0.016 2.846107e-164

Hvcn1 1.546337e-164 1.8329939 0.528 0.085 2.603413e-160

Cxcr4 2.963927e-163 1.8861429 0.759 0.274 4.990067e-159

Ly6e 8.019408e-162 1.3900666 0.938 0.614 1.350147e-157

Stk17b 3.492480e-158 1.4702308 0.925 0.546 5.879940e-154

Spib 3.980694e-158 1.4809034 0.373 0.014 6.701897e-154

Cd55 2.577189e-153 1.8810896 0.547 0.110 4.338956e-149

Siglecg 3.620703e-152 1.4736013 0.374 0.019 6.095815e-148

Npc2 4.662864e-150 -1.9361674 0.223 0.761 7.850398e-146

Napsa 1.126790e-146 1.4915262 0.521 0.087 1.897063e-142

Fxyd5 6.010977e-145 -1.8131248 0.188 0.753 1.012008e-140

Blnk 1.035147e-144 1.4885018 0.408 0.038 1.742774e-140

Cd180 7.173099e-137 1.5735909 0.391 0.038 1.207663e-132

Rasgrp3 1.832035e-136 1.4037156 0.340 0.017 3.084414e-132

Ciita 5.631452e-133 1.5629577 0.438 0.065 9.481113e-129

Gm30211 2.844641e-131 1.6423697 0.306 0.009 4.789237e-127

Cxcr5 1.675567e-128 1.3363281 0.320 0.016 2.820984e-124

H3f3a 5.568426e-126 0.9650033 0.978 0.906 9.375002e-122

Cd69 8.601540e-126 1.6966716 0.668 0.226 1.448155e-121

Man1a 1.334656e-119 1.6749552 0.627 0.255 2.247027e-115

Fchsd2 7.969338e-119 1.8157353 0.537 0.163 1.341718e-114

B3gnt5 8.496375e-118 1.3038391 0.325 0.025 1.430450e-113

BE692007 1.165388e-117 1.6005070 0.554 0.156 1.962047e-113

H2-Eb2 2.136695e-114 1.1488385 0.280 0.011 3.597340e-110

Fcrl1 2.766658e-114 1.1756483 0.291 0.015 4.657946e-110

Arl4c 6.665471e-113 -1.4559402 0.021 0.502 1.122199e-108

Iglc1 8.274487e-109 1.9185369 0.268 0.011 1.393093e-104

Txn1 2.229944e-106 -1.8487135 0.270 0.705 3.754334e-102

Ifitm2 3.452274e-106 -2.3695058 0.041 0.501 5.812249e-102

Lgals1 5.974376e-106 -3.2276843 0.177 0.616 1.005846e-101

Serinc3 7.153651e-106 1.3038333 0.769 0.461 1.204389e-101

Cd63 1.372212e-105 -2.5107683 0.037 0.488 2.310257e-101

Ctsd 6.105241e-104 -1.7989219 0.089 0.562 1.027878e-99

Swap70 1.216466e-103 1.5437787 0.520 0.174 2.048042e-99

Klf2 7.504715e-102 1.2241695 0.891 0.572 1.263494e-97

Chst3 6.417026e-101 1.1645504 0.257 0.013 1.080370e-96

Ptprcap 3.378129e-98 1.3043564 0.601 0.227 5.687418e-94

Ikzf3 5.240927e-98 1.3473379 0.375 0.068 8.823624e-94

Tsc22d3 1.253568e-97 1.3772735 0.764 0.473 2.110508e-93

Anxa2 3.036627e-97 -2.1948150 0.076 0.507 5.112465e-93

Zfp36l1 5.288706e-97 1.3725961 0.837 0.611 8.904066e-93

Cd9 5.781223e-97 -2.4940169 0.045 0.475 9.733267e-93

Serpinb1a 2.648165e-96 1.3221427 0.325 0.044 4.458451e-92

Snn 3.344955e-95 1.3950567 0.383 0.081 5.631566e-91

Chchd10 1.055111e-94 1.3614879 0.399 0.085 1.776385e-90

Gm8369 1.811398e-94 1.4846161 0.605 0.227 3.049670e-90

Xist 2.107184e-94 1.2605884 0.820 0.467 3.547654e-90

Pkm 1.287406e-93 -1.5086559 0.293 0.714 2.167477e-89

Lgals3 1.205158e-92 -1.9768371 0.070 0.497 2.029003e-88

Syk 6.916932e-92 1.4220055 0.472 0.142 1.164535e-87

Irf8 1.463969e-91 1.2361998 0.537 0.188 2.464738e-87

Emb 3.130513e-91 -1.9380176 0.030 0.444 5.270531e-87

Zfp318 6.540463e-91 1.4819586 0.408 0.106 1.101152e-86

Serp1 5.940389e-90 1.2383622 0.755 0.502 1.000124e-85

Actn1 1.897084e-88 -1.3279241 0.024 0.430 3.193930e-84

Mt1 5.212335e-88 -2.3017351 0.054 0.466 8.775486e-84

Ldha 5.474895e-88 -1.4253451 0.243 0.668 9.217534e-84

Myl6 1.031787e-87 -1.1378593 0.643 0.895 1.737116e-83

Prdx5 1.770777e-87 -1.7539513 0.235 0.625 2.981279e-83

Ddx5 1.785117e-87 0.8057150 0.974 0.839 3.005423e-83

Tagln2 1.934577e-85 -1.3114655 0.126 0.559 3.257053e-81

Ltb 4.141011e-85 1.1123599 0.713 0.329 6.971805e-81

**Cluster 1 CD4 T-cells**

cluster.markers <- FindMarkers(danx, ident.1 =1, min.pct = 0.25)

|++++++++++++++++++++++++++++++++++++++++++++++++++| 100% elapsed=09s

> head(cluster.markers, n = 100)

p_val avg_log2FC pct.1 pct.2 p_val_adj

Trbc2 0.000000e+00 2.6444429 0.924 0.104 0.000000e+00

Lef1 1.294532e-299 2.5474070 0.850 0.094 2.179474e-295

Cd3d 4.480145e-283 2.2445770 0.894 0.105 7.542772e-279

Tcf7 1.582363e-270 2.4245808 0.873 0.125 2.664066e-266

Ms4a4b 6.322609e-266 2.2625798 0.901 0.122 1.064474e-261

Cd28 2.413297e-255 2.3656143 0.779 0.087 4.063026e-251

Bcl11b 6.188099e-246 2.1691403 0.756 0.084 1.041828e-241

Cd3g 1.303628e-208 1.7975086 0.703 0.086 2.194788e-204

Trac 1.011722e-186 1.7286718 0.645 0.085 1.703336e-182

Itk 3.063671e-178 1.6429087 0.608 0.075 5.157996e-174

Il7r 3.733255e-175 1.9592325 0.668 0.099 6.285309e-171

Txk 1.418447e-173 1.6613236 0.631 0.086 2.388097e-169

Cd3e 3.252179e-165 1.5132715 0.583 0.073 5.475369e-161

Lat 1.593794e-162 1.5386921 0.569 0.072 2.683311e-158

Prkcq 1.199339e-160 1.4915141 0.509 0.054 2.019206e-156

Satb1 5.395607e-154 1.8325920 0.912 0.305 9.084044e-150

Skap1 6.935818e-145 1.4681923 0.548 0.078 1.167714e-140

Emb 1.648035e-144 1.9415222 0.811 0.241 2.774631e-140

Ms4a6b 1.117958e-142 1.6116970 0.786 0.211 1.882195e-138

Trbc1 2.893739e-142 2.1223333 0.541 0.075 4.871899e-138

Rps16 9.468491e-140 0.9753005 0.993 0.982 1.594115e-135

S1pr1 3.354734e-139 1.5591401 0.763 0.188 5.648030e-135

Themis 4.435013e-132 1.2661273 0.454 0.053 7.466787e-128

Cd4 1.219231e-129 1.2462036 0.376 0.029 2.052697e-125

Rpl12 1.074183e-124 1.0285085 0.988 0.949 1.808495e-120

Cd247 6.136966e-119 1.1976564 0.422 0.051 1.033220e-114

Rps24 1.932219e-118 0.9249482 0.998 0.985 3.253084e-114

Rpl5 3.383351e-116 0.9644925 0.993 0.923 5.696210e-112

Nsg2 1.092044e-110 1.1038247 0.380 0.042 1.838565e-106

Fth1 2.018245e-110 -2.1760754 0.963 0.987 3.397917e-106

Thy1 9.447011e-109 1.3331189 0.512 0.096 1.590499e-104

Rps15a 1.973059e-108 0.8503165 0.993 0.962 3.321841e-104

Dapl1 8.053439e-108 1.5660589 0.392 0.046 1.355877e-103

Rpl18 6.978890e-107 0.8486208 0.995 0.946 1.174966e-102

Fam78a 9.811026e-106 1.1374142 0.426 0.063 1.651784e-101

Rplp1 7.807529e-105 0.7912974 1.000 0.987 1.314476e-100

Rps23 3.740046e-104 0.7982672 0.991 0.978 6.296741e-100

Tpt1 1.300509e-103 0.6820538 0.995 0.998 2.189538e-99

Trat1 1.053667e-102 0.9785748 0.276 0.017 1.773955e-98

Cd5 9.982318e-102 1.1090554 0.355 0.040 1.680623e-97

Rps14 3.581568e-100 0.7374091 0.998 0.980 6.029928e-96

Vps37b 5.904253e-100 1.5479053 0.696 0.247 9.940401e-96

Fyb 9.430668e-99 1.3434626 0.675 0.213 1.587747e-94

Rps13 4.226763e-98 0.7767361 0.995 0.972 7.116178e-94

Rpl13a 5.112227e-98 0.7715551 0.995 0.966 8.606945e-94

Lck 6.522176e-97 1.1934959 0.454 0.082 1.098074e-92

Rpl30 1.249740e-96 0.7294297 0.998 0.980 2.104062e-92

Saraf 5.730406e-94 1.5087012 0.661 0.260 9.647711e-90

Inpp4b 6.017432e-94 1.2992602 0.541 0.138 1.013095e-89

Ets1 2.040019e-92 1.3199719 0.908 0.493 3.434576e-88

Rps29 6.553019e-92 0.7316403 0.998 0.981 1.103266e-87

Rps6 1.461948e-91 0.8237027 0.988 0.942 2.461336e-87

Limd2 3.405256e-91 1.2930584 0.816 0.373 5.733089e-87

H2-Q7 1.795699e-90 1.1953381 0.795 0.312 3.023239e-86

Gimap3 2.330726e-90 1.2902570 0.599 0.164 3.924010e-86

Gm2682 3.137373e-89 0.9711247 0.339 0.043 5.282082e-85

Grap2 5.530135e-89 1.1552659 0.493 0.109 9.310535e-85

Rps15 9.574242e-87 0.7225800 0.995 0.957 1.611919e-82

Hcst 1.514282e-86 1.2308977 0.661 0.218 2.549446e-82

Rps7 1.205205e-85 0.7546204 0.995 0.959 2.029084e-81

Rps3 6.643433e-84 0.7436663 0.991 0.952 1.118488e-79

Dgka 2.899102e-83 1.2407311 0.514 0.138 4.880928e-79

Rps3a1 8.493875e-83 0.6361312 0.995 0.979 1.430029e-78

Gimap6 9.805481e-83 1.1838152 0.767 0.286 1.650851e-78

Shisa5 1.435637e-81 1.1044011 0.935 0.601 2.417038e-77

Atp1b3 3.139142e-81 1.3419885 0.705 0.335 5.285059e-77

H2-K1 3.834476e-81 0.9812863 0.977 0.725 6.455724e-77

Ablim1 3.343371e-80 1.1121990 0.795 0.320 5.628899e-76

Rplp2 1.945784e-79 0.7158542 0.993 0.951 3.275923e-75

Tmsb10 5.006154e-79 0.8764945 0.982 0.785 8.428360e-75

Rpl19 3.238566e-78 0.6228639 0.995 0.975 5.452449e-74

Cd27 2.061002e-75 1.0170413 0.364 0.066 3.469903e-71

Rps19 6.936412e-75 0.7081722 0.995 0.955 1.167814e-70

Peli1 4.251360e-74 1.2672217 0.680 0.291 7.157590e-70

Rpl8 5.758895e-74 0.6660058 0.995 0.963 9.695676e-70

Rpl35a 2.747114e-73 0.6200609 1.000 0.978 4.625040e-69

Spn 4.427479e-72 1.0142474 0.366 0.073 7.454103e-68

Smc4 6.145170e-71 1.4594632 0.650 0.281 1.034601e-66

Cd6 9.876243e-71 0.9736029 0.302 0.045 1.662764e-66

Rps28 1.014432e-70 0.6865755 0.995 0.948 1.707898e-66

Rps10 3.408906e-70 0.5906980 0.993 0.985 5.739235e-66

Rps21 4.723963e-69 0.6646083 0.993 0.963 7.953264e-65

Cd74 3.012076e-68 -4.0970834 0.152 0.577 5.071131e-64

Rps5 5.312140e-67 0.6635770 0.998 0.946 8.943518e-63

H2-Aa 4.739429e-66 -3.4912686 0.090 0.529 7.979303e-62

Rps8 3.469599e-65 0.6010080 0.998 0.970 5.841417e-61

Ltb 4.308477e-65 0.9302147 0.820 0.363 7.253752e-61

H2-Ab1 1.085355e-64 -3.2972965 0.083 0.519 1.827303e-60

Rps20 5.882025e-64 0.6172692 1.000 0.968 9.902978e-60

Rapgef6 1.139033e-63 1.1229813 0.700 0.335 1.917677e-59

Arhgap15 1.497680e-63 1.0437633 0.650 0.260 2.521495e-59

Rpl17 3.420248e-63 0.6291010 0.991 0.963 5.758330e-59

Ccr7 4.125385e-63 1.0333539 0.671 0.251 6.945498e-59

Ftl1 9.966953e-63 -1.8713496 0.719 0.878 1.678036e-58

Rps4x 2.324780e-62 0.5643944 0.998 0.947 3.914000e-58

Gimap4 3.997052e-62 0.9942609 0.576 0.192 6.729437e-58

Rps9 8.281657e-62 0.5006397 0.998 0.991 1.394300e-57

H2-Eb1 9.178572e-62 -3.0830867 0.081 0.503 1.545304e-57

Ptpn18 1.865629e-61 0.9402664 0.850 0.460 3.140973e-57

Rpsa 4.775146e-61 0.6174689 1.000 0.955 8.039436e-57

**Cluster2 cancer cells**

cluster.markers <- FindMarkers(danx, ident.1 =2, min.pct = 0.25)

|++++++++++++++++++++++++++++++++++++++++++++++++++| 100% elapsed=12s

> head(cluster.markers, n = 100)

p_val avg_log2FC pct.1 pct.2 p_val_adj

Gng11 6.592944e-203 2.6002052 0.835 0.148 1.109988e-198

Tnnt2 5.918788e-193 2.2475612 0.744 0.111 9.964872e-189

Krt8 2.802489e-171 2.1151632 0.733 0.120 4.718271e-167

Lgals1 9.280875e-171 2.7854764 0.972 0.423 1.562528e-166

S100a6 6.825891e-167 2.8033668 0.997 0.536 1.149207e-162

Krt18 6.132317e-164 1.8497726 0.713 0.114 1.032437e-159

S100a4 1.979708e-155 2.8324898 0.915 0.286 3.333037e-151

Hmga2 4.074082e-150 1.9841745 0.727 0.134 6.859124e-146

Vim 2.390897e-138 2.0296142 0.967 0.499 4.025315e-134

Cd52 3.938808e-137 -3.6979456 0.055 0.824 6.631377e-133

Tpm1 5.746629e-136 1.9743326 0.807 0.222 9.675025e-132

Ccnd1 9.707790e-134 2.1473020 0.711 0.163 1.634403e-129

Cald1 5.232297e-129 1.7326209 0.741 0.156 8.809096e-125

Prkg2 6.584470e-117 1.5801110 0.579 0.106 1.108561e-112

S100a10 5.284820e-115 2.0296250 0.917 0.552 8.897522e-111

Coro1a 3.355929e-114 -2.7189024 0.036 0.745 5.650042e-110

Lmna 1.453663e-112 1.7051134 0.736 0.195 2.447386e-108

Malat1 5.178687e-111 -2.1299677 0.311 0.897 8.718837e-107

Anxa2 1.904200e-110 1.7521788 0.865 0.317 3.205911e-106

Ptprc 1.675159e-109 -3.0532887 0.017 0.712 2.820298e-105

Tm4sf1 2.427089e-108 1.3830293 0.661 0.137 4.086247e-104

Stk17b 1.042632e-105 -2.7160725 0.080 0.736 1.755376e-101

Serpinb6a 1.215978e-104 1.5285634 0.727 0.209 2.047220e-100

Arhgdib 3.999086e-104 -2.4914795 0.091 0.738 6.732861e-100

S100a11 3.985548e-102 1.7218962 0.923 0.530 6.710069e-98

Srgn 1.365039e-98 -3.1564828 0.036 0.688 2.298179e-94

Fos 1.958852e-95 -3.4453654 0.077 0.691 3.297923e-91

Rhoc 7.318176e-95 1.5359665 0.614 0.164 1.232088e-90

H2-K1 3.166499e-94 -1.9665008 0.287 0.837 5.331117e-90

Laptm5 1.896983e-92 -2.4273331 0.028 0.658 3.193760e-88

Cd53 2.411535e-88 -2.2857270 0.028 0.635 4.060061e-84

Nedd4 6.521114e-88 1.4424583 0.653 0.175 1.097895e-83

Mif 1.547816e-86 1.5419668 0.906 0.594 2.605903e-82

Gpx4 3.368322e-86 1.4885760 0.851 0.493 5.670906e-82

Lcp1 3.299991e-85 -2.4454106 0.039 0.628 5.555865e-81

Cyba 4.570164e-84 -2.3896578 0.033 0.625 7.694329e-80

Hspb1 6.244446e-84 1.4589468 0.512 0.114 1.051315e-79

Pgk1 6.698304e-84 1.6772995 0.848 0.456 1.127727e-79

Timp1 1.114383e-82 1.4900905 0.551 0.132 1.876175e-78

Shisa5 3.904188e-81 -2.0348769 0.179 0.725 6.573091e-77

Rac2 4.759131e-80 -2.1494929 0.022 0.601 8.012473e-76

H2-D1 5.425608e-78 -1.4345501 0.364 0.844 9.134554e-74

Zfp36 1.233977e-77 -2.7768068 0.074 0.623 2.077524e-73

Junb 2.914876e-77 -2.1815490 0.499 0.837 4.907485e-73

Xist 9.879515e-77 -2.5099227 0.069 0.639 1.663315e-72

Ppp1r14b 4.301885e-76 1.4718171 0.625 0.234 7.242653e-72

Ptpn18 1.622254e-75 -2.0772402 0.039 0.594 2.731227e-71

Btg1 1.756783e-75 -1.6245702 0.598 0.899 2.957719e-71

Anxa1 2.483996e-75 1.3112842 0.719 0.247 4.182056e-71

Ly6e 5.389892e-74 -1.8070051 0.275 0.768 9.074423e-70

Igfbp4 6.431222e-74 1.7749083 0.656 0.241 1.082761e-69

Nupr1 1.134515e-73 1.4267376 0.532 0.149 1.910070e-69

Ddx5 1.704739e-73 -1.2930995 0.628 0.914 2.870099e-69

Jund 1.940590e-73 -1.9640263 0.416 0.814 3.267177e-69

Txn1 1.974202e-73 1.2536191 0.873 0.542 3.323766e-69

Id3 3.490273e-73 1.7174599 0.576 0.182 5.876224e-69

Klf2 9.465532e-73 -2.5020085 0.264 0.720 1.593617e-68

Phlda3 1.728772e-72 1.2920812 0.435 0.099 2.910560e-68

Sox9 4.633834e-72 1.2026281 0.493 0.113 7.801523e-68

Tnfrsf12a 1.884684e-71 1.2920767 0.501 0.126 3.173054e-67

Cd74 3.974678e-71 -4.4424280 0.058 0.579 6.691767e-67

2200002D01Rik 4.474225e-71 1.1967305 0.419 0.095 7.532805e-67

Nme2 9.872404e-71 1.2614808 0.906 0.742 1.662118e-66

Ctla2a 2.404649e-70 1.2212208 0.543 0.151 4.048466e-66

Tpi1 5.422973e-70 1.2523945 0.771 0.340 9.130118e-66

Btg2 1.625863e-69 -2.3571327 0.052 0.575 2.737302e-65

Aprt 3.478944e-69 1.3349799 0.777 0.420 5.857150e-65

Pls3 1.090891e-66 1.2554964 0.496 0.129 1.836624e-62

Rps27l 1.680665e-66 1.4538048 0.793 0.467 2.829567e-62

Ank 2.602174e-66 1.1055894 0.534 0.146 4.381020e-62

Nenf 2.502795e-65 1.3016035 0.570 0.197 4.213705e-61

Wwtr1 4.970746e-65 1.1488142 0.444 0.112 8.368749e-61

Anxa5 6.402793e-65 1.4020763 0.744 0.342 1.077974e-60

Cdkn2a 1.424465e-64 1.1872539 0.388 0.084 2.398230e-60

Lmo1 2.977062e-63 1.1500512 0.375 0.084 5.012181e-59

Akap13 4.296955e-63 -1.5959800 0.085 0.585 7.234354e-59

H2-Ab1 5.087440e-63 -3.5962001 0.025 0.515 8.565214e-59

Gmfg 1.447633e-62 -1.8632973 0.017 0.503 2.437235e-58

H2-Aa 4.874561e-62 -3.8039243 0.044 0.523 8.206811e-58

Crip1 8.403135e-62 -2.4279648 0.041 0.528 1.414752e-57

Prdx2 8.834898e-61 1.2654180 0.733 0.383 1.487443e-56

Dap 1.869529e-60 1.3458625 0.603 0.248 3.147539e-56

Ltb 2.565035e-60 -2.0394915 0.019 0.498 4.318493e-56

Ighm 2.617745e-60 -3.1437180 0.033 0.510 4.407236e-56

Lsp1 1.028238e-59 -1.8161260 0.030 0.504 1.731142e-55

Dstn 2.198849e-59 1.2955754 0.642 0.277 3.701981e-55

Zfp36l1 4.126283e-59 -1.7428711 0.314 0.728 6.947010e-55

Ywhae 6.741316e-59 1.1125687 0.843 0.518 1.134968e-54

Tceal9 1.691500e-58 1.2841388 0.598 0.237 2.847810e-54

Uqcc2 5.415502e-58 1.3379481 0.661 0.323 9.117538e-54

B2m 6.848314e-58 -0.9944819 0.755 0.947 1.152982e-53

Clk1 3.549692e-57 -1.5106732 0.107 0.570 5.976262e-53

Cytip 1.269238e-56 -1.7966850 0.011 0.467 2.136889e-52

Eef1b2 2.143374e-56 0.9649961 0.948 0.896 3.608584e-52

H2-Eb1 2.563651e-56 -3.1471363 0.044 0.496 4.316162e-52

Serinc3 2.596442e-56 -1.5599612 0.149 0.606 4.371371e-52

Ptma 6.560175e-56 0.9556268 0.964 0.943 1.104471e-51

Satb1 9.753135e-56 -2.3243822 0.008 0.459 1.642038e-51

Mbnl1 1.145433e-55 -1.4410230 0.421 0.773 1.928452e-51

Cxcr4 1.175180e-55 -2.0780470 0.014 0.466 1.978533e-51

**Cluster 3 macrophage - 1**

cluster.markers <- FindMarkers(danx, ident.1 =3, min.pct = 0.25)

|++++++++++++++++++++++++++++++++++++++++++++++++++| 100% elapsed=09s

> head(cluster.markers, n = 100)

p_val avg_log2FC pct.1 pct.2 p_val_adj

C1qc 3.305416e-202 2.5182313 0.843 0.105 5.564998e-198

C1qa 5.585068e-202 2.7337662 0.881 0.124 9.403021e-198

C1qb 5.781051e-198 2.6299947 0.854 0.116 9.732977e-194

Aif1 7.346758e-179 2.7875494 0.805 0.128 1.236900e-174

Apoe 1.318585e-134 3.0934119 0.935 0.325 2.219970e-130

Lyz2 2.900098e-133 2.1960125 0.874 0.209 4.882605e-129

Fcer1g 2.669431e-132 2.0434442 0.920 0.215 4.494253e-128

Arg1 3.660182e-129 2.6277490 0.605 0.088 6.162282e-125

Pf4 9.452318e-115 2.2501201 0.540 0.074 1.591392e-110

Fabp5 4.042700e-108 2.9440291 0.759 0.224 6.806289e-104

Mmp13 1.780977e-107 2.3361352 0.421 0.043 2.998453e-103

Ftl1 2.043763e-103 2.0801974 0.992 0.838 3.440880e-99

Bcl2a1b 4.727785e-102 2.0918115 0.755 0.215 7.959698e-98

Tyrobp 1.515708e-101 1.5775054 0.877 0.231 2.551846e-97

Fth1 1.859270e-96 1.8411279 1.000 0.981 3.130266e-92

Mmp12 1.193978e-94 2.7199064 0.402 0.047 2.010181e-90

Ccl12 2.250848e-94 2.0861031 0.398 0.045 3.789527e-90

Fcgr3 3.407408e-89 1.7736210 0.621 0.134 5.736712e-85

Tmsb4x 1.944275e-88 1.2225575 1.000 0.998 3.273381e-84

Mt1 2.025259e-88 2.2906447 0.812 0.305 3.409725e-84

Cxcl16 8.140776e-87 1.8286465 0.506 0.090 1.370581e-82

Clec4n 1.767718e-80 1.6244833 0.487 0.088 2.976130e-76

Malat1 2.191423e-80 -2.6308365 0.314 0.872 3.689480e-76

Ms4a7 3.921904e-76 1.7195185 0.391 0.058 6.602918e-72

Ccl24 2.961490e-73 2.5461185 0.326 0.039 4.985965e-69

Msr1 2.409538e-70 1.6622123 0.425 0.076 4.056698e-66

Adamdec1 4.447690e-70 1.6843357 0.287 0.030 7.488131e-66

Mafb 1.693178e-68 1.6344840 0.525 0.124 2.850635e-64

Ctsc 1.231472e-65 1.8202099 0.762 0.337 2.073305e-61

Ctss 5.103896e-60 1.8202502 0.774 0.378 8.592919e-56

Lgmn 1.245866e-59 1.9658553 0.644 0.247 2.097540e-55

Tmsb10 1.027932e-57 -1.6641686 0.368 0.863 1.730626e-53

Ms4a6d 1.429820e-57 1.3659219 0.410 0.085 2.407245e-53

Ccl6 2.441966e-56 1.3748560 0.544 0.147 4.111294e-52

Mrc1 3.504507e-56 1.3110584 0.364 0.066 5.900188e-52

Trf 1.435537e-55 1.6560044 0.467 0.119 2.416871e-51

Ctsz 4.968463e-55 1.7603140 0.670 0.295 8.364904e-51

Klf2 9.219507e-55 -1.9477089 0.142 0.714 1.552196e-50

AW112010 4.946651e-54 1.7042760 0.693 0.306 8.328181e-50

Wfdc17 7.003156e-54 0.6701023 0.571 0.169 1.179051e-49

Dab2 2.823920e-52 1.7315223 0.567 0.205 4.754352e-48

Fcgr4 3.926342e-52 1.4779190 0.372 0.078 6.610389e-48

Ntpcr 5.563109e-52 1.5408374 0.421 0.111 9.366050e-48

Atp6v0c 3.420577e-50 1.3984978 0.793 0.495 5.758884e-46

Cd68 1.340962e-49 1.3577768 0.395 0.096 2.257644e-45

Junb 9.912611e-49 -1.6871838 0.341 0.840 1.668887e-44

Xist 4.283867e-48 -2.3423909 0.107 0.612 7.212319e-44

Npl 3.008948e-47 1.4273069 0.268 0.044 5.065865e-43

Foxp1 8.628217e-47 -1.6051581 0.126 0.640 1.452647e-42

Bcl2a1a 1.003585e-46 1.3521900 0.356 0.081 1.689636e-42

Acp5 2.115966e-46 1.5959210 0.517 0.181 3.562440e-42

Cotl1 2.066492e-44 1.3359229 0.789 0.525 3.479145e-40

Ctsb 2.162865e-42 1.6114683 0.697 0.386 3.641400e-38

Ddx5 2.345796e-42 -1.1318501 0.517 0.913 3.949382e-38

Ms4a6c 2.367701e-42 1.1101981 0.425 0.118 3.986261e-38

Jund 3.380157e-42 -1.5591590 0.356 0.804 5.690832e-38

Fos 3.062850e-39 -2.0900247 0.203 0.653 5.156614e-35

Ets1 3.663482e-39 -1.4894699 0.130 0.603 6.167838e-35

Csf1r 1.339308e-38 1.3094839 0.352 0.091 2.254859e-34

Mpeg1 4.280153e-38 1.2203320 0.395 0.112 7.206065e-34

Sdcbp 1.376361e-37 1.2258867 0.736 0.490 2.317242e-33

Luc7l2 1.439196e-37 -1.2150518 0.161 0.630 2.423031e-33

Prdx5 2.760177e-37 1.3029413 0.770 0.491 4.647035e-33

Mif 1.169356e-36 1.1506997 0.854 0.612 1.968728e-32

Cd300c2 1.421859e-36 1.1450143 0.372 0.105 2.393841e-32

Cst3 4.763771e-36 0.9277905 0.751 0.473 8.020284e-32

Msrb1 4.948409e-36 1.1619586 0.586 0.278 8.331142e-32

Ifitm1 1.595133e-35 1.0054829 0.391 0.111 2.685565e-31

Ccnl1 1.668688e-35 -1.3520146 0.080 0.503 2.809404e-31

Tmem176b 2.025408e-35 1.0613694 0.352 0.096 3.409976e-31

Clk1 2.197371e-34 -1.2752187 0.123 0.549 3.699494e-30

Ucp2 2.199697e-34 1.3064879 0.663 0.374 3.703410e-30

Shisa5 3.527945e-34 -1.2352951 0.249 0.695 5.939648e-30

Gm2a 4.806841e-34 1.4666279 0.594 0.300 8.092797e-30

Eif4a2 1.743719e-33 -1.1114077 0.161 0.599 2.935725e-29

Apoc2 4.848185e-33 1.1558981 0.280 0.067 8.162405e-29

Fcgr1 9.525911e-33 1.1053840 0.261 0.059 1.603782e-28

Psap 1.556838e-32 1.4186400 0.736 0.527 2.621092e-28

Spi1 6.822350e-32 1.0964967 0.471 0.184 1.148611e-27

Btg1 7.519564e-32 -1.1002107 0.582 0.888 1.265994e-27

Rbm39 8.898972e-32 -1.0072729 0.299 0.739 1.498231e-27

Spp1 1.088760e-31 1.1888557 0.628 0.296 1.833036e-27

Selenop 1.365170e-31 1.1383535 0.525 0.228 2.298401e-27

Atox1 1.571587e-31 1.3453497 0.690 0.469 2.645923e-27

Neat1 7.072311e-31 -1.8977412 0.073 0.453 1.190694e-26

Ighm 9.876673e-31 -2.4370925 0.107 0.482 1.662837e-26

Stk17b 1.161986e-30 -1.3242360 0.299 0.687 1.956320e-26

Ltb 2.202117e-30 -1.6297763 0.103 0.469 3.707485e-26

Btg2 4.967242e-30 -1.5831770 0.149 0.544 8.362848e-26

4930523C07Rik 1.186609e-29 -1.2392765 0.092 0.469 1.997775e-25

Gapdh 1.419426e-29 1.0665594 0.751 0.507 2.389745e-25

Gatm 1.739270e-29 1.4279177 0.433 0.184 2.928236e-25

Tsc22d3 1.765257e-29 -1.3082181 0.192 0.591 2.971987e-25

Klf6 4.462732e-29 -1.2921913 0.207 0.603 7.513455e-25

Rps20 5.235039e-29 -0.6814047 0.985 0.972 8.813712e-25

Rps27 2.983408e-28 -0.9728570 0.533 0.873 5.022865e-24

Fosb 8.573922e-28 -1.5693825 0.092 0.445 1.443506e-23

Macf1 1.927646e-27 -1.0055821 0.161 0.550 3.245385e-23

Zfp36l1 2.128664e-27 -1.2055458 0.314 0.711 3.583818e-23

Rpsa 4.420451e-27 -0.6727000 0.916 0.967 7.442271e-23

**Cluster 4 macrophage**

cluster.markers <- FindMarkers(danx, ident.1 = 4, min.pct = 0.25)

|++++++++++++++++++++++++++++++++++++++++++++++++++| 100% elapsed=11s

> head(cluster.markers, n = 100)

p_val avg_log2FC pct.1 pct.2 p_val_adj

Ccr2 7.486681e-215 2.9145359 0.575 0.025 1.260458e-210

Clec4a3 3.995151e-199 1.3411051 0.509 0.018 6.726236e-195

Csf1r 5.822163e-196 1.9521025 0.721 0.061 9.802194e-192

Mpeg1 4.910990e-190 2.1703026 0.783 0.081 8.268143e-186

Ccl9 9.225239e-186 2.4519573 0.619 0.044 1.553161e-181

Ccr5 1.183972e-182 1.6064800 0.602 0.040 1.993336e-178

Cfp 4.432870e-180 1.5308875 0.655 0.052 7.463180e-176

Plbd1 1.711718e-174 1.4797275 0.659 0.053 2.881848e-170

Ms4a6c 1.191477e-172 2.2370145 0.765 0.092 2.005971e-168

C3ar1 1.335630e-170 1.4401350 0.553 0.035 2.248667e-166

Itgam 1.741263e-168 1.5045052 0.664 0.057 2.931591e-164

Sirpa 2.050092e-167 1.6663228 0.770 0.090 3.451534e-163

Pirb 4.037123e-167 1.2538800 0.708 0.066 6.796901e-163

Csf2rb 1.590983e-163 1.3550568 0.664 0.061 2.678580e-159

Cysltr1 3.027248e-160 0.8698410 0.442 0.018 5.096675e-156

Pld4 7.392933e-158 1.4095212 0.681 0.069 1.244674e-153

Csf2ra 3.005411e-154 0.9972525 0.588 0.047 5.059911e-150

Adgre1 3.409373e-152 1.2237033 0.527 0.036 5.740021e-148

Fcgr1 1.331156e-151 1.1977137 0.535 0.038 2.241135e-147

Alox5ap 3.775033e-151 1.6002660 0.699 0.076 6.355645e-147

Mrc1 1.628502e-148 2.1025250 0.575 0.051 2.741746e-144

Cd68 2.062121e-146 1.3431984 0.677 0.075 3.471788e-142

Csf2rb2 6.699180e-146 0.7641120 0.460 0.026 1.127874e-141

Ms4a6d 1.212897e-144 1.5957532 0.646 0.069 2.042034e-140

Fcgr2b 2.196198e-144 2.0141627 0.726 0.102 3.697519e-140

Olfm1 3.316754e-144 1.0799251 0.504 0.036 5.584086e-140

Clec4a1 6.277212e-143 0.9458184 0.314 0.006 1.056831e-138

Rassf4 3.652358e-142 0.9525945 0.478 0.031 6.149110e-138

Clec10a 1.926517e-140 1.2106961 0.292 0.004 3.243485e-136

Cd300c2 1.746008e-139 1.4690636 0.681 0.081 2.939579e-135

Pid1 5.268490e-139 1.2912431 0.451 0.029 8.870030e-135

Lair1 1.697111e-134 1.4961844 0.540 0.050 2.857255e-130

Spint1 1.980834e-134 0.9942573 0.434 0.026 3.334933e-130

Tbxas1 6.277205e-133 0.7521968 0.385 0.018 1.056830e-128

Trem2 7.846557e-133 1.1022519 0.513 0.042 1.321046e-128

Mertk 1.667623e-132 0.6128315 0.332 0.011 2.807610e-128

Cx3cr1 5.778486e-132 1.5875245 0.447 0.030 9.728660e-128

Clec4a2 9.885102e-131 1.1311011 0.553 0.052 1.664256e-126

Cd300a 6.398784e-128 0.9204561 0.460 0.033 1.077299e-123

Lrrc25 7.792424e-128 0.7269736 0.465 0.033 1.311933e-123

Slamf9 1.599394e-125 0.8186367 0.412 0.025 2.692740e-121

P2ry6 1.173634e-124 0.7201477 0.412 0.026 1.975930e-120

F13a1 5.066055e-123 2.0687301 0.394 0.024 8.529211e-119

Lilrb4a 5.236811e-123 1.0314705 0.642 0.077 8.816694e-119

Fcer1g 2.433441e-122 2.1171333 0.920 0.225 4.096941e-118

Tyrobp 1.689323e-120 2.0248637 0.934 0.235 2.844145e-116

Ccl6 1.378329e-119 2.2667333 0.761 0.133 2.320556e-115

Tmem176a 1.055863e-118 1.5931604 0.584 0.072 1.777651e-114

Tnfsf13 1.363595e-118 0.7179979 0.363 0.019 2.295749e-114

Apoc2 2.062266e-118 1.3179024 0.509 0.049 3.472031e-114

Igsf6 1.644229e-116 0.7239505 0.465 0.039 2.768223e-112

Hfe 3.803651e-116 0.7009115 0.358 0.020 6.403826e-112

Msr1 4.678772e-115 1.4180168 0.571 0.068 7.877181e-111

Emilin2 6.118121e-115 1.0818534 0.558 0.063 1.030047e-110

Grn 8.075205e-114 1.8987826 0.845 0.212 1.359542e-109

F10 1.130672e-113 0.7398188 0.270 0.007 1.903599e-109

Dhrs3 1.616170e-113 0.8056992 0.447 0.039 2.720984e-109

Trf 1.929105e-113 1.9421374 0.664 0.106 3.247840e-109

Naip2 1.935364e-113 0.6189266 0.345 0.018 3.258378e-109

Lyz2 1.883057e-112 3.3088760 0.810 0.223 3.170315e-108

C5ar1 7.492212e-112 1.0384898 0.527 0.056 1.261389e-107

Mafb 1.120684e-111 2.2439369 0.664 0.117 1.886783e-107

Slc8a1 2.520874e-111 0.6348464 0.341 0.018 4.244143e-107

Naaa 3.805889e-111 1.4157431 0.535 0.066 6.407595e-107

Tifab 8.546939e-111 0.5648720 0.385 0.025 1.438963e-106

Zeb2 2.694251e-110 1.8979495 0.805 0.204 4.536042e-106

Atf3 6.300058e-110 1.5597199 0.558 0.074 1.060678e-105

Gda 2.125809e-109 1.2230504 0.704 0.112 3.579012e-105

Cst3 8.317356e-108 2.9741301 0.956 0.459 1.400310e-103

Psap 2.649459e-106 2.4814925 0.973 0.509 4.460629e-102

Slc11a1 3.830757e-106 0.6752896 0.385 0.028 6.449462e-102

Clec4n 1.287318e-105 1.4142280 0.597 0.084 2.167329e-101

Tmem176b 2.150540e-105 1.5504018 0.571 0.080 3.620649e-101

Cd302 9.348497e-105 0.8988874 0.491 0.053 1.573913e-100

Fes 2.590244e-104 0.6619256 0.469 0.047 4.360935e-100

Evi2a 5.313906e-104 0.9955980 0.602 0.090 8.946492e-100

Hacd4 6.284761e-104 0.9022710 0.473 0.051 1.058102e-99

Ctss 8.638267e-104 2.2527596 0.934 0.370 1.454339e-99

Cebpa 9.764370e-104 0.6890983 0.398 0.033 1.643929e-99

Shtn1 1.164979e-103 0.5390791 0.363 0.024 1.961358e-99

Tmem106a 5.841985e-103 0.8174694 0.438 0.044 9.835566e-99

Slc15a3 7.214760e-103 0.7079078 0.465 0.046 1.214677e-98

Sirpb1c 1.226463e-102 0.7773722 0.456 0.044 2.064874e-98

Cybb 6.256996e-102 1.7429814 0.832 0.205 1.053428e-97

Ifi30 7.242782e-102 1.9466142 0.867 0.252 1.219395e-97

Ifi207 8.320820e-102 1.0003418 0.500 0.062 1.400893e-97

Tgfbi 1.869721e-101 2.1592460 0.765 0.192 3.147863e-97

Rab3il1 1.756092e-99 0.7132039 0.434 0.042 2.956557e-95

Nfam1 6.723534e-99 0.7170652 0.504 0.057 1.131974e-94

Cd14 1.558101e-98 1.1718052 0.606 0.102 2.623220e-94

Ccr1 1.586948e-97 1.0931140 0.624 0.095 2.671785e-93

Ctsz 2.186947e-97 1.7999442 0.898 0.280 3.681945e-93

Ccrl2 3.221208e-97 0.6805951 0.425 0.041 5.423227e-93

Fcgr4 4.320661e-97 1.3060132 0.522 0.069 7.274265e-93

Wfdc17 8.924400e-97 1.1716743 0.765 0.157 1.502512e-92

P2ry14 2.033049e-96 0.5828204 0.274 0.012 3.422841e-92

Fcgr3 3.558025e-96 1.6357674 0.686 0.135 5.990290e-92

Gpr35 6.533225e-96 0.6093655 0.350 0.026 1.099934e-91

Nxpe5 1.512366e-95 0.4809150 0.257 0.010 2.546219e-91

Lilr4b 5.623050e-95 0.5997676 0.504 0.060 9.466967e-91

**Cluster 5 CD8 Tcells**

cluster.markers <- FindMarkers(danx, ident.1 = 5, min.pct = 0.25)

|++++++++++++++++++++++++++++++++++++++++++++++++++| 100% elapsed=07s

> head(cluster.markers, n = 100)

p_val avg_log2FC pct.1 pct.2 p_val_adj

Nkg7 1.797409e-218 2.5869612 0.635 0.032 3.026117e-214

Il2rb 1.844106e-190 1.9345172 0.502 0.019 3.104737e-186

Cd8b1 3.758711e-156 1.9407314 0.576 0.044 6.328166e-152

Cd8a 6.133212e-150 1.6152904 0.507 0.033 1.032588e-145

Klrd1 1.440056e-135 1.6949052 0.547 0.048 2.424478e-131

Ctsw 2.715481e-128 0.9896830 0.389 0.019 4.571784e-124

Cd3d 2.085061e-123 1.8589572 0.892 0.177 3.510409e-119

Ccl5 3.143120e-122 4.3761170 0.389 0.022 5.291757e-118

Cst7 3.011525e-108 0.8666569 0.315 0.014 5.070204e-104

Ms4a4b 3.038403e-106 1.9414231 0.847 0.196 5.115455e-102

Trbc2 3.068431e-105 1.7636314 0.872 0.182 5.166011e-101

Cd3g 1.651908e-103 1.6231071 0.749 0.138 2.781152e-99

Cd3e 7.142187e-89 1.3160819 0.640 0.114 1.202459e-84

Cxcr6 3.238233e-88 0.9278595 0.251 0.011 5.451889e-84

Lck 1.335137e-87 1.2840771 0.606 0.103 2.247837e-83

Il7r 3.733415e-87 1.8032607 0.700 0.147 6.285577e-83

Itk 2.794563e-83 1.2086348 0.645 0.120 4.704927e-79

Skap1 2.328440e-82 1.2049130 0.626 0.115 3.920162e-78

Ly6c2 5.470845e-82 1.6385490 0.389 0.042 9.210715e-78

Thy1 3.030406e-80 1.3152608 0.631 0.124 5.101992e-76

Bcl11b 3.562937e-80 1.2128490 0.714 0.148 5.998561e-76

Cd28 2.177711e-78 1.2659155 0.719 0.154 3.666394e-74

Cd27 2.414658e-78 1.1377827 0.517 0.081 4.065319e-74

Trac 4.661573e-77 1.2989683 0.655 0.134 7.848225e-73

Tcf7 2.952054e-75 1.3970989 0.773 0.200 4.970079e-71

H2-Q7 1.836844e-71 1.4358195 0.911 0.346 3.092511e-67

Sidt1 4.139455e-71 0.8462950 0.369 0.043 6.969186e-67

Klk8 1.979847e-70 1.0412938 0.468 0.075 3.333271e-66

Icos 2.679938e-70 1.3861800 0.340 0.037 4.511944e-66

Lat 3.002572e-68 1.1498776 0.576 0.116 5.055130e-64

Txk 6.802237e-68 1.4882869 0.606 0.137 1.145225e-63

Trbc1 5.570475e-67 1.8244809 0.557 0.116 9.378452e-63

Ptpn22 2.289789e-66 1.3484259 0.635 0.159 3.855088e-62

Sh2d1a 2.529465e-66 1.1580468 0.365 0.046 4.258608e-62

Hcst 4.295772e-63 1.3323463 0.783 0.249 7.232361e-59

Cd6 1.667133e-61 1.0507139 0.399 0.061 2.806785e-57

Gm15472 3.515611e-60 1.1432128 0.453 0.083 5.918882e-56

Ccnd2 4.390004e-60 1.2457917 0.759 0.237 7.391011e-56

Gm2682 6.528843e-60 0.8546540 0.409 0.064 1.099196e-55

Cd226 1.566299e-59 0.8873650 0.350 0.047 2.637020e-55

Ctla4 1.840287e-59 1.6381069 0.251 0.023 3.098308e-55

Epsti1 9.068090e-59 1.4759239 0.655 0.189 1.526704e-54

Selplg 3.697330e-58 1.2159510 0.709 0.221 6.224826e-54

Dapl1 5.141642e-56 1.4539814 0.419 0.075 8.656469e-52

Emb 1.691361e-55 1.3611199 0.808 0.292 2.847576e-51

AW112010 8.046753e-55 1.7230254 0.803 0.306 1.354751e-50

Ikzf2 4.365437e-53 2.0146699 0.335 0.054 7.349650e-49

Fth1 1.455623e-52 -2.2487493 0.961 0.985 2.450686e-48

Zap70 3.391000e-52 0.7092115 0.325 0.046 5.709087e-48

Themis 1.834259e-51 0.8511026 0.453 0.089 3.088159e-47

Ms4a6b 4.873447e-51 1.0781804 0.778 0.263 8.204935e-47

Prkch 1.121145e-50 0.8136565 0.424 0.081 1.887560e-46

Il18r1 1.726591e-50 0.7751585 0.261 0.030 2.906889e-46

Cd96 1.644282e-49 0.7138430 0.281 0.036 2.768314e-45

Lef1 2.458165e-48 0.9202443 0.645 0.178 4.138567e-44

Sh2d2a 5.633531e-47 0.8654079 0.365 0.065 9.484612e-43

Gimap4 5.141462e-46 1.1156133 0.680 0.218 8.656165e-42

Tnfaip3 1.399066e-45 1.1286807 0.601 0.182 2.355467e-41

Gimap3 4.901707e-45 1.0409444 0.645 0.200 8.252514e-41

Tnfrsf18 1.557915e-44 0.9384438 0.291 0.044 2.622906e-40

Ipcef1 2.105988e-44 0.8316567 0.429 0.093 3.545642e-40

Cd2 3.650443e-44 0.9512766 0.547 0.146 6.145887e-40

Gm43065 2.285188e-43 0.8365562 0.315 0.055 3.847343e-39

Spn 8.790210e-43 0.8765666 0.419 0.095 1.479920e-38

Fam189b 1.169464e-42 0.8609595 0.369 0.077 1.968910e-38

Plcxd2 3.574763e-42 0.6287656 0.315 0.054 6.018471e-38

Pdcd4 3.757007e-42 1.0565237 0.739 0.301 6.325297e-38

Nsg2 7.413502e-39 0.7176283 0.360 0.074 1.248137e-34

Prkcq 1.181984e-38 0.7805959 0.424 0.101 1.989989e-34

Ptprc 2.577238e-37 0.9445189 0.961 0.594 4.339038e-33

Cd247 5.133744e-36 0.7851279 0.379 0.088 8.643171e-32

H2-K1 6.888119e-36 0.9124761 0.966 0.749 1.159684e-31

Mbnl1 1.277516e-35 0.9663068 0.946 0.709 2.150826e-31

Cd5 1.303212e-34 0.6981174 0.330 0.071 2.194088e-30

Ablim1 1.405609e-34 0.8365646 0.833 0.360 2.366484e-30

Atp8b4 1.899899e-34 0.6057795 0.261 0.046 3.198670e-30

Grap2 2.316302e-34 0.7646930 0.493 0.144 3.899726e-30

Inpp4b 1.970151e-33 0.9998466 0.522 0.176 3.316946e-29

Rinl 2.089483e-33 0.6775331 0.365 0.089 3.517854e-29

Tmsb10 7.795654e-33 0.8012428 0.970 0.804 1.312476e-28

Vps37b 1.151636e-32 0.9220883 0.670 0.290 1.938895e-28

H2-Q6 1.156005e-32 0.7823813 0.478 0.145 1.946251e-28

Stat4 4.157875e-32 0.7794018 0.330 0.076 7.000198e-28

Peli1 4.268284e-32 1.0451075 0.680 0.326 7.186083e-28

Saraf 4.613236e-32 0.9344158 0.650 0.297 7.766845e-28

Fyb 7.622956e-32 0.8714329 0.640 0.257 1.283401e-27

Il27ra 8.395746e-32 0.6200305 0.286 0.058 1.413508e-27

Ubash3a 3.020666e-31 0.6355767 0.251 0.046 5.085593e-27

Ptpn18 7.917389e-31 0.8100052 0.901 0.491 1.332972e-26

Rapgef6 8.427589e-31 0.9722422 0.729 0.366 1.418869e-26

H2-Ab1 8.951845e-31 -3.3533046 0.059 0.481 1.507133e-26

Rpl18 9.853667e-31 0.6353543 0.990 0.951 1.658963e-26

Hsd11b1 1.259433e-30 0.5581681 0.271 0.055 2.120381e-26

Gramd3 2.767235e-30 0.8343842 0.448 0.144 4.658917e-26

Shisa5 3.811612e-30 0.8360219 0.926 0.632 6.417230e-26

Itpkb 1.047163e-29 0.8269502 0.537 0.197 1.763004e-25

Ppm1h 1.996704e-29 0.6440226 0.350 0.094 3.361651e-25

Smc4 2.285514e-29 0.9668481 0.670 0.312 3.847891e-25

Fyn 3.808444e-29 0.8536446 0.468 0.164 6.411896e-25

Rps16 4.077547e-29 0.5904733 0.990 0.984 6.864959e-25

**Cluster 6 Cancer cells 2**

cluster.markers <- FindMarkers(danx, ident.1 = 6, min.pct = 0.25)

|++++++++++++++++++++++++++++++++++++++++++++++++++| 100% elapsed=23s

> head(cluster.markers, n = 100)

p_val avg_log2FC pct.1 pct.2 p_val_adj

Lamc2 5.130054e-305 1.6788764 0.781 0.023 8.636959e-301

Fbln2 4.996883e-296 2.5153850 0.975 0.052 8.412752e-292

Ncam1 3.561863e-290 1.6088236 0.887 0.041 5.996752e-286

Lama5 7.392361e-288 1.8453435 0.944 0.053 1.244578e-283

Lgr6 5.198722e-287 1.4360754 0.869 0.039 8.752568e-283

Tfap2b 7.085840e-282 1.1190043 0.744 0.023 1.192972e-277

Igfbp3 4.191370e-278 2.3842360 0.869 0.041 7.056591e-274

Neo1 1.762826e-277 0.9993299 0.856 0.040 2.967893e-273

Ptprk 1.356073e-276 1.0699423 0.844 0.039 2.283085e-272

Unc5b 1.069312e-268 1.3260215 0.894 0.048 1.800293e-264

Pcdh7 4.470323e-265 1.7038695 0.887 0.051 7.526237e-261

Adgrl3 5.790160e-262 1.3122846 0.844 0.044 9.748313e-258

Efnb1 1.184621e-257 1.5135869 0.881 0.053 1.994427e-253

Itgb4 2.365199e-257 0.9425392 0.662 0.019 3.982048e-253

Slco2a1 1.415761e-256 2.1991599 0.938 0.065 2.383575e-252

Mir100hg 8.268627e-254 1.8081121 0.919 0.062 1.392106e-249

Ano1 1.329677e-253 1.4971401 0.925 0.057 2.238645e-249

Pxdn 8.524040e-251 1.1673408 0.863 0.048 1.435107e-246

Msln 1.509300e-250 1.0105375 0.656 0.019 2.541057e-246

Tnc 1.228283e-249 1.7032615 0.713 0.027 2.067938e-245

Bdnf 1.100669e-248 1.3706195 0.831 0.045 1.853086e-244

Clu 1.467981e-246 2.4081440 0.938 0.069 2.471492e-242

Gm14636 6.603362e-246 0.9847173 0.731 0.030 1.111742e-241

Ccbe1 1.094086e-244 1.2506062 0.856 0.050 1.842003e-240

Jag1 4.418768e-244 2.2903147 0.938 0.070 7.439438e-240

Pgf 3.208494e-243 1.2462210 0.825 0.046 5.401821e-239

Itga3 2.253798e-242 1.3961888 0.806 0.043 3.794494e-238

Tns4 2.719452e-240 1.5863827 0.900 0.060 4.578470e-236

Vldlr 2.821225e-240 0.7577602 0.606 0.016 4.749815e-236

Cpe 1.255397e-239 2.5035452 0.988 0.084 2.113586e-235

Magi1 4.174426e-239 0.7569803 0.762 0.036 7.028064e-235

Piezo2 1.014856e-238 1.9104444 0.881 0.059 1.708612e-234

Smoc2 3.632847e-238 2.5423409 0.969 0.082 6.116261e-234

Ltbp1 7.507251e-238 1.7166581 0.831 0.051 1.263921e-233

Ackr3 5.064764e-237 1.1066641 0.856 0.052 8.527037e-233

Lamb1 3.544285e-235 2.0049766 0.981 0.082 5.967158e-231

Sdc1 1.737663e-233 1.9502032 0.956 0.079 2.925530e-229

Adgrg6 1.803362e-233 1.1414101 0.744 0.036 3.036140e-229

Slc47a1 2.205927e-233 0.8409313 0.706 0.030 3.713899e-229

Bmp7 2.487010e-231 1.0522356 0.800 0.044 4.187130e-227

Pkp1 5.808508e-230 1.2005494 0.812 0.048 9.779204e-226

Bmp2 1.700371e-228 1.8714224 0.875 0.059 2.862744e-224

Tfap2a 6.551656e-228 0.8637825 0.800 0.045 1.103037e-223

Nav2 1.896357e-226 1.0537049 0.850 0.054 3.192706e-222

Flt1 4.087071e-226 0.9744887 0.731 0.035 6.880992e-222

Tmprss11e 5.761814e-225 0.6819822 0.444 0.004 9.700591e-221

Wnt5a 8.058202e-224 1.1667449 0.844 0.056 1.356679e-219

Col18a1 1.113508e-223 2.1267655 0.938 0.075 1.874703e-219

Gja1 6.371827e-223 1.0240103 0.856 0.057 1.072761e-218

Epha2 3.292306e-222 0.8753976 0.762 0.042 5.542926e-218

Has2 4.350734e-222 1.1123451 0.688 0.031 7.324895e-218

Mab21l4 3.144671e-221 0.8122115 0.600 0.019 5.294368e-217

Acvr1 6.546220e-221 0.8916899 0.787 0.046 1.102122e-216

Angptl2 1.622611e-220 1.4883229 0.900 0.068 2.731828e-216

Abcb1a 2.426844e-218 1.3208608 0.838 0.057 4.085835e-214

Ahnak2 6.989387e-217 1.1306013 0.838 0.058 1.176733e-212

Tnfrsf23 5.197559e-215 0.9949921 0.887 0.066 8.750610e-211

Selp 1.327349e-214 0.9903695 0.475 0.008 2.234724e-210

Inhba 4.305094e-214 1.9828699 0.919 0.077 7.248055e-210

Col7a1 8.959584e-214 1.1208285 0.750 0.043 1.508436e-209

Areg 2.882283e-213 2.7974220 0.912 0.082 4.852611e-209

Rdh10 4.384293e-213 0.9737978 0.806 0.053 7.381396e-209

Epn2 1.477432e-211 1.0480943 0.850 0.062 2.487405e-207

Fgfr1 1.831356e-211 1.9462597 0.981 0.100 3.083272e-207

Pcdh19 4.483609e-211 0.8117971 0.619 0.023 7.548604e-207

Ptprs 4.038874e-210 1.5150024 0.931 0.085 6.799848e-206

Sema3a 7.666798e-210 0.8203571 0.637 0.028 1.290782e-205

Klra4 9.511685e-210 1.4794124 0.825 0.057 1.601387e-205

Tbx3 1.104466e-209 0.7149889 0.731 0.041 1.859479e-205

Cadm1 8.624313e-208 1.0225103 0.775 0.050 1.451989e-203

Plod2 1.636787e-206 1.3763156 0.900 0.072 2.755694e-202

Fnbp1l 3.409132e-206 0.9685685 0.869 0.068 5.739614e-202

Ghr 2.262448e-205 1.4833689 0.925 0.084 3.809058e-201

Etv1 4.474833e-205 1.1607311 0.875 0.072 7.533830e-201

Bmp1 5.111999e-205 1.4792674 0.863 0.067 8.606562e-201

Prrx1 6.013640e-205 0.7822096 0.781 0.047 1.012456e-200

Htra1 6.800711e-205 1.2473065 0.856 0.067 1.144968e-200

Lamc1 2.679756e-204 1.4054391 0.887 0.074 4.511637e-200

Cdh2 2.844672e-204 0.8257599 0.744 0.044 4.789290e-200

Abca5 3.591369e-204 0.6341575 0.644 0.030 6.046428e-200

Zfp503 9.062269e-204 0.9161075 0.769 0.050 1.525724e-199

B3gnt3 1.363903e-203 0.5799887 0.644 0.030 2.296268e-199

Clstn1 2.040750e-203 0.8037746 0.744 0.046 3.435807e-199

Gprc5a 6.221997e-203 0.7722834 0.662 0.033 1.047535e-198

Cxadr 1.887710e-201 0.6509931 0.650 0.031 3.178149e-197

Map1b 7.274232e-201 1.3546224 0.925 0.084 1.224690e-196

Asap2 2.017407e-200 1.3014721 0.887 0.077 3.396506e-196

Arhgef40 1.390884e-198 0.9331330 0.850 0.067 2.341692e-194

Dcbld2 1.558435e-198 0.9227289 0.812 0.059 2.623781e-194

Ereg 2.474331e-198 0.7875364 0.600 0.025 4.165783e-194

Flnb 6.827685e-198 1.4673833 0.931 0.089 1.149509e-193

Hspg2 1.217089e-197 1.0573382 0.831 0.062 2.049090e-193

Ltbp3 2.911937e-197 1.6636338 0.931 0.092 4.902537e-193

Pkd2 2.743820e-196 0.8123280 0.744 0.049 4.619495e-192

Nppb 1.492874e-195 1.9230758 0.700 0.042 2.513402e-191

Gjb4 9.401103e-194 0.4910232 0.531 0.018 1.582770e-189

Frmd4a 1.872328e-193 1.4720788 0.944 0.098 3.152252e-189

Flrt3 1.809570e-192 0.5661047 0.494 0.013 3.046592e-188

Slc35e4 2.748369e-192 0.7989479 0.781 0.056 4.627154e-188

Col4a1 9.006539e-191 0.9790314 0.894 0.076 1.516341e-186

**Cluster 7 neutrophils**

cluster.markers <- FindMarkers(danx, ident.1 = 7, min.pct = 0.25)

|++++++++++++++++++++++++++++++++++++++++++++++++++| 100% elapsed=15s

> head(cluster.markers, n = 100)

p_val avg_log2FC pct.1 pct.2 p_val_adj

Cxcr2 0.000000e+00 3.301580 0.788 0.001 0.000000e+00

Hdc 0.000000e+00 3.241031 0.689 0.006 0.000000e+00

S100a9 0.000000e+00 8.891881 0.887 0.033 0.000000e+00

Csf3r 0.000000e+00 3.568157 0.868 0.015 0.000000e+00

Wfdc21 0.000000e+00 4.278426 0.669 0.006 0.000000e+00

Stfa2l1 0.000000e+00 3.617743 0.543 0.000 0.000000e+00

Retnlg 0.000000e+00 6.815801 0.675 0.011 0.000000e+00

Cxcl2 8.310141e-304 6.007811 0.901 0.042 1.399095e-299

Mmp9 1.319218e-294 3.371336 0.722 0.018 2.221035e-290

Lcn2 3.671810e-275 4.208900 0.642 0.014 6.181860e-271

S100a8 9.917541e-264 8.500034 0.901 0.060 1.669717e-259

Clec4e 2.662691e-263 3.237590 0.675 0.020 4.482907e-259

Cstdc4 7.920616e-258 4.088434 0.470 0.002 1.333515e-253

Lrg1 8.293291e-252 2.785224 0.570 0.011 1.396259e-247

Asprv1 3.209148e-246 2.937711 0.457 0.002 5.402921e-242

Slpi 4.630684e-246 3.324865 0.728 0.031 7.796220e-242

G0s2 3.439697e-243 4.861974 0.550 0.010 5.791074e-239

Clec4d 1.860095e-229 2.999660 0.702 0.032 3.131656e-225

Hp 1.040243e-213 2.973029 0.636 0.027 1.751353e-209

Acod1 1.192564e-206 2.507072 0.417 0.004 2.007801e-202

Fpr2 5.733389e-204 1.942639 0.483 0.010 9.652734e-200

Il1f9 1.731623e-201 1.408045 0.358 0.001 2.915360e-197

Il1r2 8.071814e-196 3.168504 0.722 0.048 1.358971e-191

Trem1 1.208273e-194 2.545292 0.629 0.032 2.034248e-190

Stfa2 6.578978e-194 2.670737 0.331 0.000 1.107637e-189

Ifitm1 3.255564e-189 4.234558 0.894 0.094 5.481067e-185

Slfn4 6.189358e-188 1.864329 0.391 0.005 1.042040e-183

Chil1 2.610615e-187 1.463286 0.358 0.002 4.395232e-183

Mxd1 2.443809e-183 3.123470 0.828 0.085 4.114397e-179

Alox5ap 7.789460e-180 3.531527 0.834 0.086 1.311434e-175

Arg2 1.942139e-179 1.501150 0.371 0.004 3.269786e-175

Cd300lf 2.634680e-176 2.518634 0.702 0.054 4.435748e-172

Mcemp1 8.420277e-165 2.009582 0.530 0.026 1.417638e-160

Mmp8 1.320122e-164 3.165060 0.384 0.008 2.222558e-160

Il1b 1.153754e-161 5.279506 0.854 0.114 1.942461e-157

Hcar2 3.424737e-160 2.312555 0.411 0.011 5.765887e-156

Lilrb4a 2.173690e-159 2.594476 0.781 0.085 3.659625e-155

Cd33 5.761241e-154 2.731565 0.715 0.073 9.699626e-150

Ccr1 1.950513e-153 2.795680 0.815 0.099 3.283884e-149

Lilr4b 4.801165e-153 2.859834 0.682 0.063 8.083241e-149

Mirt2 4.135743e-147 1.179002 0.258 0.000 6.962936e-143

Cd177 1.637688e-143 1.585569 0.298 0.003 2.757211e-139

Pglyrp1 1.955334e-139 2.836148 0.636 0.060 3.292001e-135

Osm 7.198086e-127 3.276632 0.517 0.039 1.211870e-122

Tyrobp 2.882495e-125 2.964276 1.000 0.251 4.852969e-121

Nfam1 8.989679e-124 2.229136 0.616 0.064 1.513502e-119

Cd300ld 1.532886e-122 1.903398 0.523 0.041 2.580767e-118

Ankrd33b 6.178968e-120 1.300985 0.311 0.008 1.040291e-115

C5ar1 2.127145e-117 2.011470 0.609 0.065 3.581262e-113

Bst1 1.165424e-115 1.728576 0.437 0.028 1.962107e-111

Egr1 2.525292e-114 3.918077 0.861 0.190 4.251581e-110

Slc40a1 7.623931e-112 1.655084 0.364 0.017 1.283565e-107

Cd14 1.347845e-109 3.538852 0.702 0.111 2.269232e-105

Gsr 3.103168e-109 2.682593 0.841 0.188 5.224494e-105

Slc7a11 2.214032e-108 2.193829 0.437 0.031 3.727543e-104

Pygl 2.270952e-108 2.083951 0.556 0.059 3.823375e-104

Gda 4.639159e-108 2.621174 0.742 0.126 7.810488e-104

Chil3 6.305948e-108 2.954012 0.397 0.023 1.061669e-103

Trim30b 2.343961e-107 1.631358 0.437 0.031 3.946293e-103

Msrb1 1.290469e-104 3.044801 0.921 0.272 2.172633e-100

Ccrl2 1.128512e-103 2.987967 0.497 0.047 1.899963e-99

Dusp1 7.155050e-101 4.028283 0.967 0.367 1.204624e-96

Wfdc17 1.989066e-98 4.272861 0.788 0.173 3.348791e-94

Gm9733 5.396365e-98 1.386217 0.325 0.016 9.085320e-94

Ptafr 1.073651e-96 2.048403 0.437 0.037 1.807598e-92

Cebpb 2.192310e-96 2.227762 0.682 0.116 3.690973e-92

Itgam 7.784143e-96 2.035519 0.589 0.078 1.310538e-91

Fcer1g 1.451826e-95 2.161304 0.980 0.241 2.444295e-91

Grina 2.379462e-93 2.863083 0.821 0.223 4.006063e-89

Ifitm6 1.151018e-92 3.295757 0.384 0.028 1.937854e-88

Mcl1 1.539717e-89 2.798343 0.993 0.551 2.592267e-85

Srgn 4.455917e-89 3.065175 0.987 0.581 7.501981e-85

F630028O10Rik 2.638218e-88 1.479152 0.285 0.013 4.441704e-84

Siglece 1.024501e-87 1.329498 0.344 0.023 1.724850e-83

Fgr 2.093674e-85 1.716405 0.517 0.065 3.524910e-81

Ppp1r3b 2.351442e-85 1.297582 0.351 0.025 3.958887e-81

Pim1 4.046354e-85 3.137019 0.901 0.335 6.812441e-81

Dhrs9 1.418936e-84 0.942291 0.272 0.012 2.388921e-80

Rps20 2.020386e-84 -3.006973 0.629 0.993 3.401522e-80

Ier3 6.383253e-84 3.476357 0.795 0.211 1.074685e-79

Il18rap 2.340067e-83 1.650678 0.457 0.052 3.939736e-79

Rpl13 1.459878e-82 -2.587860 0.828 0.994 2.457851e-78

Rps4x 3.450687e-81 -2.852966 0.517 0.981 5.809577e-77

Pirb 1.933209e-80 1.777327 0.576 0.092 3.254751e-76

Eef1a1 7.582466e-80 -2.210359 0.834 0.996 1.276584e-75

Rpl12 1.840795e-79 -3.072607 0.530 0.980 3.099163e-75

Rpsa 2.169931e-79 -2.645024 0.623 0.981 3.653296e-75

Ifitm2 2.183633e-79 2.788079 0.901 0.344 3.676364e-75

Rpl3 2.674553e-79 -2.888962 0.325 0.962 4.502877e-75

Rps8 3.421192e-79 -2.331277 0.735 0.988 5.759919e-75

Rpl23 4.142961e-79 -2.243854 0.894 0.992 6.975089e-75

Rps24 8.552865e-79 -2.697470 0.881 0.994 1.439960e-74

Rpl10a 8.728597e-79 -2.893325 0.305 0.963 1.469547e-74

Rpl32 1.499487e-78 -2.571325 0.702 0.984 2.524537e-74

Lst1 1.796817e-78 2.309246 0.656 0.125 3.025122e-74

Rpl13a 2.768446e-78 -2.419251 0.715 0.985 4.660956e-74

Csf2rb 3.085494e-78 1.676154 0.556 0.084 5.194738e-74

Rpl19 3.191713e-78 -2.306523 0.795 0.989 5.373569e-74

Fbxl5 3.198497e-78 2.854528 0.722 0.191 5.384989e-74

Rplp1 1.539616e-77 -2.260156 0.848 0.997 2.592098e-73

**Cluster 8 endothelial cells**

cluster.markers <- FindMarkers(danx, ident.1 = 8, min.pct = 0.25)

|++++++++++++++++++++++++++++++++++++++++++++++++++| 100% elapsed=18s

> head(cluster.markers, n = 100)

p_val avg_log2FC pct.1 pct.2 p_val_adj

Sulf1 0.000000e+00 1.4335735 0.568 0.000 0.000000e+00

Col3a1 0.000000e+00 6.7762674 0.986 0.024 0.000000e+00

Col5a2 0.000000e+00 3.2652187 0.865 0.003 0.000000e+00

Prelp 0.000000e+00 1.9060909 0.622 0.002 0.000000e+00

Dpt 0.000000e+00 4.0216061 0.703 0.001 0.000000e+00

Cd34 0.000000e+00 2.6527245 0.743 0.003 0.000000e+00

Entpd2 0.000000e+00 1.3589611 0.527 0.000 0.000000e+00

Scn7a 0.000000e+00 1.7381422 0.554 0.000 0.000000e+00

Serping1 0.000000e+00 4.3035024 0.851 0.002 0.000000e+00

Fbn1 0.000000e+00 3.8687759 0.878 0.004 0.000000e+00

Cpxm1 0.000000e+00 3.6923896 0.851 0.005 0.000000e+00

Tshz2 0.000000e+00 1.0846617 0.622 0.003 0.000000e+00

Sfrp2 0.000000e+00 3.3119463 0.662 0.003 0.000000e+00

Ctsk 0.000000e+00 2.3830291 0.743 0.004 0.000000e+00

Gstm2 0.000000e+00 1.2595338 0.689 0.004 0.000000e+00

Svep1 0.000000e+00 2.5716228 0.595 0.000 0.000000e+00

Mfap2 0.000000e+00 1.4666867 0.649 0.001 0.000000e+00

Mmp23 0.000000e+00 1.2601177 0.608 0.003 0.000000e+00

Sod3 0.000000e+00 1.5327179 0.635 0.001 0.000000e+00

Adgrd1 0.000000e+00 1.5766964 0.716 0.000 0.000000e+00

Col1a2 0.000000e+00 5.6385465 0.986 0.010 0.000000e+00

Rarres2 0.000000e+00 3.8729920 0.838 0.004 0.000000e+00

Mfap5 0.000000e+00 3.5775689 0.784 0.004 0.000000e+00

C1s1 0.000000e+00 3.2238641 0.838 0.009 0.000000e+00

Fxyd1 0.000000e+00 1.7071344 0.662 0.001 0.000000e+00

Prss23 0.000000e+00 2.9173123 0.811 0.008 0.000000e+00

Serpinh1 0.000000e+00 3.4627089 0.892 0.008 0.000000e+00

Nnmt 0.000000e+00 1.4640802 0.635 0.000 0.000000e+00

Islr 0.000000e+00 1.8762488 0.608 0.001 0.000000e+00

Loxl1 0.000000e+00 2.8572041 0.838 0.002 0.000000e+00

Rbms3 0.000000e+00 1.5274412 0.689 0.003 0.000000e+00

Clec3b 0.000000e+00 3.5134188 0.703 0.003 0.000000e+00

Col6a2 0.000000e+00 2.2236102 0.770 0.008 0.000000e+00

Lum 0.000000e+00 3.5710096 0.797 0.001 0.000000e+00

Aebp1 0.000000e+00 3.6240502 0.892 0.007 0.000000e+00

Grb10 0.000000e+00 1.2243257 0.608 0.002 0.000000e+00

Efemp1 0.000000e+00 3.2568860 0.757 0.000 0.000000e+00

Slit3 0.000000e+00 1.6580718 0.662 0.000 0.000000e+00

Gfpt2 0.000000e+00 1.3986686 0.581 0.001 0.000000e+00

Col1a1 0.000000e+00 5.5226741 0.959 0.021 0.000000e+00

Cavin1 0.000000e+00 2.5260908 0.851 0.008 0.000000e+00

Mrc2 0.000000e+00 0.7856092 0.568 0.001 0.000000e+00

Cygb 0.000000e+00 1.9446561 0.608 0.002 0.000000e+00

Serpina3n 0.000000e+00 2.7849537 0.716 0.001 0.000000e+00

Meg3 0.000000e+00 3.0319598 0.676 0.001 0.000000e+00

Rian 0.000000e+00 1.3601116 0.554 0.001 0.000000e+00

Ogn 0.000000e+00 2.6181922 0.649 0.001 0.000000e+00

Gas1 0.000000e+00 2.4055894 0.662 0.004 0.000000e+00

Scara3 0.000000e+00 1.9402883 0.595 0.001 0.000000e+00

Loxl2 0.000000e+00 1.5386423 0.703 0.001 0.000000e+00

Fbln1 0.000000e+00 1.8584934 0.716 0.001 0.000000e+00

Ccdc80 0.000000e+00 2.7702875 0.824 0.008 0.000000e+00

Abi3bp 0.000000e+00 2.3490779 0.608 0.001 0.000000e+00

Vgll3 0.000000e+00 1.8652341 0.730 0.006 0.000000e+00

Adamts5 0.000000e+00 3.0552373 0.730 0.003 0.000000e+00

Fndc1 0.000000e+00 1.9009050 0.568 0.001 0.000000e+00

Thbs2 0.000000e+00 2.3914495 0.662 0.001 0.000000e+00

Tnxb 0.000000e+00 2.8053319 0.689 0.002 0.000000e+00

Lox 0.000000e+00 3.1870174 0.797 0.001 0.000000e+00

Cd248 0.000000e+00 2.7168927 0.703 0.001 0.000000e+00

Il33 0.000000e+00 1.6863847 0.581 0.001 0.000000e+00

Srpx 0.000000e+00 1.2771288 0.595 0.000 0.000000e+00

Gpc3 0.000000e+00 1.5237458 0.608 0.002 0.000000e+00

Tmem45a 5.860106e-307 0.9368072 0.514 0.000 9.866074e-303

Adamts2 1.943753e-306 2.2669786 0.757 0.010 3.272503e-302

Lgi2 5.272895e-306 1.8387371 0.595 0.003 8.877446e-302

Nid1 2.940387e-301 3.3292312 0.797 0.013 4.950435e-297

Adamts15 4.828784e-299 1.3956653 0.514 0.000 8.129741e-295

Bgn 3.508549e-296 3.9082547 0.892 0.020 5.906993e-292

Adamtsl1 3.354628e-292 0.9274473 0.527 0.001 5.647852e-288

Fkbp10 1.259433e-291 0.9673931 0.527 0.001 2.120381e-287

Hoxc8 1.044335e-290 0.7820579 0.486 0.000 1.758243e-286

C1ra 1.217190e-288 2.3306742 0.797 0.014 2.049260e-284

Lrrc17 1.366975e-282 1.3151188 0.473 0.000 2.301440e-278

Igsf10 2.189349e-282 1.0875626 0.486 0.000 3.685988e-278

Ccl11 3.441974e-275 1.9508656 0.486 0.001 5.794908e-271

Ndn 1.164491e-274 0.7041430 0.486 0.001 1.960537e-270

Spon2 2.505059e-274 1.0169226 0.473 0.000 4.217517e-270

Dcn 8.159521e-274 6.2438555 0.919 0.027 1.373737e-269

Timp3 4.273175e-273 2.8319635 0.595 0.005 7.194317e-269

Thbs3 4.955896e-273 1.4995581 0.595 0.005 8.343746e-269

Medag 8.579973e-273 1.5457798 0.689 0.010 1.444524e-268

Nsg1 3.911830e-270 1.2530440 0.514 0.002 6.585957e-266

Fxyd6 5.224634e-269 1.3773715 0.500 0.001 8.796194e-265

Sfrp4 6.046832e-268 3.0475060 0.486 0.001 1.018045e-263

Prg4 6.147157e-268 2.1018016 0.541 0.003 1.034935e-263

Ptgis 6.360328e-268 1.7189387 0.486 0.001 1.070825e-263

Bicc1 7.197644e-267 1.7363303 0.703 0.011 1.211795e-262

Dpep1 2.410728e-266 1.2953583 0.459 0.000 4.058702e-262

Lama4 4.550189e-264 1.3709379 0.635 0.007 7.660698e-260

Col6a3 5.339488e-264 2.6571697 0.784 0.017 8.989561e-260

Gpx7 2.150285e-261 0.6715112 0.500 0.002 3.620219e-257

Pamr1 2.414124e-258 1.2254912 0.446 0.000 4.064419e-254

Col6a1 2.206546e-256 2.9075347 0.838 0.022 3.714941e-252

Lbp 4.031535e-255 2.6558986 0.595 0.006 6.787493e-251

Serpinf1 1.127474e-254 2.7362840 0.770 0.017 1.898216e-250

Eln 2.117488e-252 1.5214323 0.473 0.001 3.565003e-248

Vcan 5.072300e-252 2.0332087 0.716 0.013 8.539724e-248

Chl1 2.322837e-250 1.3581847 0.432 0.000 3.910728e-246

Osmr 2.685397e-250 1.0863169 0.581 0.006 4.521134e-246

**Cluster 9 Fibroblasts**

| cluster.markers <- FindMarkers(danx, ident.1 = 9, min.pct = 0.25)  \|++++++++++++++++++++++++++++++++++++++++++++++++++\| 100% elapsed=22s  > head(cluster.markers, n = 100)  p_val avg_log2FC pct.1 pct.2 p_val_adj  Col4a3 0.000000e+00 0.7230677 0.725 0.007 0.000000e+00  Epha7 0.000000e+00 0.4976842 0.623 0.002 0.000000e+00  Tbx2 7.522537e-306 1.0695632 0.812 0.011 1.266494e-301  Acsl6 9.380088e-295 0.3820855 0.493 0.000 1.579232e-290  Eda2r 4.517953e-274 0.4787770 0.652 0.007 7.606426e-270  Gm43113 8.805879e-274 0.4499067 0.565 0.003 1.482558e-269  Gm43728 3.512266e-270 0.6319708 0.710 0.010 5.913251e-266  Gm43112 2.488658e-259 0.5467492 0.580 0.005 4.189904e-255  Cdh13 1.033197e-243 0.3255644 0.536 0.004 1.739490e-239  Phactr1 3.633069e-243 0.6933797 0.725 0.014 6.116635e-239  Tafa1 1.029129e-240 0.3537719 0.478 0.002 1.732642e-236  Gm43111 2.956446e-232 0.6693282 0.594 0.008 4.977472e-228  Nes 4.173032e-230 1.3165030 0.928 0.032 7.025716e-226  Tspan7 2.205343e-228 0.9992850 0.797 0.021 3.712916e-224  Fermt1 4.992820e-223 0.7063801 0.696 0.015 8.405911e-219  Col8a1 6.563702e-211 1.2205713 0.623 0.011 1.105065e-206  Ociad2 2.855545e-206 0.4292836 0.609 0.011 4.807595e-202  Gm43727 3.909556e-205 0.6455917 0.536 0.007 6.582128e-201  Gm10484 1.302231e-202 0.3658995 0.478 0.005 2.192435e-198  Naalad2 6.520005e-200 0.3929533 0.536 0.008 1.097708e-195  Sorbs2 1.237954e-196 0.7631532 0.667 0.016 2.084219e-192  Edil3 1.412816e-191 0.4373316 0.551 0.010 2.378617e-187  Gpc4 3.705230e-190 0.4190561 0.551 0.010 6.238125e-186  Ngf 9.236070e-189 0.6805670 0.725 0.023 1.554985e-184  Prokr2 2.325181e-188 0.3491865 0.435 0.004 3.914675e-184  Sema5a 1.903647e-182 2.2327903 0.928 0.048 3.204980e-178  Tenm3 3.603906e-180 1.3794103 0.884 0.043 6.067537e-176  Col4a4 4.233993e-179 0.3981527 0.522 0.009 7.128351e-175  Pdgfrl 2.711203e-178 0.5131323 0.449 0.006 4.564581e-174  Gm43623 7.186960e-175 0.3546871 0.493 0.008 1.209997e-170  Nrn1 7.991284e-173 0.9134940 0.609 0.016 1.345413e-168  Masp1 2.532708e-172 0.7564859 0.667 0.021 4.264066e-168  Mpzl1 1.676859e-170 1.3130816 0.986 0.060 2.823160e-166  Disp2 2.047403e-170 0.4188342 0.609 0.017 3.447008e-166  Cldn12 1.404723e-168 1.4705870 0.870 0.044 2.364992e-164  Pex1 6.932679e-168 1.0793341 0.855 0.041 1.167186e-163  Serpine2 6.873451e-167 2.3901991 0.681 0.023 1.157214e-162  Chst2 5.735701e-166 0.5031274 0.623 0.018 9.656625e-162  Lingo1 3.793237e-162 0.2586262 0.362 0.003 6.386294e-158  2510017J16Rik 2.917526e-161 0.6509473 0.565 0.015 4.911947e-157  Srgap3 4.696925e-161 0.7421138 0.797 0.037 7.907744e-157  Klhdc8a 2.196034e-159 0.7425108 0.725 0.029 3.697243e-155  Cd109 9.820392e-159 1.1114420 0.913 0.053 1.653361e-154  Ibsp 3.969527e-154 3.5842129 0.783 0.040 6.683096e-150  Adgrg3 7.684864e-154 0.7208587 0.667 0.024 1.293824e-149  Ank3 4.832928e-152 0.9390043 0.957 0.061 8.136718e-148  Gdpd5 6.715319e-152 1.3851501 0.928 0.061 1.130591e-147  Col7a1 1.589251e-151 1.1968641 0.957 0.061 2.675662e-147  Gm17590 3.523869e-151 0.2834897 0.377 0.004 5.932786e-147  Fzd1 7.727660e-151 1.5679986 0.826 0.046 1.301029e-146  1700109H08Rik 7.417603e-149 0.4306067 0.507 0.012 1.248828e-144  Akap12 7.940667e-149 1.1716861 0.739 0.034 1.336891e-144  Psrc1 2.015702e-148 0.3851642 0.420 0.007 3.393637e-144  Tspan9 1.247125e-146 0.8368776 0.884 0.054 2.099660e-142  Tspan8 3.589393e-142 0.4842769 0.493 0.013 6.043101e-138  Dennd2a 5.366566e-142 0.3552877 0.551 0.017 9.035151e-138  Grem1 3.327764e-141 1.6126170 0.841 0.052 5.602623e-137  Pdgfrb 3.882045e-138 0.5202530 0.565 0.019 6.535811e-134  Cavin3 5.009175e-138 0.4604431 0.725 0.034 8.433447e-134  Rbm48 2.581397e-136 1.1475196 0.855 0.053 4.346040e-132  Mcam 3.766162e-136 0.5586803 0.391 0.007 6.340711e-132  Ndst3 5.682472e-135 0.6516902 0.681 0.033 9.567010e-131  4833413G10Rik 1.541926e-134 0.4845746 0.493 0.014 2.595987e-130  Pxdn 1.672015e-133 1.4725472 0.942 0.073 2.815004e-129  Itga3 1.067514e-132 1.1228672 0.928 0.066 1.797267e-128  Ddr2 1.475235e-132 0.4378221 0.696 0.033 2.483706e-128  Rbpms2 2.173718e-132 0.9852336 0.855 0.058 3.659672e-128  D630045J12Rik 7.204487e-132 0.2777600 0.449 0.011 1.212947e-127  Cdh2 5.612466e-131 1.0730211 0.884 0.064 9.449147e-127  Gpc1 2.035809e-130 1.4260649 0.899 0.068 3.427488e-126  Nptx1 2.867170e-129 0.5298506 0.449 0.011 4.827167e-125  Agrn 1.694443e-128 0.6147046 0.696 0.036 2.852764e-124  Cst6 4.138385e-128 0.6266173 0.681 0.035 6.967386e-124  C1qtnf12 2.243517e-127 0.3289623 0.551 0.020 3.777185e-123  Atg9b 2.378931e-127 0.5252725 0.594 0.025 4.005168e-123  Col27a1 4.153947e-127 0.5089348 0.580 0.023 6.993586e-123  Adgrl2 7.582109e-127 0.5116929 0.435 0.011 1.276524e-122  Pros1 1.102005e-126 0.7340433 0.797 0.048 1.855335e-122  Cacna2d1 2.367485e-126 0.7617239 0.797 0.052 3.985898e-122  Ptprn 2.707732e-126 1.1451030 0.841 0.057 4.558738e-122  Nectin3 3.376276e-126 0.8382153 0.826 0.056 5.684298e-122  Col4a2 5.227043e-126 1.0980959 0.942 0.075 8.800250e-122  Samd5 5.855151e-125 0.2534229 0.348 0.006 9.857732e-121  Greb1 6.439267e-124 0.6418137 0.667 0.034 1.084115e-119  Ano1 1.657502e-122 1.5463865 0.971 0.085 2.790570e-118  Gpr149 1.313980e-121 0.2773102 0.348 0.006 2.212217e-117  Unc5b 3.103438e-121 1.1803327 0.928 0.076 5.224948e-117  Piezo2 4.632115e-121 1.8777119 0.957 0.084 7.798629e-117  Gprc5a 5.595960e-121 0.8458766 0.783 0.051 9.421357e-117  Zfp462 1.445562e-120 0.4733694 0.667 0.035 2.433749e-116  Nav3 4.951815e-120 0.6318918 0.797 0.054 8.336876e-116  Morc4 1.156045e-119 0.4349627 0.667 0.036 1.946318e-115  Plxna1 1.880869e-119 0.7811181 0.826 0.060 3.166631e-115  Cdr2l 2.507754e-119 0.4750741 0.696 0.039 4.222055e-115  Ltbp4 2.629938e-119 1.0146420 0.812 0.058 4.427763e-115  Col18a1 3.261583e-119 2.5227649 1.000 0.103 5.491202e-115  Loxl3 7.901678e-119 0.6913770 0.797 0.054 1.330327e-114  Plod2 1.626028e-118 1.7308475 1.000 0.098 2.737581e-114  Tinagl1 1.641555e-118 0.8436353 0.812 0.057 2.763722e-114  P3h3 6.272223e-118 0.4939922 0.536 0.021 1.055991e-113  **Cluster 10 Mesenchymal progenitors** |
| --- |
| cluster.markers <- FindMarkers(danx, ident.1 = 10, min.pct = 0.25)  \|++++++++++++++++++++++++++++++++++++++++++++++++++\| 100% elapsed=11s  > head(cluster.markers, n = 100)  p_val avg_log2FC pct.1 pct.2 p_val_adj  Birc5 3.753883e-132 2.3077276 0.742 0.041 6.320037e-128  Ccna2 3.603986e-105 1.2892541 0.470 0.018 6.067671e-101  Pclaf 1.498704e-97 2.6083450 0.682 0.052 2.523219e-93  Ube2c 2.790596e-93 2.1174373 0.576 0.036 4.698248e-89  Hist1h2ap 2.591653e-89 2.6884903 0.652 0.053 4.363307e-85  Cdca8 1.897902e-84 1.6313352 0.470 0.025 3.195309e-80  Hist1h1b 5.608513e-77 2.0283746 0.455 0.027 9.442493e-73  Hist1h2ae 3.672976e-76 2.0976746 0.545 0.042 6.183822e-72  Cdca3 9.502625e-74 1.5583323 0.424 0.024 1.599862e-69  Aurkb 1.723646e-72 1.1971640 0.333 0.013 2.901931e-68  Mki67 1.445663e-71 1.9728857 0.455 0.029 2.433918e-67  Top2a 4.136779e-70 2.2238921 0.667 0.073 6.964682e-66  Kif22 1.359009e-68 1.0382725 0.303 0.011 2.288028e-64  Nusap1 4.909682e-66 1.1640561 0.318 0.014 8.265941e-62  Cenpe 4.094797e-58 0.8608544 0.303 0.014 6.894000e-54  Knl1 6.534547e-57 0.9489863 0.318 0.017 1.100156e-52  Bub1 6.464861e-56 0.7508618 0.258 0.010 1.088424e-51  Tpx2 1.107190e-54 1.3155599 0.409 0.031 1.864066e-50  Stmn1 6.498378e-54 2.5791525 0.818 0.171 1.094067e-49  Spc24 1.299873e-48 0.9981040 0.333 0.023 2.188467e-44  Racgap1 5.481379e-48 1.3001905 0.455 0.046 9.228450e-44  Pbk 6.415074e-48 1.0693481 0.258 0.013 1.080042e-43  Kif11 4.265034e-46 0.9757336 0.258 0.014 7.180612e-42  Ccnb1 5.459131e-46 0.9032933 0.273 0.016 9.190993e-42  Kif15 1.478593e-44 0.8286229 0.273 0.016 2.489359e-40  Cenpm 3.283192e-43 0.6799864 0.258 0.015 5.527582e-39  Tk1 1.019845e-42 1.4378491 0.379 0.036 1.717012e-38  Ccnb2 5.159955e-42 0.8386548 0.273 0.017 8.687300e-38  Bub1b 1.134198e-41 0.8170572 0.258 0.016 1.909535e-37  Tyms 1.377777e-41 1.7654949 0.424 0.048 2.319626e-37  Cenpf 4.239479e-40 1.0059111 0.258 0.016 7.137587e-36  Kif23 4.406599e-37 1.0289847 0.364 0.037 7.418950e-33  Hmgb2 4.655295e-36 2.7193193 0.924 0.441 7.837655e-32  H2afz 3.778436e-34 2.1932427 0.970 0.629 6.361375e-30  Rrm2 2.646718e-29 1.0010551 0.288 0.030 4.456014e-25  Tuba1b 2.156312e-28 2.1070331 0.833 0.363 3.630366e-24  Cdk1 2.905147e-28 1.1111430 0.409 0.064 4.891106e-24  Bcl2a1a 4.674409e-27 1.5890567 0.500 0.097 7.869835e-23  Ptma 8.112307e-27 1.3195346 1.000 0.944 1.365788e-22  H2afx 1.509892e-26 1.3577150 0.485 0.098 2.542054e-22  Bcl2a1b 1.961235e-26 1.6118321 0.803 0.253 3.301934e-22  Smc2 3.227376e-25 1.2253800 0.424 0.078 5.433611e-21  Tubb5 5.887850e-25 1.7766304 0.909 0.575 9.912784e-21  C1qb 1.646363e-24 1.9018242 0.652 0.175 2.771817e-20  Cks2 5.136486e-24 1.4679807 0.561 0.141 8.647788e-20  Gmnn 9.395210e-24 1.0371835 0.424 0.080 1.581778e-19  Aif1 1.154331e-23 1.8347477 0.636 0.181 1.943431e-19  C1qa 1.209539e-23 1.7461773 0.667 0.183 2.036380e-19  Tmsb4x 1.847064e-23 1.1665860 1.000 0.999 3.109716e-19  Gatm 4.044414e-22 1.8730907 0.621 0.198 6.809176e-18  Bcl2a1d 5.998963e-22 1.2403591 0.455 0.096 1.009985e-17  Rrm1 2.597002e-21 1.3246373 0.455 0.102 4.372313e-17  Cxcl16 5.421460e-21 1.5169828 0.500 0.120 9.127570e-17  Msr1 1.035051e-20 1.3425079 0.455 0.100 1.742612e-16  Cenpw 1.122721e-20 0.8388917 0.303 0.047 1.890214e-16  Malat1 4.706924e-20 -2.5014918 0.348 0.831 7.924578e-16  Rfc5 6.352104e-20 0.8219691 0.288 0.044 1.069440e-15  Hist1h1e 6.866738e-20 1.3470422 0.485 0.123 1.156084e-15  Incenp 1.107681e-18 0.9051065 0.303 0.053 1.864891e-14  Lyz2 1.391515e-18 1.6150202 0.727 0.260 2.342755e-14  Junb 3.700952e-18 -2.4769801 0.379 0.803 6.230923e-14  C1qc 4.880997e-18 1.6059380 0.561 0.165 8.217646e-14  Hmgn2 1.301497e-17 1.3530090 0.561 0.184 2.191200e-13  Lig1 1.331136e-17 0.8019928 0.318 0.060 2.241101e-13  Slamf9 7.288100e-17 0.7921366 0.288 0.051 1.227024e-12  Fcer1g 1.077675e-16 1.3930470 0.727 0.271 1.814373e-12  Pf4 3.559068e-16 1.6432260 0.424 0.111 5.992048e-12  Atpif1 4.401824e-16 1.2807235 0.833 0.457 7.410911e-12  Ran 1.846479e-15 1.2745943 0.833 0.517 3.108732e-11  Arg1 2.649943e-15 0.9797746 0.470 0.129 4.461444e-11  Apoe 3.132128e-15 1.2094098 0.788 0.373 5.273251e-11  Rps27 7.303832e-15 -1.5579985 0.485 0.849 1.229673e-10  Apoc2 9.917725e-15 1.1834588 0.348 0.080 1.669748e-10  Jund 1.004742e-14 -1.8492103 0.318 0.773 1.691583e-10  Cox8a 1.567473e-14 0.9283069 0.955 0.831 2.638997e-10  Ppia 4.962612e-14 0.7894734 0.985 0.917 8.355053e-10  Ccl24 5.181960e-14 1.9767242 0.288 0.060 8.724348e-10  Klf2 6.068075e-14 -2.4356510 0.227 0.671 1.021621e-09  Tubb4b 6.393222e-14 1.2912429 0.591 0.246 1.076363e-09  Ybx1 2.379939e-13 1.1381948 0.879 0.666 4.006865e-09  Mcm3 3.252660e-13 0.7390529 0.333 0.081 5.476178e-09  Tyrobp 5.182101e-13 0.9211215 0.712 0.282 8.724585e-09  Cks1b 7.362173e-13 0.9860740 0.455 0.153 1.239495e-08  Anp32b 1.484673e-12 1.1777789 0.742 0.485 2.499595e-08  Actb 1.544930e-12 0.7983446 1.000 0.996 2.601044e-08  Mgl2 2.124704e-12 0.7859253 0.258 0.053 3.577152e-08  Prdx1 2.152922e-12 0.8891464 0.939 0.609 3.624660e-08  Slc25a5 2.910747e-12 1.1827808 0.788 0.551 4.900534e-08  Tmpo 3.331811e-12 1.1804920 0.621 0.297 5.609437e-08  Npl 4.127494e-12 0.9023949 0.273 0.060 6.949049e-08  Clec4n 4.785634e-12 0.8349504 0.409 0.119 8.057093e-08  Ccl12 4.791061e-12 1.2250966 0.303 0.073 8.066230e-08  1810037I17Rik 5.877583e-12 1.1257415 0.803 0.482 9.895499e-08  Dck 1.398086e-11 0.9412996 0.394 0.121 2.353817e-07  Hint1 1.475023e-11 0.9495056 0.864 0.643 2.483348e-07  Foxp1 3.507720e-11 -1.5888010 0.197 0.602 5.905597e-07  Cox5a 3.572184e-11 1.2086200 0.788 0.575 6.014129e-07  Pycard 3.851142e-11 1.2514387 0.561 0.248 6.483783e-07  Hmgb1 4.759003e-11 1.0910087 0.712 0.436 8.012258e-07  Ntpcr 9.674735e-11 1.0416145 0.394 0.134 1.628838e-06 |
