# Supplementary material for: CD4+ T Cells Sensitize Quasimesenchymal Breast Tumors Lacking CD73 to Anti-CTLA4 Immune Checkpoint Blockade Therapy
Source: Cancer Res Commun. 2026 Jun 2;6(6):1278–94. doi: 10.1158/2767-9764.CRC-26-0304 (PMC13227059; doi:10.1158/2767-9764.CRC-26-0304)
Supplement: Supplementary Table 2 — Genes present in each cluster depicted in Figure 5B [file crc-26-0304_supplementary_table_2_suppst2.docx]

**Supplementary Table 2: Genes present in each cluster depicted in Figure 5B**

| **Cluster** | **Identified Cell Type** | **Key Distinguishing Markers** |
| --- | --- | --- |
| **Stromal & Endothelial Cells** |  |  |
| Cluster 16 | Endothelial Cells | PLVAP, EGFL7, CD81, ENG (CD105), ESAM. |
| Cluster 24 | Cancer-Associated Fibroblasts (CAFs) | COL1A1, COL1A2, POSTN, BGN, SPARC, COL3A1 (extracellular matrix production). |
| **B Cells & Plasma Cells** |  |  |
| Cluster 6 | B Cells | CD79A, CD79B, MS4A1 (CD20), IGHM. |
| Cluster 17 | Naive B Cells | IGHD, IGHM, MS4A1, CD79A/B. |
| Cluster 21 | Plasma Cells | JCHAIN, MZB1, IGHG1, XBP1 (antibody secretion). |
| Cluster 22 | Germinal Center B Cells / Plasmablasts | AICDA, MZB1, POU2AF1, IRF8 (active somatic hypermutation/differentiation). |
| **Myeloid Cells** |  |  |
| Cluster 3 | Neutrophils / MDSCs | S100A8, S100A9, CXCR2, CXCL2 (Myeloid-Derived Suppressor Cells / PMNs). |
| Cluster 7 | Tumor-Associated Macrophages (TAMs) | LYZ2, CD68, APOE, CSF1R, AIF1. |
| Cluster 13 | C1q+ Macrophages / TAMs | C1QA, C1QB, C1QC, APOE, TREM2 equivalents (immunosuppressive TAMs). |
| Cluster 18 | Conventional Dendritic Cells (cDCs) | H2-AA, H2-AB1 (MHC-II), IRF8, CLEC10A, CST3. |
| Cluster 19 | Mature / Migratory DCs (mDCs) | CCR7, CCL22, FSCN1 (also known as LAMP3+ or regulatory DCs). |
| Cluster 23 | Monocytes | PLAC8, EAR2, LYZ2, CYBB. |
| **T Cells & Natural Killer (NK) Cells** |  |  |
| Cluster 1 | Cytotoxic CD8+ T Cells | CD8A, CD8B1, CTSW, NKG7, CCL5 (effector/cytotoxicity). |
| Cluster 4 | Naive / Memory CD4+ T Cells | TCF7, CD28, CD2, SLAMF6 (stem-like / memory states). |
| Cluster 5 | Proliferating T Cells | HMGB2, MKI67 correlates (TOP2A, RRM2) + T cell signatures (IL2RB, ICOS). |
| Cluster 8 | Naive T Cells | CCR7, LEF1, KLF2, SELL (classic naive markers). |
| Cluster 9 | NKT Cells / Effector T Cells | CXCR6, NKG7, KLRD1, KLRC1 + CD3G (mix of NK receptors and T cell markers). |
| Cluster 10 | Memory / Stem-like CD8+ T Cells | CD8A, CD8B1, TCF7, CCR7, CD27. |
| Cluster 11 | Regulatory T Cells (Tregs) | IKZF2 (Helios), CTLA4, TNFRSF4 (OX40), TNFRSF18 (GITR). |
| Cluster 14 | Natural Killer (NK) Cells | NCR1 (NKp46), NKG7, KLRD1, XCL1 (lack of CD3 markers). |
| **Epithelial / Tumor Cells** |  |  |
| Cluster 0 | Tumor Cells (EMT / Stromal-like) | KRT8, KRT18 (epithelial) alongside TIMP1, DCN, COL6A1 (stromal/EMT transition). |
| Cluster 2 | Proliferating Tumor Cells | UBE2C, BIRC5, CENPF (cell cycle/proliferation) + EPCAM, KRT8. |
| Cluster 12 | Luminal Tumor / Epithelial Cells | CLDN3, CLDN6, EPCAM, KRT8, KRT18. |
| Cluster 15 | Proliferating Tumor Cells (Basal-like) | TUBA1B, TUBB5, STMN1 (proliferation) + KRT14, KRT18 (basal/luminal mix). |
| Cluster 20 | Tumor Cells (SPP1+ / Hypoxic) | SPP1, TIMP1, KRT8, MSLN. SPP1 often marks hypoxic or inflammatory states. |
| Cluster 25 | Normal Luminal Epithelial Cells | PRLR, GATA3, EPCAM, AQP5. (GATA3 and PRLR denote hormone-responsive luminal identity). |
| Cluster 26 | Alveolar / Secretory Epithelial Cells | CSN3, MFGE8, LCN2, EPCAM. These denote milk-producing/alveolar properties. |
